# Discovery and prospects of new heterocyclic Isatin-hydrazide derivative with a novel role as estrogen receptor α degrader in breast cancer cells

**DOI:** 10.3389/fchem.2024.1424637

**Published:** 2024-07-03

**Authors:** Muhammad Nouman Arif, Sadia Sarwar, Farhat Firdous, Rahman Shah Zaib Saleem, Humaira Nadeem, Abir Abdullah Alamro, Amani Ahmad Alghamdi, Atekah Hazza Alshammari, Omer Farooq, Rashid Ali Khan, Amir Faisal

**Affiliations:** ^1^ Pharmaceutical Chemistry Research Lab, Department of Pharmaceutical Chemistry, Riphah Institute of Pharmaceutical Sciences, Riphah International University, Islamabad, Pakistan; ^2^ Cell Culture Laboratory, Department of Pharmacognosy, Riphah Institute of Pharmaceutical Sciences, Riphah International University, Islamabad, Pakistan; ^3^ Department of Chemistry and Chemical Engineering, Syed Babar Ali School of Science and Engineering, Lahore University of Management Sciences (LUMS), Defence Housing Authority, Lahore, Pakistan; ^4^ Department of Life Sciences, Syed Babar Ali School of Science and Engineering, Lahore University of Management Sciences (LUMS), Lahore, Pakistan; ^5^ Department of Biochemistry, College of Science, King Saud University, Riyadh, Saudi Arabia; ^6^ Shifa College of Pharmaceutical Sciences, Shifa Tameer-e-Millat University, Islamabad, Pakistan

**Keywords:** isatin–hydrazide derivatives, antiproliferative activity, molecular docking, molecular dynamics simulations, estrogen receptor, breast cancer

## Abstract

**Introduction:** Isatin, a heterocycle scaffold, is the backbone of many anticancer drugs and has previously been reported to engage multiple cellular targets and mechanisms, including angiogenesis, cell cycle, checkpoint pathways and multiple kinases. Here, we report that a novel isatin derivative, 5i, degrades estrogen receptor alpha (ERα) in estrogen-dependent breast cancer cells. This effect of the isatin nucleus has not been previously reported. Tamoxifen and fulvestrant represent standard therapy options in estrogen-mediated disease but have their own limitations. Isatin-based triple angiokinase inhibitor BIBF1120 (Nintedanib) and multikinase inhibitor Sunitinib (Sutent) have been approved by the FDA.

**Methods:** Keeping this in view, we synthesized a series of N'-(1-benzyl-2-oxo-1, 2-dihydro-3H-indol-3-ylidene) hydrazide derivatives and evaluated them *in vitro* for antiproliferative activities in MCF-7 (ER+) cell line. We further investigated the effect of the most potent compound (5i) on the Erα through Western Blot Analysis. We used *in silico* pharmacokinetics prediction tools, particularly pkCSM tool, to assess the activity profiles of the compounds.

**Results and discussion:** Compound 5i showed the best antiproliferative activity (IC50 value; 9.29 ± 0.97 µM) in these cells. Furthermore, 5i downregulated ERα protein levels in a dose-dependent manner in MCF-7. A multifaceted analysis of physicochemical properties through Data Warrior software revealed some prominent drug-like features of the synthesized compounds. The docking studies predicted the binding of ligands (compounds) with the target protein (ERα). Finally, molecular dynamics (MD) simulations indicated stable behavior of the protein-ligand complex between ERα and its ligand 5i. Overall, these results suggest that the new isatin derivative 5i holds promise as a new ERα degrader.

## 1 Introduction

Breast cancer is the leading malignancy causing the highest death rate among women (Guissi et al., 2017). Most BC patients have an estrogen receptor (ER)-positive form of the disease, with postmenopausal women accounting for 75% of the cases (Shoda et al., 2015). The initial line of treatment for ER-positive BC is endocrine therapy. Selective estrogen receptor modulators (SERMs) are a predominant class of endocrine therapeutic agents, including tamoxifen (brand name: Soltamox), raloxifene (brand name: Evista), and toremifene (brand name: Farestone). These pharmacological substances bind to the ER (ERα or ERβ subtypes) in cells, acting as agonists/antagonists in an organ-specific manner (Dandriyal et al., 2016). The first-generation SERM, tamoxifen, dramatically decreased BC mortality rates and the risk of recurrence. Although tamoxifen remained a gold standard in BC treatment, its use was restricted once its agonistic action on endometrial cells was shown to be associated with moderately increased risk of endometrial cancer growth, progression, and resistance (Duffy, 2006; Hiscox et al., 2006; Lewis-Wambi et al., 2011; Welsh et al., 2012; Han et al., 2016). Second-generation SERMs have also been associated with hot flashes, increased blood clot risk, deep vein thrombosis, and pulmonary embolism. Bazedoxifene, a third-generation SERM, was launched to treat BC and osteoporosis due to the toxicity and unfavorable side effects associated with the earlier SERMs (Lewis-Wambi et al., 2011; Payton-Stewart et al., 2014) by replacing the benzothiophene core of raloxifene by indole (Harris et al., 2003). Bazedoxifene binds to both ERα and ERβ, with a stronger affinity for the former. The inhibitory impact of bazedoxifene is linked to cell cycle arrest and ERα downregulation (Lewis-Wambi et al., 2011). Recently, bazedoxifene was used in combination with palbociclib to treat stage IV metastatic BC (Lindsay et al., 2009; Gupta et al., 2014; Jeselsohn et al., 2019).

Selective estrogen receptor degraders (SERDs), another class of ER-binding small molecules, bind to the ER and downregulate ER-mediated transcriptional activity. Fulvestrant and elacestrant are FDA-approved SERDs for clinical use in ER+ BC patients. Some small-molecule examples of SERDs have been disclosed in the literature (e.g., WO2005073204, WO2014205136, and WO2016097071). There has been a recent shift of focus in research on endocrine therapies toward SERDs as the development of resistance against SERMs grows (Bhatia et al., 2023). These SERDs can be used either as single agents or in combination with other classes of drugs, including SERMs, aromatase inhibitors, CDK4/CDK6 inhibitors, PI3K inhibitors, and mTOR inhibitors to treat hormone receptor-positive BC. The quest for new molecules to treat ER+ cancers that display better pharmacokinetic and pharmacodynamic properties, including oral bioavailability and higher efficiency in the clinic, is highly desirable.

**FIGURE 1:**
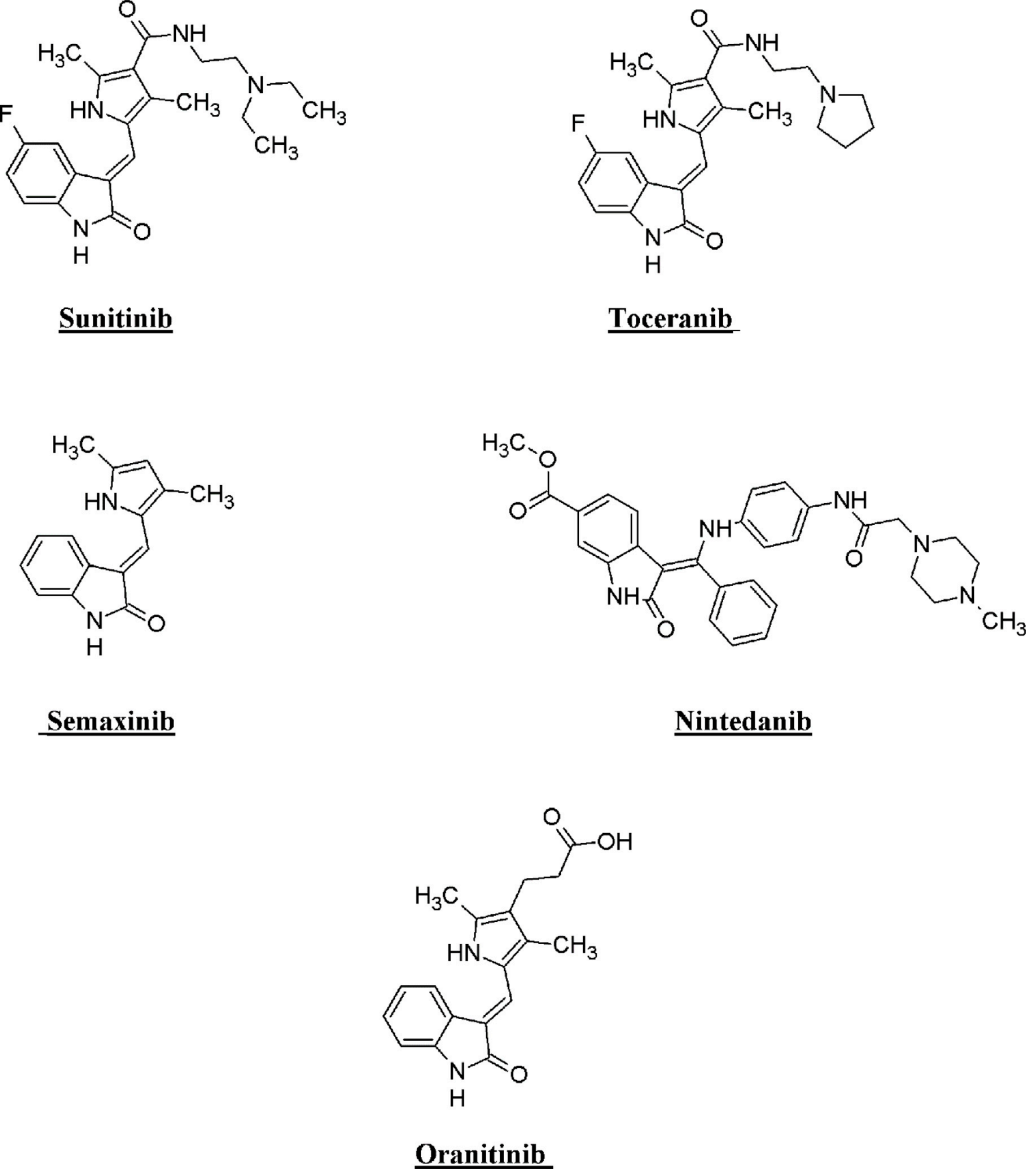
Structure of few isatin derivatives in clinical application/clinical trials.

Isatin stands out as an important class of heterocyclic compounds. It is a versatile scaffold present in human and other mammalian tissues. Isatin is a common structural feature in various dyes, agrochemicals, and pharmacologically active molecules. The synthetic ingenuity of isatin makes it a perfect platform for structural alterations and derivatization. Isatin-based compounds have displayed diverse biological properties, such as anticancer, antidepressant, anticonvulsant, antifungal, anti-HIV, and antiangiogenic activities (Pandeya et al., 1999; Sridhar and Ramesh, 2001; Verma et al., 2004; Selvam et al., 2008; Rohini et al., 2011; Ibrahim et al., 2015). Isatin–quinazoline hybrids have demonstrated antiproliferative activities against HepG2, MCF-7, and HT-29 cancer cell lines (Fares et al., 2015). Several isatin-based compounds have gone through clinical trials (shown in Figure 1), including triple angiokinase inhibitor BIBF1120 (Nintedanib, Vargatef), Sunitinib (Sutent), a multikinase inhibitor targeting VEGFR-1, VEGFR-2, PDGFRb and c-Kit. Sunitinib was approved by FDA for treating gastrointestinal stromal tumors (GIST) and advanced renal cell carcinoma (RCC) (Motzer et al., 2006; Le Tourneau et al., 2007).

 The main objective of the present research was to synthesize a new series of compounds based on the isatin nucleus and to investigate their antiproliferative activity and potential to degrade ER, inspired by the uniqueness of the isatin scaffold and its demonstrated biological activities. The present study describes the synthesis of N'-(1-benzyl-2-oxo-1, 2-dihydro-3H-indol-3-ylidene) hydrazide derivatives along with their antiproliferative evaluation against MCF-7 (ER+) cell-lines using MTT assay. Predictions of physicochemical and pharmacokinetic properties of synthesized compounds were conducted using *in silico* approaches. Molecular docking and molecular dynamics simulations further explored the binding modes of test compounds into the ligand binding domain (LBD) of estrogen receptor (ERα).

The main objective of the present research was to synthesize new series of an isatin-based nucleus and to investigate the candidates as potential ER degraders, inspired by the deficiencies and side effects of first, second, and third generations of endocrine therapeutic agents for the treatment of ER+ BC. Thus, inspired by the uniqueness of the isatin scaffold and its demonstrated biological activities, this study was aimed at the synthesis of new compounds that will have even better pharmacokinetics and pharmacodynamics profiles than the current regimen.

The present study describes the synthesis of Nʹ-(1-benzyl-2-oxo-1, 2-dihydro-3H-indol-3-ylidene) hydrazide derivatives, along with their antiproliferative evaluation against MCF-7 (ER+) cell lines using the MTT assay. The physicochemical and pharmacokinetic properties of synthesized compounds were predicted using *in silico* approaches. Molecular docking and molecular dynamics (MD) simulations further explored the binding modes of the test compounds into the ligand-binding domain (LBD) of estrogen receptor alpha (ERα).

## 2 Materials and methods

### 2.1 Synthetic chemistry

Solvents and chemicals from Merck and Sigma-Aldrich were used without further purification. All the synthesized compounds were purified by recrystallization in suitable solvents. The boiling points of the esters were determined using a microscale condenser apparatus. Elemental analyses (C, H, and N) were in agreement with the proposed structures and within ±0.5% of the theoretical values. Thin-layer chromatography (TLC) was used to monitor the reaction progress and purity of the final products using silica gel-precoated aluminum sheets (60 F254; Merck Schuchardt, Darmstadt, Germany). The TLC plates were visualized with ultraviolet light at 365 and 254 nm. The synthesized compounds were characterized using spectrophotometric analysis employing Fourier-transform infrared (FTIR) spectroscopy (Nicolet iS10 spectrophotometer, Thermo Fisher Scientific) and ^1^H NMR and ^13^C NMR spectroscopy (Bruker AM-300 spectrophotometer).

#### 2.1.1 Preparation of isatin (1H-indole-2,3-dione) (**1**)

The synthesis of isatin involved the reaction of chloral hydrate, hydroxylamine, and aniline to yield α-isonitrosoacetanilide and subsequent electrophilic cyclization in the presence of a strong acid such as concentrated sulfuric acid. The synthesis is generally known as the Sandmeyer isatin synthesis (Sumpter, 1944). A yield of 84.7% was obtained, with m.p. 200°C and R_f_ 0.85 (ethyl acetate:petroleum ether, 2:1).

#### 2.1.2 Synthesis of N-benzyl indole-2,3-dione (N-benzyl isatin) (**2**)

In a round bottom flask, indole-2,3-dione (**1**, 0.8 gm, 3.37 mM) and an equimolar quantity of benzyl chloride (6.5 mL, 3.7 mM) were mixed in DMF (20 mL). K_2_CO_3_ (2 g) was added to this mixture, and the mixture was refluxed for 2 h. The flask was cooled, and the contents of the flask were poured onto ice-cold water (100 mL), leading to the formation of an orange–red precipitate, which was collected, washed with water, and dried. The compound was then purified by recrystallization from acetonitrile. A yield of 83.6% was obtained, with m.p. 134°C and R_f_ 0.65 (ethyl acetate:petroleum ether, 2:1); ^1^H NMR: (300 MHz, CDCl_3_) δ 5.09 (s, 2H, CH_2_ benzyl group), 7.18 (d, J = 7.8 Hz, 1H, Ar-H), 7.23 (t, J = 7.6 Hz, 1H, Ar-H), 7.41 (s, 5H, Ar-H), 7.52 (t, J = 8.4 Hz, 1H, Ar-H), and 7.69 (d, J = 7.9 Hz, 1H, Ar-H). The NMR spectral data are congruent with previously documented findings (El-Faham et al., 2014).

#### 2.1.3 General procedure for the preparation of esters 3(a–i)

Appropriately substituted benzoic acid (0.032 mol) was dissolved in absolute ethanol (20 mL) in a round bottom flask. Concentrated sulfuric acid (1 mL) was added to the reaction mixture, and the mixture was refluxed. The reaction progress was monitored through TLC. Upon completion of the reaction, the mixture was cooled and poured onto ice-cold water, and the product was extracted with ethyl acetate. The ethyl acetate layer was washed with water and 10% NaHCO_3_ solution, dried over anhydrous sodium sulfate, and evaporated to dryness to obtain the respective ester (Guzowski Jr et al., 2012). The NMR spectral data of all synthesized ester derivatives 3(a–i) are congruent with previously documented findings (Samanta et al., 2014).

*Methyl pyridine-4-carboxylate (3a)*: Yield, 75%; b.p., 207°C–208°C; and IR (KBr) cm^-1^, 1,720 (C=O). ^1^H NMR: (300 MHz, CDCl_3_) δ: 3.74 (s, 3H, OCH_3_), 7.91 (d, J = 8.4 Hz, 2H, Ar-H), and 8.73 (d, J = 7.8 Hz, 2H, Ar-H).

*Methyl 3-bromobenzoate (3b)*: Yield, 68%; b.p., 233°C–237°C; and IR (KBr) cm^-1^, 1,729 (C=O). ^1^H NMR: (300 MHz, CDCl_3_) δ: 3.84 (s, 3H, OCH_3_), 7.62–7.68 (m, 1H, Ar-H), 7.73–7.79 (m, 1H, Ar-H), and 7.82 (d, J = 7.8 Hz, 2H, Ar-H).

*Methyl 3-iodobenzoate (3c)*: Yield, 65%; b.p., 263°C–267°C; and IR (KBr) cm^-1^, 1,730 (C=O). ^1^H NMR: (300 MHz, CDCl_3_) δ: 3.86 (s, 3H, OCH_3_), 7.68–7.77 (m, 1H, Ar-H), 7.82–7.86 (m, 1H, Ar-H), and 7.95 (d, J = 7.8 Hz, 2H, Ar-H).

*Methyl 2-hydroxybenzoate (3d):* Yield, 75%; b.p., 214°C–216°C; and IR (KBr) cm^-1^, 1,720 (C=O). ^1^H NMR: δ: 3.83 (s, 3H, OCH_3_), 5.29 (s, 1H of OH), 7.05 (d, J = 8.3 Hz, 1H, Ar-H), 7.83 (d, J = 8.1 Hz, 1H, Ar-H), 7.85 (d, J = 8.3 Hz, 1H, Ar-H), and 7.94 (d, J = 8.1 Hz, 1H, Ar-H).

*Methyl 4-fluorobenzoate (3e):* Yield, 75%; b.p., 196°C–198°C; and IR (KBr) cm^-1^, 1,720 (C=O). ^1^H NMR: (300 MHz, CDCl_3_) δ: 3.83 (s, 3H, OCH_3_), 7.50 (d, J = 7.8 Hz, 2H, Ar-H), and 7.83 (d, J = 7.8 Hz, 2H, Ar-H).

*Methyl pyridine-2-carboxylate (3f):* Yield, 75%; b.p., 204°C–206°C; and IR (KBr) cm^-1^, 1,720 (C=O). ^1^H NMR: (300 MHz, CDCl_3_) δ: 3.72 (s, 3H, OCH_3_), 7.57–7.60 (m, 1H, Ar-H), 7.62 (d, J = 7.8 Hz, 2H, Ar-H), and 7.65–7.69 (m, 1H, Ar-H).

*Methyl 3,4,5-trihydroxybenzoate (3g)*: Yield, 67%; b.p, 201°C–203°C; and IR (KBr) cm^-1^, 1,725 (C=O). ^1^H NMR: (300 MHz, CDCl_3_) δ: 3.87 (s, 3H, OCH_3_), 5.34 (m, 3H of OH), and 7.25 (d, J = 7.2, 2H, Ar-H).

*Methyl 2-chlorobenzoate (3h):* Yield, 60%; b.p, 217°C–219°C; and IR (KBr) cm^-1^, 1,725 (C=O). ^1^H NMR: (300 MHz, CDCl_3_) δ: 3.81 (s, 3H, OCH_3_), 7.35 (d, J = 8.5 Hz, 1H, Ar-H), 7.43 (d, J = 8.3 Hz, 1H, Ar-H), 7.57 (d, J = 8.5 Hz, 1H, Ar-H), and 7.94 (d, J = 8.3 Hz, 1H, Ar-H).

*Methyl benzoate (3i):* Yield, 75%; b.p., 188°C–190°C; and IR (KBr) cm^-1^, 1,725 (C=O). ^1^H NMR: (300 MHz, CDCl_3_) δ: 3.81 (s, 3H, OCH_3_), 7.45 (d, J = 8.5 Hz, 2H, Ar-H), 7.57–7.61 (m, 1H, Ar-H), and 8.02 (d, J = 8.5 Hz, 2H, Ar-H).

#### 2.1.4 General procedure for the preparation of hydrazide 4(a–i)

Respective ester **3(a–i)** was dissolved in absolute ethanol (50 mL), and hydrazine monohydrate (15 mL) was added to the solution. The mixture was thoroughly stirred and heated under reflux for 10–12 h. The reaction progress was monitored through TLC (chloroform:methanol, 3:1). Upon completion of the reaction, excess ethanol and hydrazine were evaporated under reduced pressure, yielding the corresponding hydrazide.

The NMR spectral data are congruent with previously documented findings (Khan et al., 2003).

*Pyridine-4-carbohydrazide (4a)*: Light yellow solid; yield, 80%; m.p., 169°C–171°C; and IR (KBr) cm^-1^, 1,675 (C=O). ^1^H NMR: (300 MHz, CDCl_3_) δ: 4.45 (s, 2H, NH_2_), 7.88 (d, J = 8.5 Hz, 2H, Ar-H), 8.34 (d, J = 8.5 Hz, 2H, Ar-H), and 9.53 (s, 1H, NH).

*3-Bromobenzohydrazide (4b):* White solid; yield, 75%; m.p., 183°C–187°C; and IR (KBr) cm^-1^, 1,650 (C=O). ^1^H NMR: (300 MHz, CDCl_3_) δ: 4.51 (s, 2H, NH_2_), 7.43–7.48 (m, 1H, Ar-H), 7.65–7.71 (m, 1H, Ar-H), 7.94 (d, J = 7.4 Hz, 2H, Ar-H), and 9.60 (s, 1H, NH).

*3-Iodobenzohydrazide (4c):* White solid; yield, 70%; m.p., 208°C–211°C; and IR (KBr) cm^-1^, 1,645 (C=O). ^1^H NMR: (300 MHz, CDCl_3_) δ: 4.49 (s, 2H, NH_2_), 7.26–7.31 (m, 1H, Ar-H), 7.42–7.47 (m, 1H, Ar-H), 8.08 (d, J = 8.2 Hz, 2H, Ar-H), and 10.02 (s, 1H, NH).

*2-Hydroxybenzohydrazide (4d):* Light yellow solid; yield, 80%; m.p., 146°C–148°C; and IR (KBr) cm^-1^, 1,635 (C=O). ^1^H NMR: (300 MHz, CDCl_3_) δ: 4.49 (s, 2H, NH_2_), 5.41 (s, 1H of OH), 7.03 (d, J = 8.6 Hz, 1H, Ar-H), 7.22 (d, J = 8.3 Hz, 1H, Ar-H), 7.52 (d, J = 8.6 Hz, 1H, Ar-H), 7.89 (d, J = 8.3 Hz, 1H, Ar-H), and 10.02 (s, 1H, NH).

*4-Fluorobenzohydrazide (4e):* White solid; yield, 70%; m.p., 176°C–178°C; IR (KBr) cm^-1^, 1,644 (C=O). ^1^H NMR: (300 MHz, CDCl_3_) δ: 4.39 (s, 2H, NH_2_), 7.47 (d, J = 8.4 Hz, 2H, Ar-H), 7.81 (d, J = 8.4 Hz, 2H, Ar-H), and 9.97 (s, 1H, NH).

*Pyridine-2-carbohydrazide (4f)*: Light yellow solid; yield, 70%; m.p., 164°C–166°C; and IR (KBr) cm^-1^, 1,670 (C=O). ^1^H NMR: (300 MHz, CDCl_3_) δ: 4.43 (s, 2H, NH_2_), 7.57–7.63 (m, 1H, Ar-H), 7.71 (d, J = 8.0 Hz, 2H, Ar-H), 8.52–7.69 (m, 1H, Ar-H), and 9.87 (s, 1H, NH).

*3,4,5-Trihydroxybenzohydrazide (4g)*: Light brown solid; yield, 55%; m.p., 159°C–161°C; and IR (KBr) cm^-1^, 1,665 (C=O). 1H NMR: (300 MHz, CDCl_3_) δ: 4.36 (s, 2H, NH_2_), 5.51 (m, 3H of OH), 7.17 (d, J = 7.4, 2H, Ar-H), and 10.19 (s, 1H, NH).

*2-Chlorobenzohydrazide (4h)*: White solid; yield, 75%; m.p., 114°C–116°C; and IR (KBr) cm^-1^, 1,647 (C=O). ^1^H NMR: (300 MHz, CDCl_3_) δ: 4.35 (s, 2H, NH_2_), 7.47 (d, J = 8.8 Hz, 1H, Ar-H), 7.59 (d, J = 8.4 Hz, 1H, Ar-H), 7.66 (d, J = 8.8 Hz, 1H, Ar-H), 8.94 (d, J = 8.4 Hz, 1H, Ar-H), and 9.99 (s, 1H, NH).

*Benzohydrazide (4i)*: White solid; yield, 75%; m.p., 109°C–111°C; and IR (KBr) cm^-1^, 1,667 (C=O). ^1^H NMR: (300 MHz, CDCl_3_) δ: 4.49 (s, 2H, NH_2_), 7.45 (d, J = 8.5 Hz, 2H, Ar-H), 7.55–7.58 (m, 1H, Ar-H), 7.77 (d, J = 8.5 Hz, 2H, Ar-H), and 10.21 (s, 1H, NH).

#### 2.1.5 N'-(1-benzyl-2-oxo-1, 2-dihydro-3H-indol-3-ylidene) hydrazide derivatives (5a–i)

A solution of appropriate hydrazide **4(a–i)** (316 mg, 2 mmol) was prepared in ethanol (20 mL). Another solution was prepared containing benzylisatin (**2**) (1 mmol) in ethanol (20 mL) and glacial acetic acid (two drops). The two solutions were mixed and refluxed for 3–4 h. Upon completion of the reaction, the mixture was cooled, and the product was filtered and purified by recrystallization from ethanol.

*N'-*(*1-benzyl-2-oxo-1,2-dihydro-3H-indol-3-ylidene*)*pyridine-4-carbohydrazide (5a):* Yellowish orange; 89% yield; m.p., 182°C–184°C. IR (KBr) cm^-1^: 3,227 (NH), 1,612 (C=N), 1,713, 1,682 (amide C=O). ^1^H NMR (300 MHz, CDCl_3_) δ: 14.32 (s, 1H, NH of hydrazide), 8.77 (s, 2H, Ar-H), 8.31–8.25 (m, 1H, Ar-H), 7.74 (d, 2H, *J* = 6.4 Hz, Ar-H), 7.58 (t, 1H, Ar-H), 7.57–7.43 (m, 2H, Ar-H), 7.42–7.27 (m, 4H, Ar-H), 7.24–7.17 (m, 1H, Ar-H), and 5.22 (s, 2H, CH_2_ of the benzyl group). ^13^C NMR: δ: 170.46, 164.71, 151.17, 141.39, 139.65, 136.80, 136.77, 129.86, 128.76, 127.50, 127.40, 125.73, 122.82, 121.49, 120.89, 109.95, and 53.38. GC–MS (EI) *m/z* 356 [M]^+^. Elemental analysis: C_21_H_16_N_4_O_2_, calculated: C (70.77%), H (4.53%), N (15.72%), and O (8.98%); found: C (70.71%), H (4.51%), N (15.69%), and O (8.88%).

*N'-(1-benzyl-2-oxo-1,2-dihydro-3H-indol-3-ylidene)-3-bromobenzohydrazide (5b):* Yellowish orange; 84% yield; m.wt: 434.3. m.p., 186°C–190°C. IR (KBr) cm^-1^: 3,225 (NH), 1,617 (C=N), 1,710, 1,679 (amide C=O); ^1^H NMR (300 MHz, CDCl_3_) δ: 13.96 (s, 1H, NH of hydrazide), 8.30–8.20 (m, 1H, Ar-H), 8.05 (s, 1H, Ar-H), 7.85–7.71 (m, 1H, Ar-H), 7.62 (t, 1H, J = 6.2 Hz, Ar-H), 7.57 (d, 1H, J = 4.3 Hz, Ar-H), 7.45–7.40 (m, 3H, Ar-H), 8.38–8.27 (m, 5H, Ar-H), and 5.18 (s, 2H, CH_2_ benzyl group). ^13^C NMR: δ: 167.47, 162.82, 153.60, 142.25, 136.77, 134.46, 133.86, 132.14, 130.87, 128.86, 128.76, 127.80, 127.40, 126.83, 125.73, 122.82, 122.26, 118.93, 114.60, and 46.77. GC–MS (EI) *m/z* 433 [M]^+^. Elemental analysis: C_22_H_16_BrN_3_O_2_; calculated: C (60.84%), H (3.71%), Br (18.40%), N (9.68%), and O (7.37%); found: C (60.79%), H (3.73%), Br (18.33%), N (9.64%), and O (7.35%).

*N'-(1-benzyl-2-oxo-1,2-dihydro-3H-indol-3-ylidene)-3-iodobenzohydrazide (5c):* Yellowish orange; 77% yield; m.p., 201°C–203°C. IR (KBr) cm^-1^: 3,238 (NH), 1,615 (C=N), 1,717, 1,678 (amide C=O). ^1^H NMR (300 MHz, CDCl_3_) δ: 13.91 (s, 1H, NH of hydrazide), 8.31–8.20 (m, 1H, Ar-H), 8.12 (s, 1H, Ar-H), 7.60 (d, 1H, J = 6.2 Hz, Ar), 7.58–7.53 (m, 2H, Ar-H), 7.43–7.38 (m, 2H, Ar-H), 7.35–7.24 (m, 6H, Ar-H), and 5.18 (s, 2H, CH_2_ benzyl group). ^13^C NMR: δ: 165.18, 160.45, 140.46, 138.96, 138.42, 136.70, 135.34, 135.24, 131.14, 129.72, 128.76, 127.80, 127.34, 126.68, 125.06, 122.63, 120.43, 110.74, 93.74, and 43.64. GC–MS (EI) *m/z* 481 [M]^+^. Elemental analysis: C_22_H_16_IN_3_O_2_; calculated: C (54.90%), H (3.35%), I (26.37%), N (8.73%), and O (6.65%); found: C (54.88%), H (3.30%), I (26.37%), N (8.68%), and O (6.61%).

*N'-(1-benzyl-2-oxo-1,2-dihydro-3H-indol-3-ylidene)-2-hydroxybenzohydrazide (5d):* Yellowish orange; 78% yield; m.p., 193°C–195°C. IR (KBr) cm^-1^: 3,233 (NH), 1,623 (C=N), 1,726, 1,677 (amide C=O). ^1^H NMR (300 MHz, CDCl_3_) δ: 14.07 (s, 1H, NH of hydrazide), 8.31–8.20 (m, 1H, Ar-H), 7.80–7.78, (d, 2H, J = 8.1 Hz, Ar-H), 7.62–7.52 (m, 1H, Ar-H), 7.43–7.40 (m, 2H, Ar-H), 7.36–7.26 (m, 5H, Ar-H), 6.95–6.93 (m, 2H of Ar-H), 5.63 (s, 1H of OH), and 5.07 (s, 2H, CH_2_ benzyl group). ^13^C NMR: δ: 166.84, 162.11, 156.99, 138.81, 136.96, 136.77, 134.11, 130.08, 129.86, 128.76, 127.80, 127.40, 125.73, 122.82, 120.89, 119.58, 117.18, 116.09, 111.86, and 45.51. GC–MS (EI) *m/z* 371 [M]+. Elemental analysis: C_22_H_17_N_3_O_3_; calculated: C (71.15%), H (4.61%), N (11.31%), and O (12.92%); found: C (71.05%), H (4.55%), N (11.29%), and O (12.90%).

*N'-(1-benzyl-2-oxo-1,2-dihydro-3H-indol-3-ylidene)-4-fluorobenzohydrazide (5e):* Yellowish orange; 74% yield; m.p., 185°C–188°C. IR (KBr) cm^-1^: 3,239 (NH), 1,620 (C=N), 1,719, 1,685 (amide C=O). ^1^H NMR (300 MHz, CDCl_3_) δ: 14.16 (s, 1H, NH of hydrazide), 8.51–8.46 (m, 1H, Ar-H), 7.97 (t, 2H, J = 7.2 Hz, Ar-H), 7.63–7.52 (m, 1H, Ar-H), 7.41–7.36 (m, 3H, Ar-H), 7.34–7.29 (m, 6H, Ar-H), and 5.18 (s, 2H, CH_2_ benzyl group). ^13^C NMR: δ: 165.88, 164.04, 163.90, 163.51, 138.81, 136.80, 136.77, 130.98, 130.96, 130.65, 130.58, 129.86, 128.76, 127.80, 127.40, 125.73, 122.82, 120.89, 116.13, 115.95, 111.96, and 44.52. GC–MS (EI) *m/z* 373 [M]^+^. Elemental analysis: C_22_H_16_FN_3_O_2_; calculated: C (70.77%), H (4.32%), F (5.09%), N (11.25%), and O (8.57%); found: C (70.71%), H (4.29%), F (5.09%), N (11.22%), and O (8.53%).

*N'-*(*1-benzyl-2-oxo-1,2-dihydro-3H-indol-3-ylidene*)*pyridine-2-carbohydrazide (5f):* Yellowish orange; 86% yield; m.p., 187°C–189°C. IR (KBr) cm^-1^: 3,198 (NH), 1,631 (C=N), 1,707, 1,680 (amide C=O). ^1^H NMR (300 MHz, CDCl_3_) δ: 14.17 (s, 1H, NH of hydrazide), 8.67–8.66 (m, 1H, Ar-H), 8.30–8.21 (m, 1H, Ar-H), 8.03 (d, 1H, J = 8.3 Hz, Ar-H), 7.89–7.63 (m, 1H, Ar-H), 7.58–7.53 (m, 1H, Ar-H), 7.43–7.37 (m, 3H, Ar-H), 7.34–7.24 (m, 5H, Ar-H), and 5.15 (s, 2H, CH_2_ benzyl group). ^13^C NMR: δ: 163.95, 159.39, 149.99, 149.62, 138.81, 137.76, 136.96, 136.77, 129.86, 128.76, 127.80, 127.40, 125.94, 125.61, 123.75, 122.82, 120.89, 111.96, and 44.52. GC–MS (EI) *m/z* 356 [M]^+^. Elemental analysis: C_21_H_16_N_4_O_2_; calculated: C (70.77%), H (4.53%), N (15.72%), and O (8.98%); found: C (70.74%), H (4.51%), N (15.66%), and O (8.91%).

*N'-(1-benzyl-2-oxo-1,2-dihydro-3H-indol-3-ylidene)-3,4,5trihydroxybenzohydrazide (5g):* Yellowish orange; 72% yield; m.p., 204°C–206°C. IR (KBr) cm^-1^: 3,230 (NH), 1,619 (C=N), 1,714, 1,674 (amide C=O); ^1^H NMR (300 MHz, CDCl_3_) δ: 14.10 (s, 1H, NH of hydrazide), 8.30–7.20 (m, 1H, Ar-H), 7.62–7.53 (m, 1H, Ar-H), 7.43–7.38 (m, 2H, Ar-H) 7.36–7.24 (m, 5H, Ar-H), 7.15 (s, 2H of OH), 7.01 (s, 2H of Ar-H), 5.40 (s, 1H of OH), and 5.18 (s, 2H, CH_2_ benzyl group). ^13^C NMR: δ: 167.94, 162.11, 146.73, 138.81, 138.55, 136.82, 136.77, 129.86, 128.76, 127.80, 127.40, 126.88, 125.73, 122.82, 120.82, 113.25, 105.58, and 44.52. GC–MS (EI) *m/z* 403 [M]^+^. Elemental analysis: C_22_H_17_N_3_O_5_; calculated: C (65.5%), H (4.25%), N (10.42%), and O (19.83%); found: C (65.11%), H (4.22%), N (10.38%), and O (19.79%).

*N'-(1-benzyl-2-oxo-1,2-dihydro-3H-indol-3-ylidene)-2-chlorobenzohydrazide (5h):* Yellowish orange; 70% yield; m.p., 197°C–200°C. IR (KBr) cm^-1^: 3,237 (NH), 1,604 (C=N), 1,723, 1,683 (amide C=O); ^1^H NMR (300 MHz, CDCl_3_): δ: 14.26 (s, 1H, NH of hydrazide), 8.04–7.98 (m, 1H, Ar-H), 7.88–7.82 (t, 1H, J = 7.3 Hz, Ar) 7.58–7.42 (m, 1H, Ar), 7.38–7.36 (m, 4H, Ar-H), 7.34–7.32 (m, 6H, Ar-H), and 4.96 (s, 2H, CH_2_ benzyl group). ^13^C NMR: δ: 169.20, 162.58, 149.27, 140.52, 136.88, 136.77, 133.35, 132.58, 131.59, 129.98, 129.86, 129.53, 128.76, 127.80, 127.58, 127.40, 125.73, 122.82, 111.96, and 48.74. GC–MS (EI) *m/z* 389 [M]^+^. Elemental analysis: C_22_H_16_ClN_3_O_2_, calculated: C (67.78%), H (4.14%), Cl (9.09%), N (10.78%) and O (8.21%); found: C (67.71%), H (4.09%), Cl (9.04%), N (10.72%), and O (8.18%).

*N'-*(*1-benzyl-2-oxo-1,2-dihydro-3H-indol-3-ylidene*) *benzohydrazide (5i):* Yellowish orange; 73% yield; m.p., 188°C–190°C. IR (KBr) cm^-1^: 3,196 (NH), 1,606 (C=N), 1,721, 1,681 (amide C=O). ^1^H NMR (300 MHz, CDCl_3_) δ: 14.10 (s, 1H, NH of hydrazide), 8.31–8.25 (m, 1H, Ar-H), 7.94–7.90 (m, 2H, Ar-H), 7.62–7.51 (m, 4H, Ar-H), 7.49–7.43 (m, 2H, Ar-H), 7.38–7.24 (m, 5H, Ar-H), and 5.10 (s, 2H, CH_2_ benzyl group). ^13^C NMR: δ: 168.81, 163.66, 151.87, 141.39, 136.77, 135.96, 132.14, 129.86, 128.76, 128.68, 128.19, 127.80, 127.40, 125,73, 122.82, 120.89, 112.24, and 51.25. GC–MS (EI) *m/z* 355 [M]^+^. Elemental analysis: C_22_H_17_N_3_O_2_, calculated: C (74.35%), H (4.82%), N (11.82%), and O (9.00%); found: C (74.32%), H (4.79%), N (11.77%), and O (8.97%).

### 2.2 Cytotoxicity assay

The impact of novel derivatives was determined in the MCF-7 BC cell line using the sulforhodamine B (SRB) assay, as described previously at the Department of Life Sciences, Syed Babar Ali School of Science and Engineering, Lahore University of Management Sciences (LUMS), Lahore, Pakistan. The cell line was originally from ATCC and was validated through STR profiling by Microsynth AG. The cell line used in this study was obtained from ATCC^®^ HTB-22™. In this procedure, cells were plated in 96-well plates and subjected to different concentrations of derivatives (5a–5i) for 72 h. Subsequently, the cells were fixed by treatment with 3% ice-cold trichloroacetic acid (TCA) at 4°C for 2 h. Following fixation, the cells were washed and stained with 0.06% SRB for 30 min at room temperature. The SRB bound to the cells was then dissolved in 100 μL of Tris buffer (10 mM) at pH 10.5. The optical density (OD) was measured at 490 nm using a microplate reader (BioTek), and the percentage viability was calculated with reference to DMSO. The data represent the average from three independent experiments (Manzoor et al., 2018).

### 2.3 Immunoblotting

BC cells (MCF-7) were subjected to treatment with three different concentrations of **5i** (1.25X, 2.5X, and 5X of IC_50_) for 24 h. Afterward, cell lysis was performed with lysis buffer (50 mM NaCl, 20 mM Tris, pH 7.5, 1 mM EDTA, 1% Triton, 50 mM NaF supplemented with protease, and phosphatase inhibitors). The resulting cell lysates were cleared by centrifugation, and the protein concentration was determined using the Bradford reagent. An equal amount of proteins for each sample was separated by 10% SDS-PAGE and transferred to nitrocellulose membranes. These membranes were then blocked with skimmed milk and incubated with ERα and GAPDH (Millipore) antibodies. Following the incubation period, the membranes were washed with PBST and then exposed to a secondary antibody labeled with HRP for 1 h. The blots were subsequently washed again and developed using the ECL reagent using the Bio-Rad ChemiDoc system (Firdous et al., 2023).

### 2.4 Chemical space, drug-likeness prediction, and pharmacokinetics profiling of compounds 5a–5i

The chemical space and drug-like potential of the synthesized compounds were studied using DataWarrior (Sander et al., 2015), which is an open-source cheminformatics tool. The physicochemical properties of these compounds were evaluated using the cutoff criteria proposed by Lipinski (2004) and Veber et al. (2002). Lead-like molecules with favorable bioavailability typically exhibit the following characteristics: (a) molecular weight <500 g/mol; (b) ≤5 hydrogen bond donors; (c) ≤10 hydrogen bond acceptors; and (d) log(octanol/water) partition coefficient ≤5. In addition to the other requirements, Veber introduced the related issues of the number of rotatable bonds (10 or less) and the polar surface area (≤140 A2). The use of these parameters as a reference value was thus justified in the determination. To gain an understanding of the pharmaceutical properties of compounds 5a–5i, their structures were analyzed using pkCSM (Pires et al., 2015), a graph-based structural signature modeling server. At the beginning, ligand-based ADMET predictions were made to assist the structure screening using molecular docking and MD simulations with their information.

### 2.5 Assessment of toxicity *via* Toxtree software

Toxtree, an open-source software, was used on a Windows platform to predict the toxicity of the compounds. The software processed chemical structures by interacting with established toxic compounds, enabling predictive toxicology evaluations. Molecular structures were imported in MOL file format into the Toxtree navigator, subsequently undergoing processing via diverse interacting software modules. These modules encompassed Cramer’s rule, carcinogenicity, *in vitro* mutagenicity, skin corrosion/irritation, eye corrosion/irritation, cytochrome P450-mediated drug metabolism, and structure alerts for the *in vivo* micronucleus assay in rodents. Post-processing, comprehensive decision support was obtained in portable document format (Patlewicz et al., 2008).

### 2.6 Molecular docking analysis

The crystal structure of ERα (PDB ID: 3ERT) was analogized from the RCSB Protein Data Bank. The formation of nine distinct ligands, which were the derivatives of the isatin nucleus, was achieved using ChemSketch (version 12.0). Gridding parameters were determined using AutoDock Tools (v 1.5.6). Enhancements included the fusion of non-polar hydrogen; Gasteiger partial charges were added, and rotatable bonds were assigned. The spacing of 0.375 Å between the grid box dimensions included in the receptor site. After these preparations, the docking protocols were validated through a re-docking procedure using the ERα crystallographic structure (PDB ID: 3ERT). This process implied the docking of ligands into the active site of ERα, followed by the comparison of the resultant poses to the original crystallographic structure. In addition, The re-docked ligand had binding mode and interactions similar to the those of the original crystallographic structure. The actual protein–ligand docking was carried out using AutoDock Vina, and Open Babel GUI, Discovery Studio 4.1 Visualizer, and PyMOL were used for the preparation and conversion of the protein molecule into its structures by eliminating ligands and water. The results of the analysis were shown as binding affinity.

### 2.7 Molecular dynamics simulation analysis

In order to investigate the potential working mechanisms of the synthesized compound, MD simulations were carried out for compound 5i that demonstrated a theoretical docking score of −8.9 kcal/mol. The docking complex that involves ERα and the ligand was studied through MD simulations for its stability (Alajmi et al., 2018). The MD simulation was performed in the ligand–protein complex with the lowest MM-GBSA binding free energy value. The MD simulation was executed for 200 ns using Desmond software (Zamzami, 2023). First, a cubic simulation box was selected for the system builder panel of the Desmond–Schrodinger interface. Then, the TIP3P explicit water model was built. The distance between the simulation box and the protein surface remained equal to 10 Å. Additionally, to neutralize the system and to make the electrolyte environment isotonic with the NaCl, we added 150 mM (mM) of NaCl. Approximately 2,000 iterations were required for the system to achieve its minimum configuration. The minimized system was then subjected to a 200-ns MD simulation using the default relaxation option, at 300 K and 1 Bar under the NpT ensemble, Trevor 272. The Nose–Hoover chain (Brańka, 2000) thermostat and the Martyna–Tobias–Klein barostat (Martyna et al., 1994) were used to set the temperature and pressure at the desired levels. Energy and structural information were collected every 10 ps and stored in trajectory files. The MD simulation was performed in fs using a 2-fs time step. Maestro software was used to analyze the trajectories and three-dimensional structure.

## 3 Results and discussion

**FIGURE 2:**
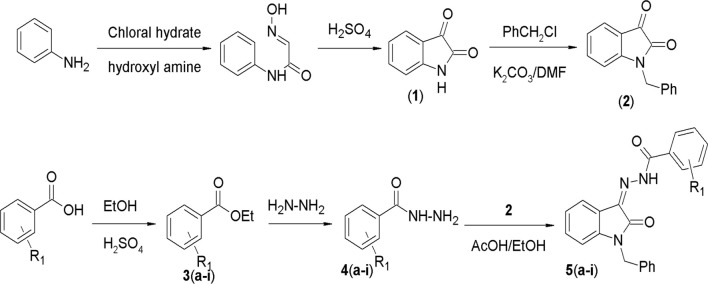
General scheme for the synthesis of compounds.

In this study, a series of N'-(1-benzyl-2-oxo-1, 2-dihydro-3H-indol-3-ylidene) hydrazide derivatives (5a–i) was synthesized, spectroscopically analyzed, and then subjected to the study of anticancer effects in BC cell line MCF-7. The synthesis involved several steps, as outlined (given as Figure 2). The synthesis started with the reaction of aniline with chloral hydrate and hydroxylamine to yield α-isonitrosoacetanilide, which was then cyclized using a strong acid. Isatin thus prepared was benzylated using benzyl chloride in the presence of potassium carbonate in DMF. In parallel, a range of substituted and unsubstituted aromatic carboxylic acid hydrazides were prepared through esterification of the respective carboxylic acid and subsequent reaction with hydrazine hydrate. In the last step, these hydrazides were condensed with benzylisatin to yield a novel series of N-substituted isatin compounds. All synthesized compounds exhibited solubility in chloroform (CHCl_3_) at room temperature and displayed a singular chromatographic spot in various solvent systems on TLC, indicative of their homogeneity as individual compounds.

The structures of 5(a–i) were characterized based on FTIR spectroscopy, ^1^H NMR, ^13^C NMR, and elemental analysis (provided as Supplementary Material). The FTIR spectra showed a major peak attributed to the carbonyl (C=O) group in each of the compounds within the range 1,720 cm^−1^– 1,733 cm^−1^. Furthermore, in each of the compounds under consideration, a particular spectral feature stands out as a peak within the wavenumber range 3,189 cm^-1^–3,195 cm^-1^ that is assigned to the vibrational mode connected to the N-H group. In addition, a big peak related to the azomethine (C=N) functionality was visible in each compound between 1,663 cm^-1^ and 1,666 cm^-1^. C–O bond stretching was also observed in all the synthesized derivatives.

In NMR spectroscopy, the appearance of a broad singlet signal within the range 13–14 ppm, corresponding to the NH protons of the hydrazide moiety, substantiated the formation of the condensation product. The methylenic protons that directly bonded to the nitrogen of isatin displayed deshielded resonances as a singlet between 4.53 and 4.73 ppm. The aromatic protons of the aryl rings exhibited upfield shifts between 6 and 9 ppm, with multiplicity consistent with their substitution patterns. Further confirmation of the structural attributes was derived from ^13^C NMR spectroscopy. Notably, the characteristic carbonyl signal of isatin vanished at 183 ppm, and a new signal assigned to the C=N group appeared within the range 135–140 ppm. The persistence of other carbon signals in their respective chemical shift regions provided clear evidence for the formation of the condensation products **5(a–i)**.

### 3.1 Anticancer activity

The *in vitro* anticancer potential of the synthesized compounds (**5a–i**) was assessed against the MCF-7 cell line using the SRB proliferation assay. All the tested compounds showed notable cytotoxicity against the MCF-7 cell line, as indicated by their IC_50_ values presented in Table 1. Among them, N'-(1-benzyl-2-oxo-1,2-dihydro-3H-indol-3-ylidene)benzohydrazide (**5i**) exhibited the highest potency, with an IC_50_ value of 9.29 ± 0.97 µM. Compounds N'-(1-benzyl-2-oxo-1,2-dihydro-3H-indol-3-ylidene)-2-hydroxybenzohydrazide **(5d)**, N'-(1-benzyl-2-oxo-1,2-dihydro-3H-indol-3-ylidene)pyridine-2-carbohydrazide **(5f)**, and N'-(1-benzyl-2-oxo-1,2-dihydro-3H-indol-3-ylidene)-3,4,5 trihydroxybenzohydrazide **(5g)** also exhibited inhibition at IC_50_ values of 13.34 ± 2.53, 12.35 ± 0.65, and 12.09 ± 4.08 µM, respectively.

**TABLE 1:** Cytotoxicity of test compounds against cancer cell line MCF-7.

Compound	Name	R	IC_50_ (µM ± SD)
5a	N'-(1-benzyl-2-oxo-1,2-dihydro-3H-indol-3-ylidene)pyridine-4-carbohydrazide	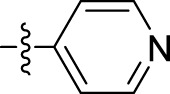	31.00 ± 11.50
5b	N'-(1-benzyl-2-oxo-1,2-dihydro-3H-indol-3-ylidene)-3-bromobenzohydrazide	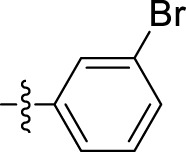	48.90 ± 24.6
5c	N'-(1-benzyl-2-oxo-1,2-dihydro-3H-indol-3-ylidene)-3-iodobenzohydrazide	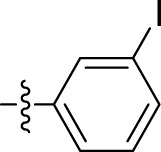	33.13 ± 9.57
5d	N'-(1-benzyl-2-oxo-1,2-dihydro-3H-indol-3-ylidene)-2-hydroxybenzohydrazide	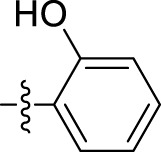	13.34 ± 2.53
5e	N'-(1-benzyl-2-oxo-1,2-dihydro-3H-indol-3-ylidene)-4-fluorobenzohydrazide	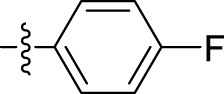	39.47 ± 12.58
5f	N'-(1-benzyl-2-oxo-1,2-dihydro-3H-indol-3-ylidene)pyridine-2-carbohydrazide	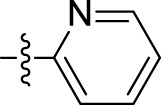	12.35 ± 0.65
5g	N'-(1-benzyl-2-oxo-1,2-dihydro-3H-indol-3-ylidene)-3,4,5trihydroxybenzohydrazide	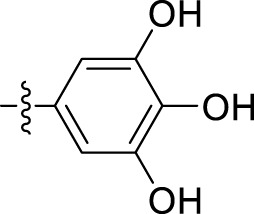	12.09 ± 4.08
5h	N'-(1-benzyl-2-oxo-1,2-dihydro-3H-indol-3-ylidene)-2-chlorobenzohydrazide	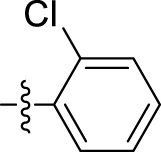	22.99 ± 2.75
5i	N'-(1-benzyl-2-oxo-1,2-dihydro-3H-indol-3-ylidene)benzohydrazide	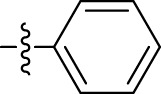	9.29 ± 0.97

**FIGURE 3:**
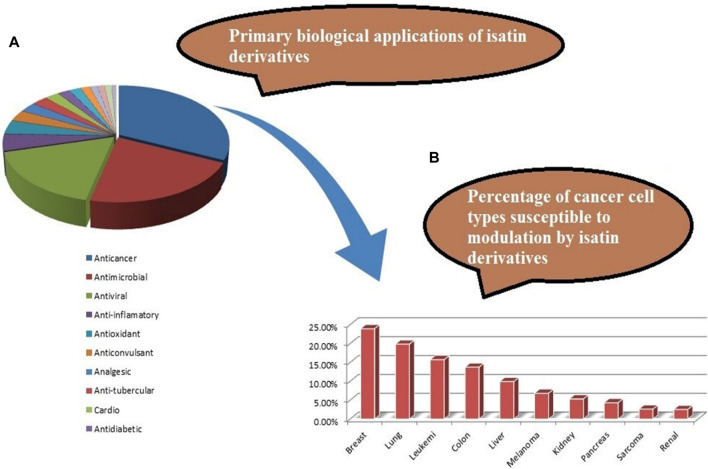
**(A)** Reported pharmacological actions of isatin derivatives. **(B)** Percentage of different types of target cancers (adapted from the study by Ferraz de Paiva et al., 2021).

Various isatin derivatives with different substitutions have been investigated and reported for the inhibition of the growth of BC cells to varying degrees (Figure 3). Several isatin derivatives, including semaxanib, sunitinib, nintedanib, and hesperadin, are in clinical use and have been reviewed recently (Kumar et al., 2018; Ding et al., 2020). In our study, the investigation into the structure–activity relationship (SAR) has revealed that the arrangement of substituent groups on the aryl ring and the placement of the heteroatom within the heteroaryl ring exert an influence on the cytotoxic potential of these compounds against MCF-7 cell lines.

The most potent compound, **5i**, characterized by an unsubstituted phenyl ring, showed an IC_50_ value of 9.29 ± 0.97 µM (Table 1). The introduction of halogen substituent groups onto the phenyl ring (5c, 5e, and 5 h) resulted in decreased activity compared to the unsubstituted compound **5i**. Remarkably, among the derivatives with phenyl substitutions, compounds with substituents at the 2-position of the phenyl ring displayed the highest activity level. For instance, compound **5d**, featuring an electron-donating hydroxyl group at the 2-position of the phenyl ring, demonstrated a pronounced IC_50_ value of 13.34 ± 2.53 µM. Similarly, compound **5h**, bearing a 2-chloro substitution, displayed a slightly reduced IC_50_ value of 22.99 ± 2.75 µM.

However, compounds containing the substitution at the 3-position of the phenyl ring were less active than their 2-substituted counterparts. For example, compounds such as **5b** and **5c**, with iodo and bromo groups of the phenyl ring as electron-withdrawing groups, showed decreased activity with IC_50_ values of 48.90 ± 24.6 µM and 33.13 ± 9.57 µM, respectively. Substitutions at the 4-position of the phenyl ring on **5e** yielded reduced reactivity, with an IC_50_ value of 39.47 ± 12.58 µM. In addition, a compound containing several substitutions on the phenyl ring, for example, compound **5g** with 3,4,5-trihydroxy configuration, had an IC_50_ value of 12.09 ± 4.08 µM, exceeding that of the 2-substituted, 3-substituted, and 4-substituted derivatives.

These findings collectively underscore the crucial role played by the inherent characteristics and strategic arrangement of substituent groups on the phenyl ring in determining the cytotoxic activity of these compounds.

Furthermore, compounds having a heteroaryl pyridyl ring instead of the phenyl ring are observed to be highly dependent on the spatial orientation of the heteroatom in this regulation. For instance, compound **5f**, garnished with a 2-pyridyl ring moiety, shows better activity, with an IC_50_ value of 12.35 ± 0.65 µM, while compound 5a, with 4-pyridyl ring substitution, showed a lower toxic effect (IC_50_ value: 31.00 ± 11.50 µM).

This discernible activity pattern conforms to the deductions on the location of the heteroatom made in the case of substitution on the phenyl ring. In particular, the presence of the heteroatom at the 2-position, analogically to the substitution at the 2-position of the phenyl ring, results in superior activity compared to the 4-position, comparable to the substitution at the 4-position of the phenyl ring, thereby highlighting the role of the heteroatom position in cytotoxicity.

### 3.2 Downregulated estrogen receptor levels in MCF-7 cells

**FIGURE 4:**
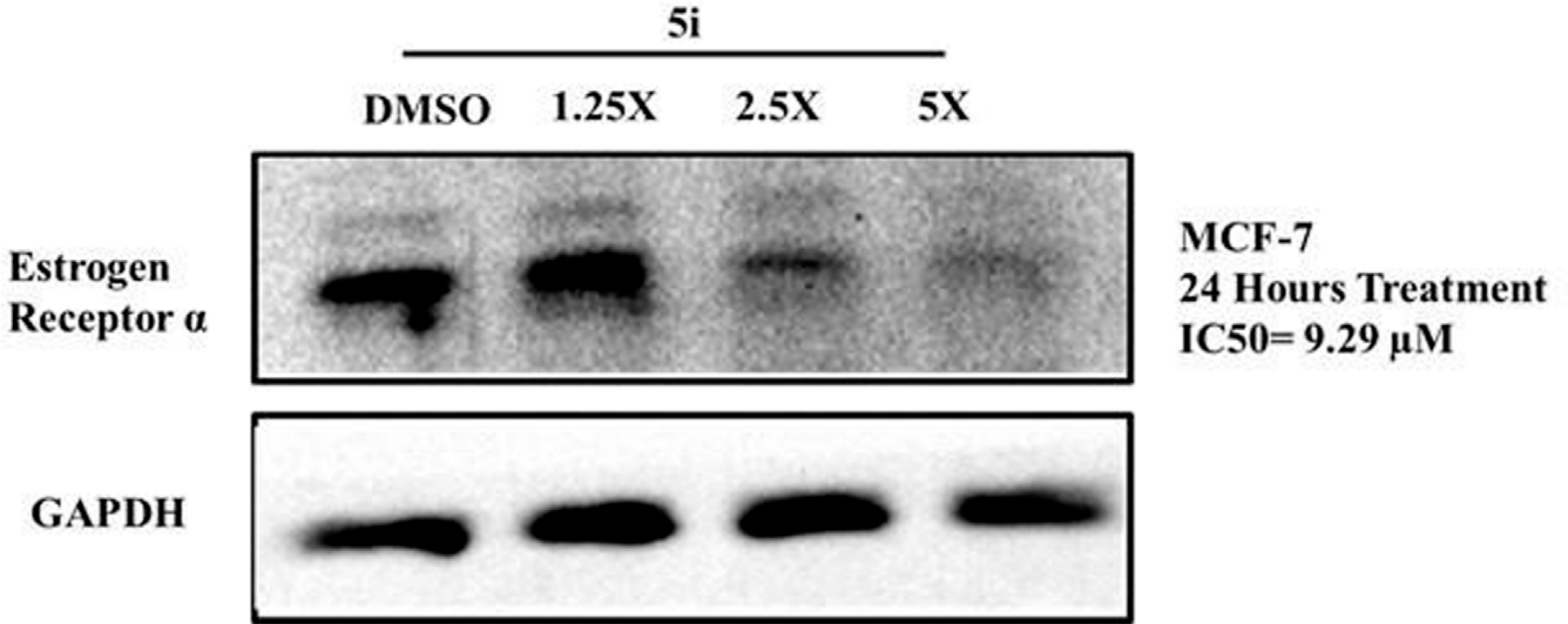
Compound 5i downregulates ERα levels in MCF-7 cells. Cells were treated with IC_50_ concentrations of 1.25X, 2.5X, and 5X of 5i for 24 h. Treatment with DMSO served as a solvent control. Cells were then lysed, and equal amounts of protein for all samples were analyzed for ERα levels through Western blotting using specific antibodies. GAPDH was used as a loading control.

To determine the mechanism of action for the antiproliferative activity of 5i, we determined its effect on the expression levels of ERα in ER+ MCF-7 cells. Cells were treated with IC_50_ concentrations of 1.25X, 2.5X, and 5X of **5i** for 24 h, and the expression of ERα was analyzed through Western blotting. Treatment with concentrations of 2.5X and 5X of **5i** robustly reduced ERα expression compared to DMSO-treated solvent control cells (Figure 4). The loading control GAPDH remained unchanged at all the concentrations, indicating equal loading of whole-cell lysate in all the wells.

In a recent review on the pharmacological profile of isatin derivatives, Ferraz de Paive et al. (2020) claimed that among different biological activities, the anticancer effect was more pronounced (Ferraz de Paiva et al., 2021). The main cancer type where isatin derivatives were tested was BC. It is well established that more than 70% cases of BC are estrogen-mediated (Fujiki et al., 2014). Our results strongly support that a major contributing mechanism underlying the cytotoxic effect of isatin derivatives in estrogen-responsive cancer cells is the degradation of ERα. However, no previous report on the antagonistic/inhibitory action of any isatin derivative on ERs, ERα or ERβ, in estrogen-responsive BC or in any other system (*in vitro* or *in vivo*) has been published. So, these results strongly suggest that this derivative with isatin moiety has the potential to be further developed into a SERD to be used alone or in combination with other anti-angiogenesis agents, checkpoint, or kinase inhibitors.

### 3.3 Chemical space, drug-likeness prediction, and pharmacokinetics profiling of compounds 5a–5i

Table 2 provides a general view of the main physicochemical properties measured using DataWarrior software. These features take care of numerous traits important to the assessment of the synthetic compounds as drug candidates. The multifaceted analysis of these properties points out some very prominent features that qualify the synthesized compounds as promising drug candidates. First, the molecular weight and monoisotopic mass offer significant information about the size and mass distribution of compounds that elucidate the chemically important properties affecting the drug absorption, distribution, metabolism, and excretion (ADME). Furthermore, properties such as lipophilicity and solubility in the water, represented by cLogP and cLogS, respectively, are key determinants of how the compounds will behave pharmacokinetically. Compounds with ideal lipophilicity and solubility often exhibit much better ADME properties, contributing to the improved bioavailability and efficacy. Moreover, the existence of functional groups like H acceptors and H donors governs the ability of the molecule to bind to the target biomolecules through hydrogen bonding, which is one of the key aspects of the molecular recognition and binding affinity of molecule. This analysis includes the molecular surface area and polarity descriptors that reflect the capability of the compound to permeate the biological membranes and to reach intracellular targets, which in turn engender its pharmacodynamic profile. In addition, parameters like drug-like propensity, irritant potential, and molecular complexity play many important roles in the evaluation of the drug-like quality, safety properties, and synthetic accessibility of the compound, which are very crucially relevant to the drug development. The presence of important structural elements such as aromaticity, cycles, and stereochemistry additionally enhances its bioactivity and also therapeutic potential. In general, the complete assessment of these physicochemical characteristics suggests that the synthesized compounds have the potential to be very promising drugs; hence, further detailed studies on their pharmacological activities and therapeutic applications are necessary.

**TABLE 2:** Chemoinformatics analysis of synthesized compounds.

Column name	5a	5b	5c	5d	5e	5f	5 g	5 h	5i
Total mol. weight	356.384	434.292	481.288	371.395	373.386	356.384	403.393	389.841	355.396
Monoisotopic mass	356.127	433.0325	481.028	371.126	373.122	356.127	403.1168	389.093	355.132
cLogP	2.602	4.3284	4.403	3.257	3.704	2.6563	2.5661	4.2092	3.6032
cLogS	−4.475	−6.102	−6.284	−4.972	−5.582	−4.497	−4.38	−6.004	−5.268
H acceptors	6	5	5	6	5	6	8	5	5
H donors	1	1	1	2	1	1	4	1	1
Total surface area	272.03	291.9	299.55	279.62	279.62	272.03	292.32	288.69	273.27
Relative PSA	9.2336	0.1802	0.1756	0.23496	0.188	9.2336	0.31348	0.1822	0.19248
Polar surface area	74.66	61.77	61.77	82	61.77	74.66	122.46	61.77	61.77
Drug-likeness	6.5269	4.7369	6.9034	6.4978	5.1869	6.5269	6.4978	6.5395	6.5269
Irritant	None	None	None	None	None	None	None	None	None
Nasty functions	Acyl hydrazone	Acyl hydrazones	Acyl hydrazones	Acyl hydrazones	Acyl hydrazones	Acyl hydrazone	Acyl hydrazones	Acyl hydrazones	Acyl hydrazones
Shape index	0.55556	0.53571	0.53571	0.53571	0.57143	0.55556	0.53333	0.53571	0.55556
Molecular flexibility	0.31329	0.31467	0.31467	0.31467	0.31467	0.31329	0.317	0.3183	0.31329
Molecular complexity	0.82315	0.83059	0.83059	0.83059	0.82582	0.82315	0.84087	0.83554	0.82132
Fragments	1	1	1	1	1	1	1	1	1
Non-hydrogen atoms	27	28	28	28	28	27	30	28	27
Non-C/H atoms	6	6	6	6	6	6	8	6	5
Metal atoms	0	0	0	0	0	0	0	0	0
Electronegative atom	6	6	6	6	6	6	8	6	5
Stereo lefts	0	0	0	0	0	0	0	0	0
Rotatable bonds	4	4	4	4	4	4	4	4	4
Ring closures	4	4	4	4	4	4	4	4	4
Aromatic atoms	18	18	18	18	18	18	18	18	18
Sp3 carbon fraction	0.047619	0.045455	0.045455	0.045455	0.045455	0.047619	0.045455	0.045455	0.045455
Sp3 atoms	1	1	1	2	1	1	4	1	1
Symmetric atom	4	2	2	2	4	4	5	2	4
Small rings	4	4	4	4	4	4	4	4	4
Carbo rings	2	3	3	3	3	2	3	3	3
Hetero rings	2	1	1	1	1	2	1	1	1
Saturated rings	0	0	0	0	0	0	0	0	0
Non-aromatic ring	1	1	1	1	1	1	1	1	1
Aromatic rings	3	3	3	3	3	3	3	3	3
Saturated carbon rings	0	0	0	0	0	0	0	0	0
Non-aromatic carbon ring	0	0	0	0	0	0	0	0	0
Carbon aromatic ring	2	3	3	3	3	2	3	3	3
Saturated hetero ring	0	0	0	0	0	0	0	0	0
Non-aromatic hetero ring	1	1	1	1	1	1	1	1	1
Hetero aromatic ring	1	0	0	0	0	1	0	0	0
Amide	1	1	1	1	1	1	1	1	1
Amines	0	0	0	0	0	0	0	0	0
Alkyl amines	0	0	0	0	0	0	0	0	0
Aromatic amines	0	0	0	0	0	0	0	0	0
Aromatic nitrogen	1	0	0	0	0	1	0	0	0
Basic nitrogen	0	0	0	0	0	0	0	0	0
Acidic oxygen	0	0	0	0	0	0	0	0	0
Globularity SVD	0.33914	0.3235	0.32945	0.34663	0.33395	0.33914	0.33756	0.34848	0.33564
Globularity vol	0.69798	0.6829	0.68036	0.6924	0.69143	0.69798	0.67078	0.69103	0.69396
VDW surface	340.61	364.17	371.16	352.06	349.99	340.61	384.53	359.13	345.27
VDW volume	344.58	368.75	377.24	357.78	353.88	344.58	389.41	367.49	348.66
Lipinski rule validation	Yes	Yes	Yes	Yes	Yes	Yes	Yes	Yes	Yes

Recognition and minimization of illegitimate actions of pharmacological entities are an issue that programs aiming at the procurement of druggable molecule candidates need to deal with. The initial characterization of PK behavior represents a critical tool to address safety issues likely to be associated with hit compounds. We used *in silico* pharmacokinetics prediction tools, particularly the pkCSM tool, for the assessment of the activity profiles of the compounds 5a–5i in this study. The initial evaluation of compounds 5a–5i through pkCSM prediction showed very promising activity profiles (Table 3). Importantly, all the compounds 5a–5i fell within the safe limits for the ADME parameters. The results imply that the synthetic substances have good PK characteristics, a key factor in drug discovery and development. Despite the predictions of ADME profiles, in the case of multiple models, more effort should be taken to enrich the literature with consensus ADME profiling and experimental data relevant to the pharmacokinetics of the synthesized compounds.

**TABLE 3:** Pharmacokinetics profile of synthesized compounds (5a–5i).

Property	Model name	5a	5b	5c	5d	5e	5f	5 g	5 h	5i	Unit
Absorption	Water solubility	−3.989	−5.661	−5.647	−4.434	−5.453	−3.872	−3.623	−5.585	−5.133	Numeric (log mol/L)
Absorption	CaCO_2_ permeability	1.235	1.315	1.313	1.034	1.319	1.037	0.118	1.317	1.321	Numeric (log Papp in 10–6 cm/s)
Absorption	Intestinal absorption (human)	96.501	92.916	93.552	93.482	93.894	96.442	73.742	92.983	94.644	Numeric (% absorbed)
Absorption	Skin permeability	−2.742	−2.729	−2.731	−2.822	−2.75	−2.707	−2.736	−2.729	−2.732	Numeric (log Kp)
Absorption	P-glycoprotein substrate	No	Yes	Yes	Yes	Yes	No	Yes	Yes	Yes	Categorical (yes/no)
Absorption	P-glycoprotein I inhibitor	Yes	Yes	Yes	Yes	Yes	Yes	Yes	Yes	Yes	Categorical (yes/no)
Absorption	P-glycoprotein II inhibitor	Yes	Yes	Yes	Yes	Yes	Yes	Yes	Yes	Yes	Categorical (yes/no)
Distribution	VDss (human)	−0.083	−0.175	−0.165	−0.332	−0.361	−0.132	−0.146	−0.192	−0.208	Numeric (log L/kg)
Distribution	Fraction unbound (human)	0	0	0	0	0	0	0	0	0	Numeric (Fu)
Distribution	BBB permeability	−0.291	−0.078	−0.083	0.132	−0.044	−0.309	−1.013	−0.077	−0.076	Numeric (log BB)
Distribution	CNS permeability	−2.296	−1.788	−1.821	−2.104	−1.958	−2.303	−2.508	−1.81	−1.925	Numeric (log PS)
Metabolism	CYP2D6 substrate	No	No	No	No	No	No	No	No	No	Categorical (yes/no)
Metabolism	CYP3A4 substrate	Yes	Yes	Yes	Yes	Yes	Yes	Yes	Yes	Yes	Categorical (yes/no)
Metabolism	CYP1A2 inhibitor	Yes	No	No	Yes	Yes	Yes	No	No	Yes	Categorical (yes/no)
Metabolism	CYP2C19 inhibitor	Yes	Yes	Yes	Yes	Yes	Yes	No	Yes	Yes	Categorical (yes/no)
Metabolism	CYP2C9 inhibitor	Yes	Yes	Yes	Yes	Yes	Yes	Yes	Yes	Yes	Categorical (yes/no)
Metabolism	CYP2D6 inhibitor	No	No	No	No	No	No	No	No	No	Categorical (yes/no)
Metabolism	CYP3A4 inhibitor	Yes	Yes	Yes	Yes	Yes	Yes	No	Yes	Yes	Categorical (yes/no)
Excretion	Total clearance	0.468	−0.121	−0.411	0.208	0.151	0.345	−0.126	0.106	0.424	Numeric (log mL/min/kg)
Excretion	Renal OCT2 substrate	Yes	No	No	No	No	Yes	No	No	No	Categorical (yes/no)
Toxicity	Max. tolerated dose (human)	0.051	0.221	0.208	−0.417	0.232	−0.05	0.001	0.218	0.203	Numeric (log mg/kg/day)

### 3.4 Toxicity prediction using Toxtree

The toxicity prediction by Toxtree, a widely deployed *in silico* toxicological approach, indicates good toxicity/safety profiles of tested compounds. Traditionally, such results are obtained only through the use of a large number of model animals. Additionally, animal experimentation is a major obstacle in terms of cost and time consumed (Rim, 2020). An intensive *in silico* computational examination sheds light on the molecular intricacy of each compound. The investigated series of the compounds has shown a one-way tendency towards the non-toxic traits across various tests performed, as shown in Table 4.

**TABLE 4:** Predicted toxicity parameters of synthesized compounds 5a–i.

Compound	Toxicity						Metabolism
	Cramer’s rule	*In vitro* mutagenicity and carcinogenicity	Skin irritation/corrosion	Eye irritation/corrosion	Alert for DNA binding	Structure alerts for the *in vivo* micronucleus assay in rodents	Cytochrome P450-mediated drug metabolism
5a	Negative	Negative	Negative	Negative	Negative	Negative	Positive
5b	Negative	Negative	Negative	Negative	Negative	Negative	Positive
5c	Negative	Negative	Negative	Negative	Negative	Negative	Positive
5d	Negative	Negative	Negative	Negative	Negative	Negative	Positive
5e	Negative	Negative	Negative	Negative	Negative	Negative	Positive
5f	Negative	Negative	Negative	Negative	Negative	Negative	Positive
5g	Negative	Negative	Negative	Negative	Negative	Negative	Positive
5h	Negative	Negative	Negative	Negative	Negative	Negative	Positive
5i	Negative	Negative	Negative	Negative	Negative	Negative	Positive

The components showed a negative potential for ocular irritation and the nullification of skin sensitization alerts, thus supporting their safety for dermal use. Moreover, the compounds showed no genotoxic carcinogenicity, thus implying their compatibility with the biological system at the genetic level (non-DNA binding). The compounds were also found harmless in other assessments, such as non-genotoxic carcinogenicity.

### 3.5 Molecular docking

**FIGURE 5:**
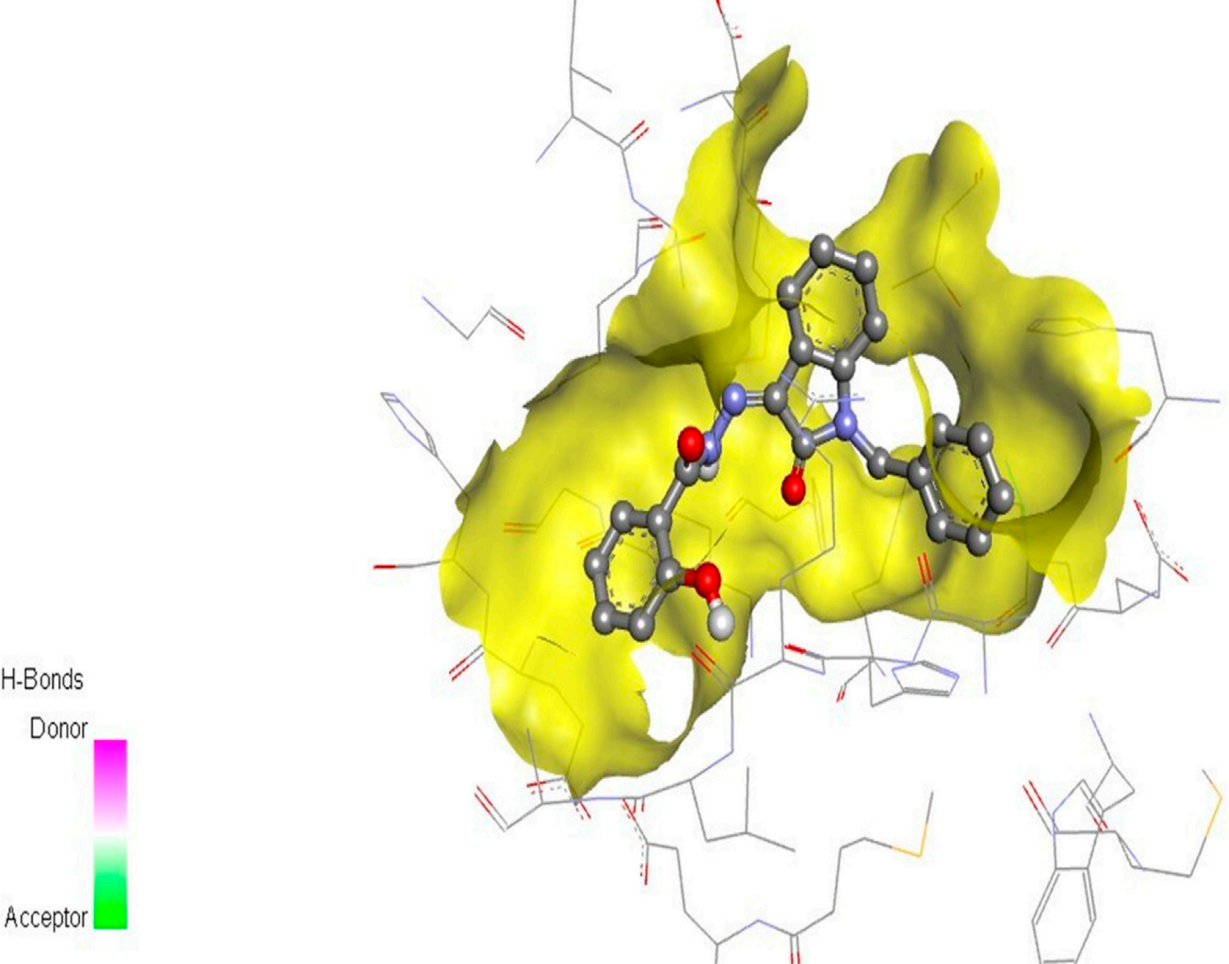
Compound 5d docked with ERα (PDB ID: 3ERT) 3D pose.

**FIGURE 6:**
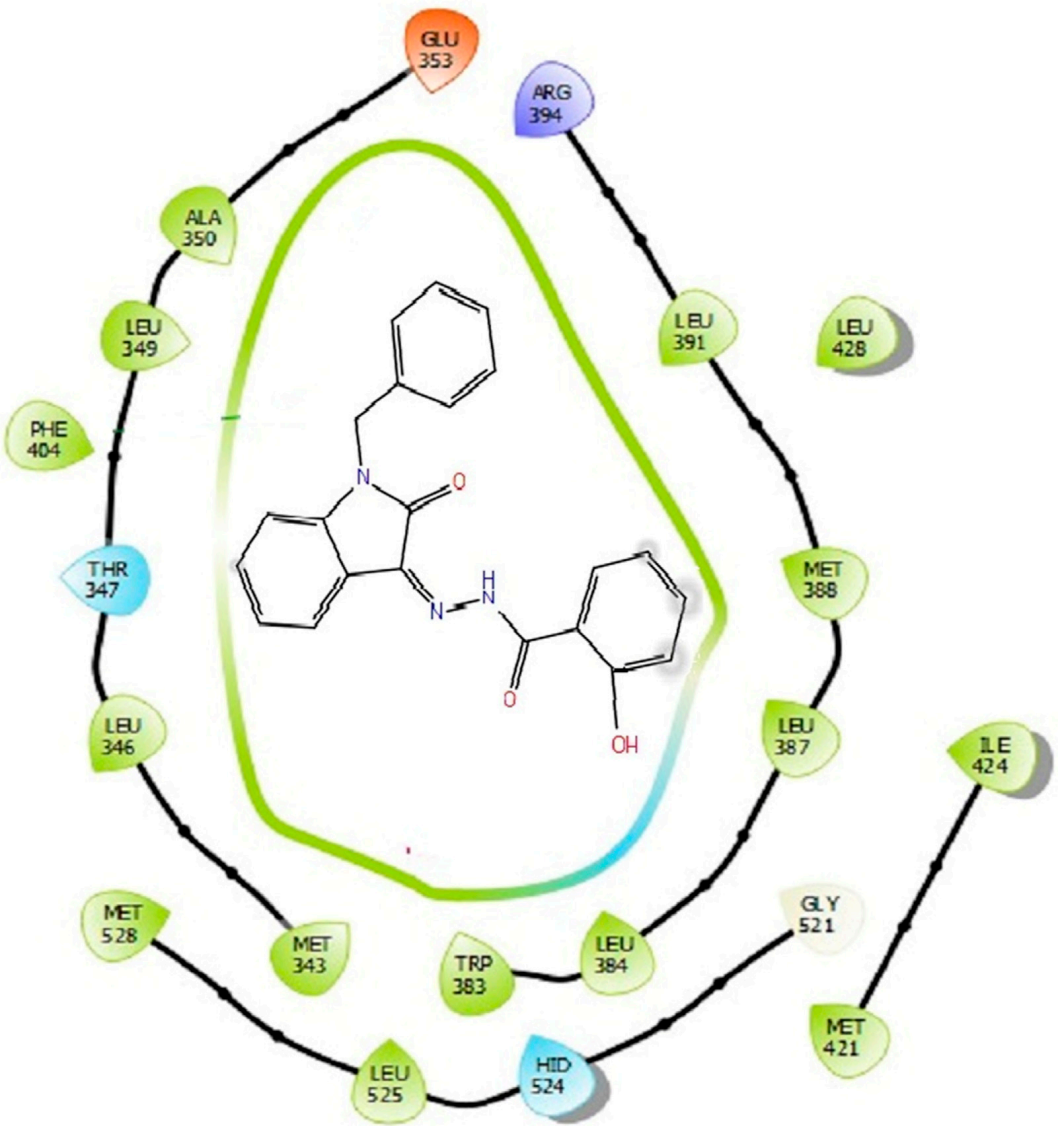
Binding interactions of compound 5d with ERα (PDB ID: 3ERT) 2D pose.

**FIGURE 7:**
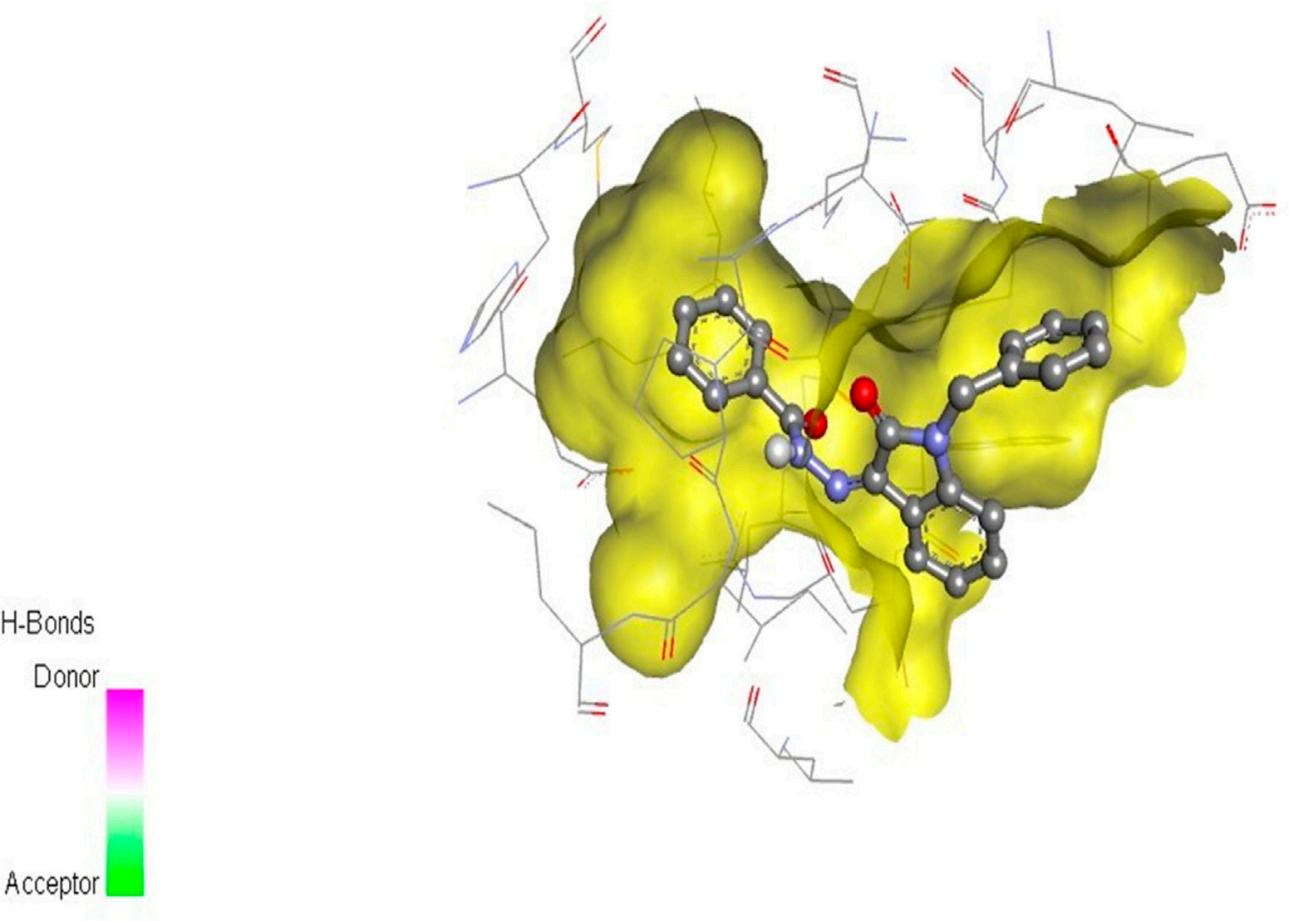
Compound 5i docked with ERα (PDB ID: 3ERT) 3D pose.

**FIGURE 8:**
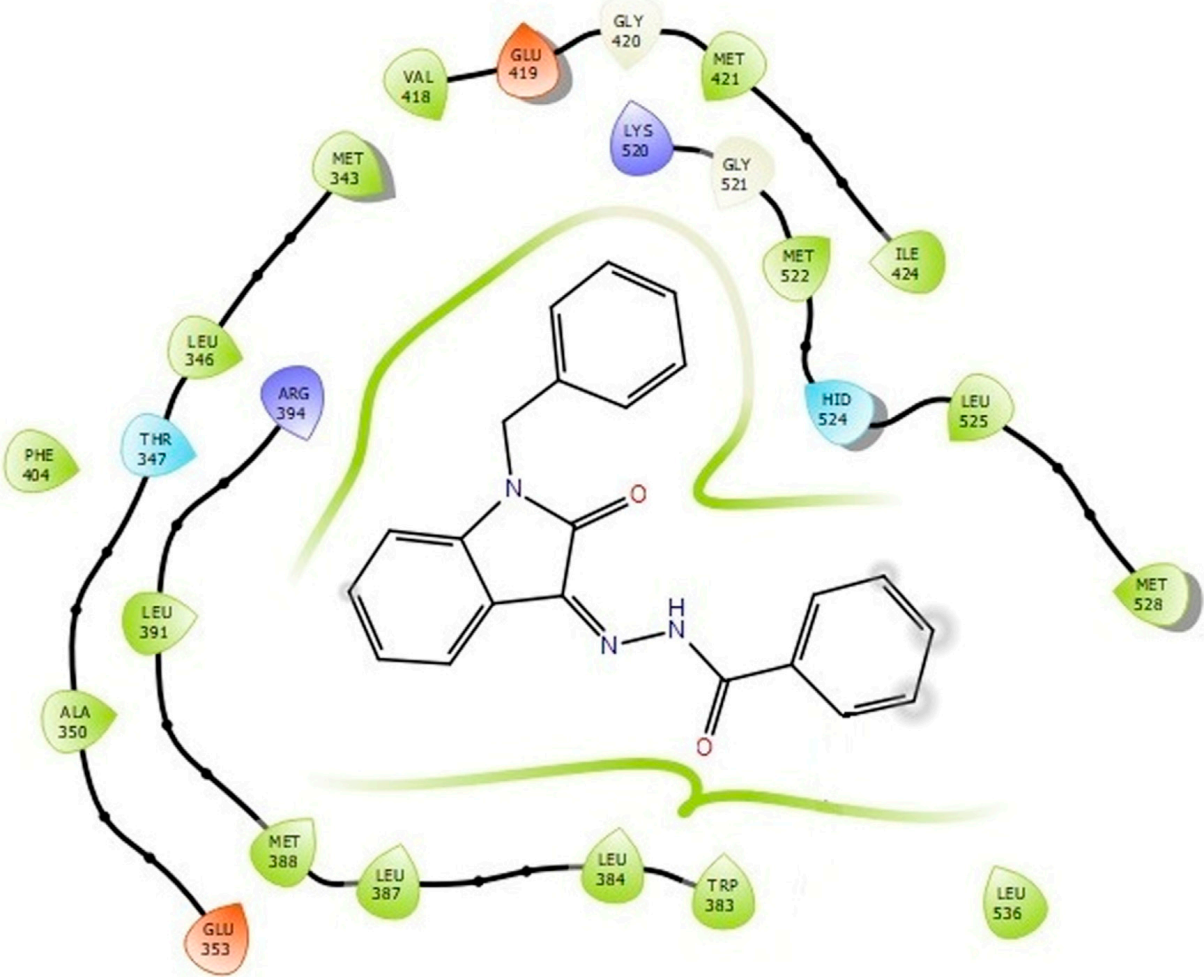
Binding interactions of compound 5i with ERα (PDB ID: 3ERT) 2D pose.

The docking studies were undertaken to predict the binding affinities of the ligands (compounds) with the target protein (ERα), as shown in Table 5. The docking scores were calculated in terms of binding affinities. These findings substantiate the prominence of hydrophobic interactions, specifically pi–sigma, pi–pi, pi–anion, and pi–alkyl interactions, involving the indolinone and aromatic ring constituents of the isatin–hydrazide conjugates and the binding residues within the target proteins. This assertion is reinforced by the graphical representations shown in Figures 5–8. The origin of these hydrophobic interactions can be attributed to the optimal positioning of the indolinone ring within a hydrophobic pocket.

**TABLE 5:** Docking scores of ligands with estrogen receptor α.

Compound	Binding affinity (kcal/mol)	Amino acid residue
5a	−8.3	VAL418, ARG394, LEU391, MET388, LEU387, LEU384, and TRP383
5b	−8.2	TRP383, ARG394, LEU391, MET388, LEU387, and LEU384
5c	−8.3	GLU419, ARG394, LEU391, MET388, LEU387, LEU384, and TRP383
5d	−9.2	ARG394, LEU391, MET388, LEU387, LEU384, TRP383, GLU353, ALA350, LEU349, PHE404, THR347, LEU346, and MET353
5e	−8.8	MET443, ARG394, LEU391, MET388, LEU387, LEU384, and TRP383
5f	−8.5	ARG394, LEU391, MET388, LEU387, LEU384, and TRP383
5g	−8.8	ARG394, LEU391, MET388, LEU387, LEU384, and TRP383
5h	−8.2	ARG394, LEU391, MET388, LEU387, LEU384, and TRP383
5i	−8.9	ARG394, LEU391, MET388, LEU387, LEU384, TRP383, LYS 520, GLY521, MET522, HID524, LEU525, and MET528

Compounds 5i and 5d exhibited the best *in vitro* inhibition, which is further supported by the outcome of docking simulations. The binding affinity of compound 5i was attributed to van der Waals interactions with lysine (521), hydrogen bonding with histidine (524), as well as pi–alkyl and pi–sulphur interactions with methionine (522). Additionally, a pi–pi interaction was observed with leucine (387), while a pi–sigma bond was formed between the indolylidene ring and methionine (388). Furthermore, a pi–pi interaction was evident between the indolylidene ring and the hydrazide phenyl ring with tryptophan (383).

### 3.6 Molecular dynamics simulation analysis

**FIGURE 9:**
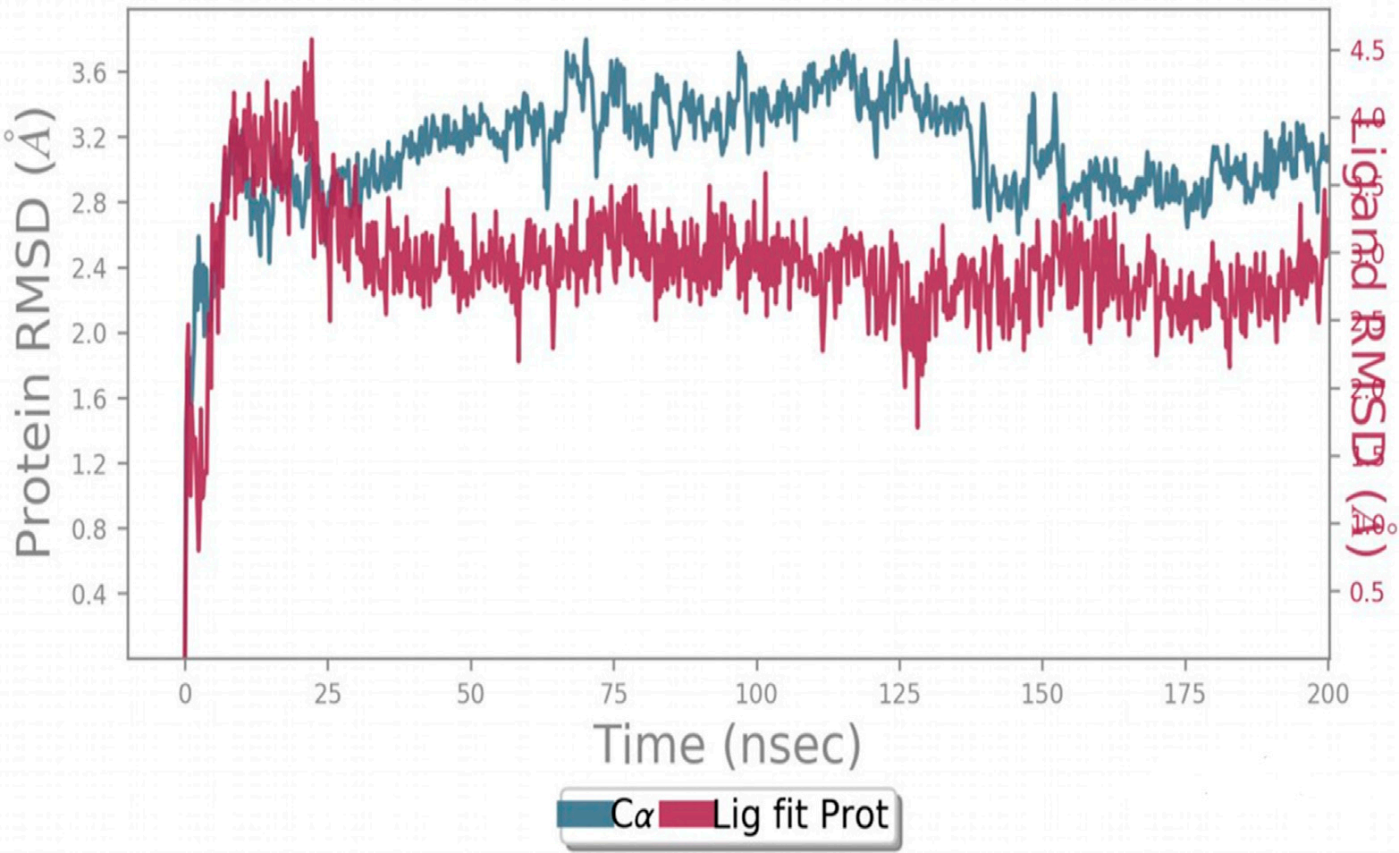
Protein–ligand RMSD.

MD simulation was used to analyze and evaluate the stability and dynamic behavior of the protein–ligand complex between ERα and its ligand. The docked ERα–ligand complex was simulated for 200 ns. As shown in Figure 9, RMSD exhibited comparable traces during the latter part of the initial half (75–100 ns) and the latter half (135–200 ns) of the simulation, implying that the whole system became effectively equilibrated. In contrast, during 10–25 ns, the movement of the complex completely repeated the trajectory of the Apo form (without ligand). Along the simulation, the RMSD values for the complex and Apo forms varied between 0 Å and 3.7 Å and 0 Å and 3.8 Å, respectively. Taken together, these results reflect a steady binding association between the ligand (compound 5i) and the ERα protein, facilitated by considerable interactions with key amino acids within the binding pocket.

**FIGURE 10:**
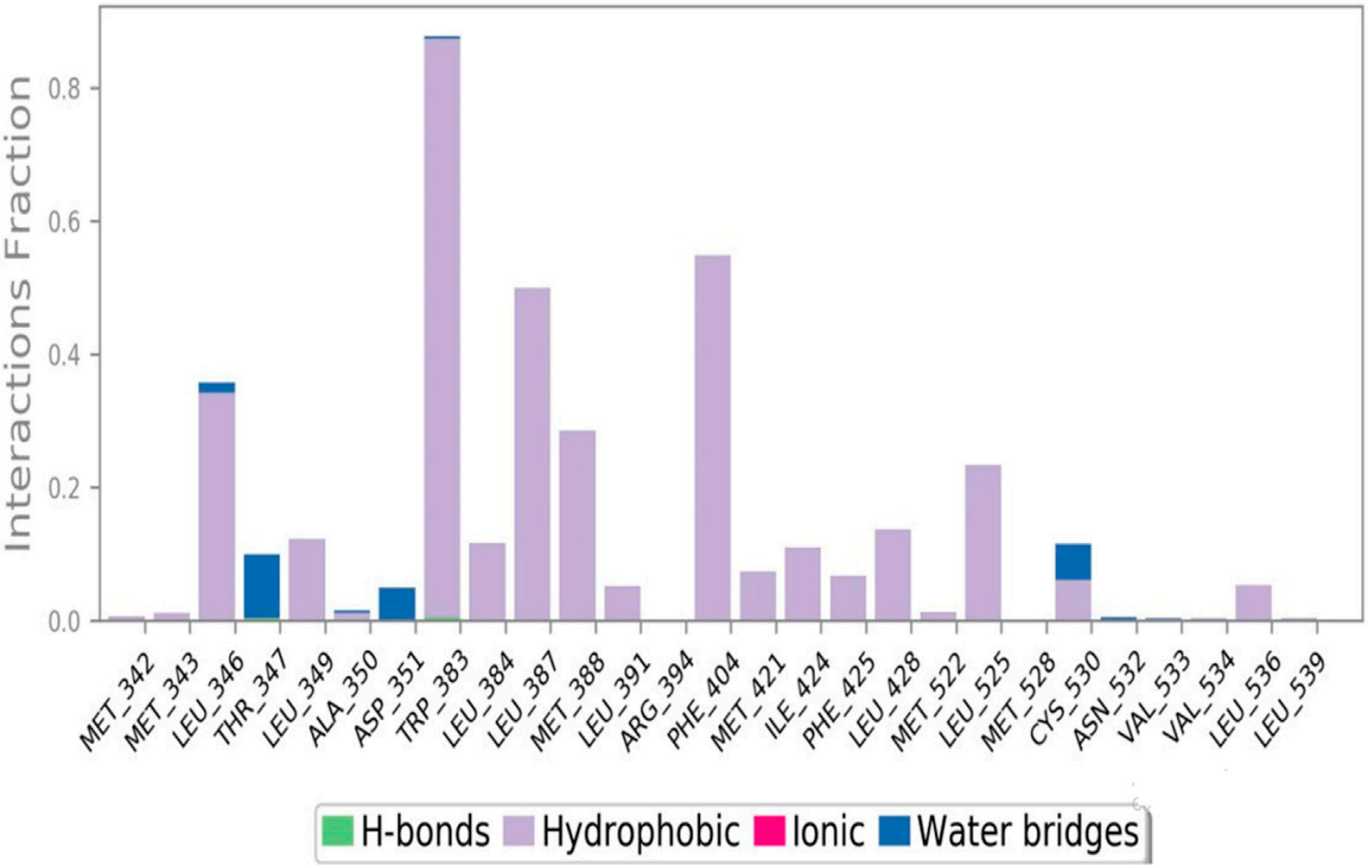
Protein–ligand contact stacked bar chart.

**FIGURE 11:**
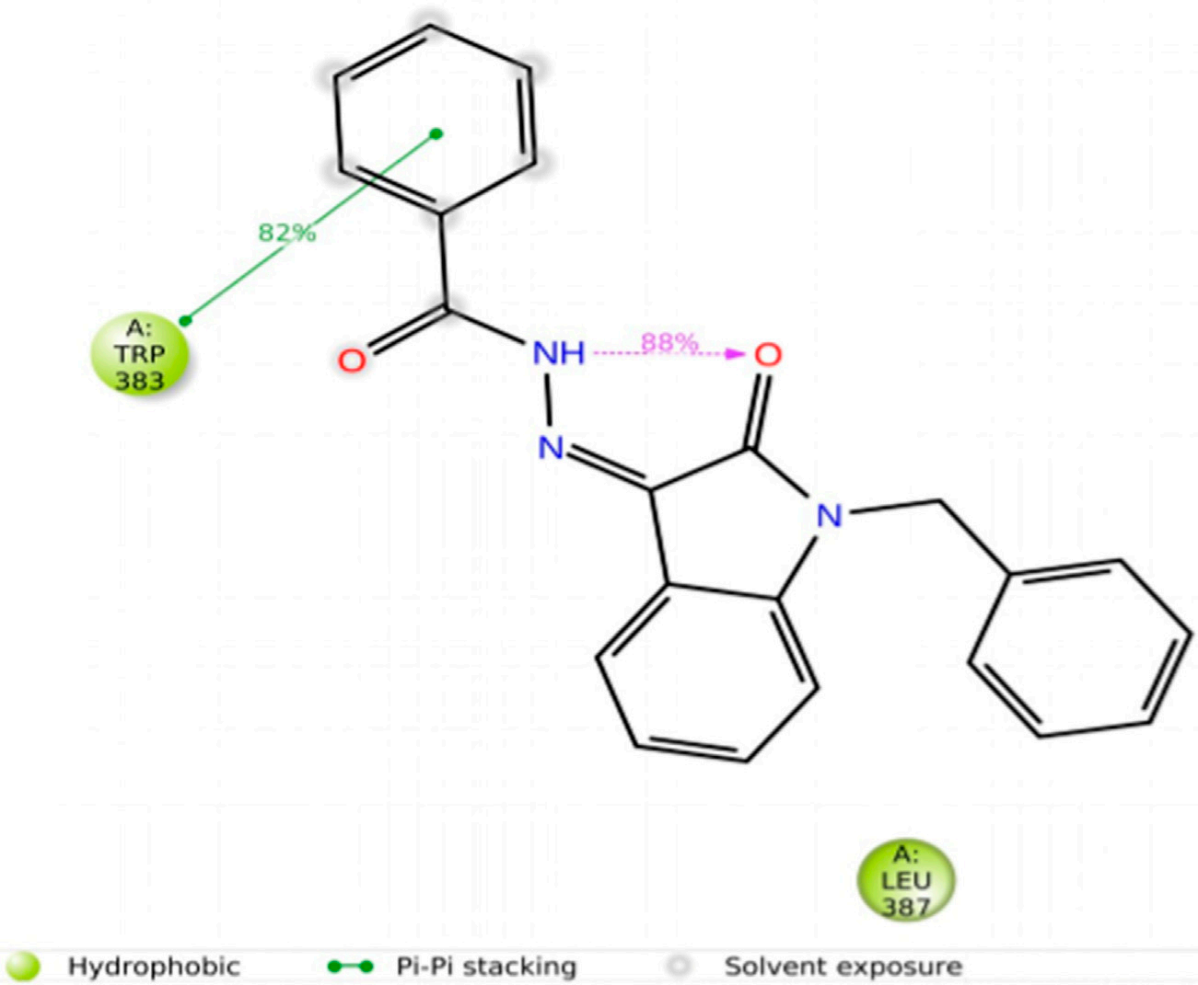
Protein–ligand contacts.

The interactions between the ligand and the protein were constantly surveyed during the whole of the simulation. These interactions were classified into four main categories, namely, hydrogen bonds, ionic interactions, water bridges, and hydrophobic interactions, schematically shown in Figures 10, 11. The normalized stacked bar chart (Figure 10) represents the fraction of time whenever particular interactions were sustained. For example, a value of 0.9 implies that the specific interactions were observed during 90% of the simulation period.

In accordance with the stacked bar charts, the ligand interaction fraction of TRP383 was approximately 0.9, and hydrogen bonds, electrostatic, and pi–alkyl interactions were the contributing types of interaction. In the beginning, there were hydrogen bonds with an interaction fraction of 0.01, then hydrophobic interactions from 0.01 to 0.88, and finally, there were water bridges for a short period of time. The mole fraction of the ligand with PHE404 was approximately 0.6, mainly including hydrophobic compensation. Analogously, the binding fraction with LEU387 was also high and consisted only of hydrophobic contacts. The interaction energy with LEU346 was approximately 0.38, which was mainly due to hydrophobic interactions and water bridges. A few other amino acids with minor interactions during short durations are presented in the stacked bar charts.

Importantly, hydrophobic interactions determine the binding of ligands. The fact that these hydrophobic interactions have a profound effect on the specificity of the drug, its metabolism, and its adsorption requires them to be taken into account when developing new drugs. Hydrophobic contacts can be categorized into three subtypes: π–cation, π–π, and other nonspecific interactions. Generally, it is a hydrophobic amino acid that interacts with an aromatic or aliphatic group on the ligand. This category has now been expanded to include π–cation interactions.

The current geometric criteria for hydrophobic interactions are defined as follows: π–cation, aromatic and charged groups less than 4.5 Å; π–π, two aromatic group stacked face to face or face to edge; and other, an uncharacterized hydrophobic side chain within 3.6 Å of the aromatic or aliphatic carbons of a ligand.

The criteria outlined herein constitute a foundation upon which to anchor mechanistic frameworks of hydrophobic interactions in the design and development of drugs (Sadiq et al., 2020).

**FIGURE 12:**
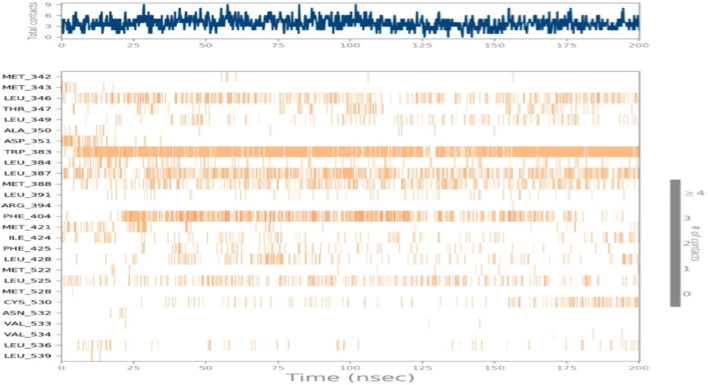
Total time simulation versus amino acid interactions.

The duration of the simulation is shown on the *x*-axis, and the interaction of each amino acid with the ligand is shown on the *y*-axis, as shown in Figure 12. The figure shows the interaction time of each amino acid involved across 200 ns of simulation. This analysis indicates that TRP383 showed one of the strongest interactions with the ligand. LEU387 displays a continuous interaction with negligible interruptions throughout the simulation, suggesting a stable interaction. However, the second important amino acid, PHE404, also displays a significant interaction with a ligand. Moreover, other amino acids that show strong and continuous interactions including LEU346, MET388, and LEU525 are also notable.

CYS530 initiates interaction in the second part of the simulation and maintains strong interaction afterward with only minor interruptions until the end of the simulation. In addition, LEU349 is significant and strong that has many minor interruptions throughout the simulation run.

**FIGURE 13:**
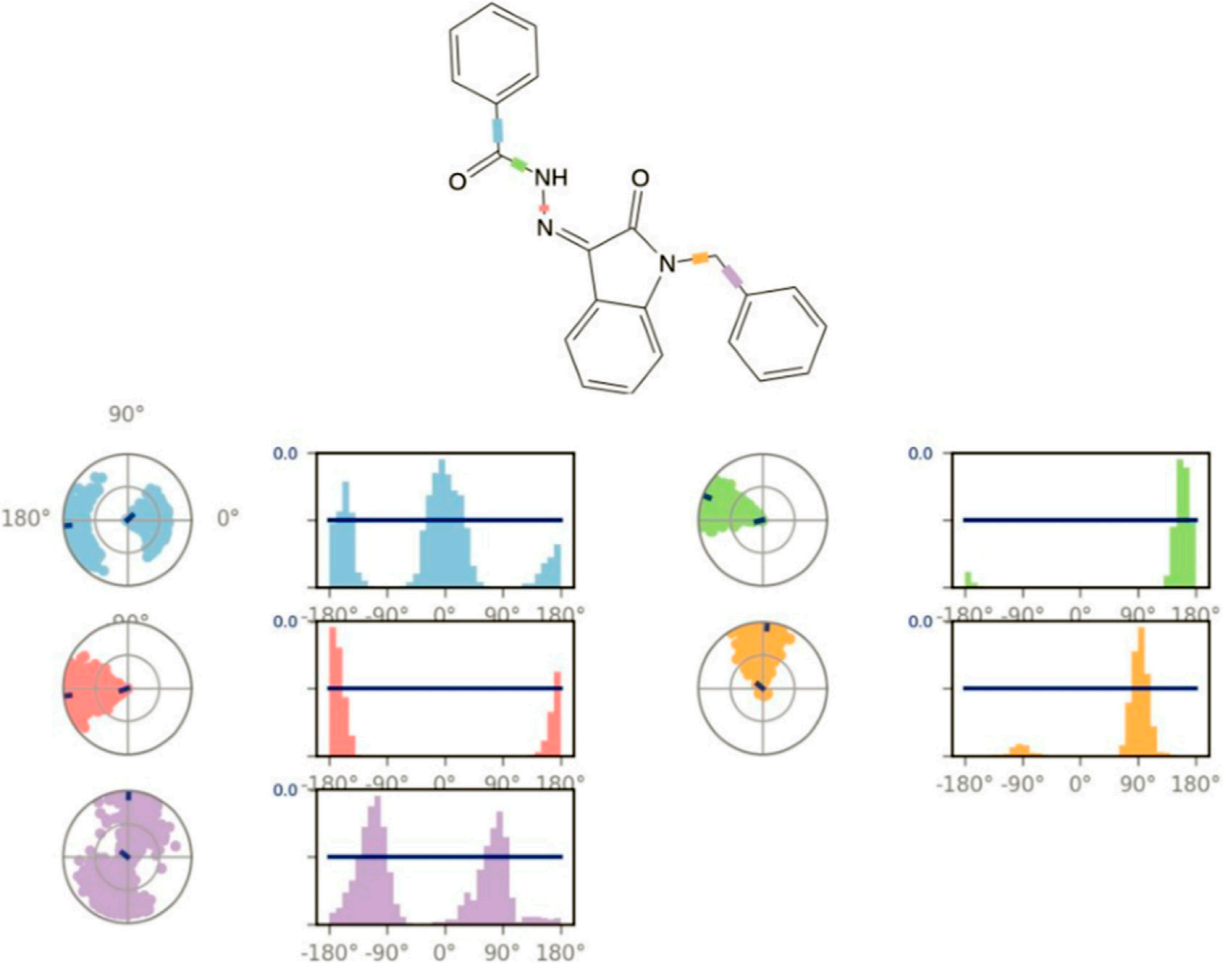
Ligand torsion profile.

Moreover, THR347, which did not show any interaction during the molecular docking phase, shows interactions during MD simulations. Such an event can be related to the dynamic nature of the environment and the presence of water molecules. THR347 creates a water bridge and then makes contact with the ligand atom, as shown in Figure 13.

Figure 13 displays the torsional profile of compound 5i obtained from MD simulations. The compound is made up of five rotatable bonds, each color-coded for clarity. Two types of plots are depicted in this figure: bar graphs and radial charts. These plots illustrate the distribution of torsional angles and the conformation of torsion, respectively, during the simulation (0–200 ns). The radial and line plots of the rotatable bond between the benzene ring and CH_2_ (in purple) show a significant degree of rotational freedom, with the bond rotating almost completely by 180° in both negative and positive *x*-axes. Furthermore, in both cases of positive and negative *x*-axes, a complete rotation of 180° in the bond between the carbonyl carbon and benzene ring is also shown in both the bar and radial plots (shown in blue).

In addition, the rotatable bond between the nitrogens of the hydrazide (pink) rotates approximately 90° around the negative *x*-axis. Similarly, the rotatable bond located between indolidine and CH_2_ (marked in orange) has a specific rotation of approximately 90° with both positive and negative *x*-axes. Additionally, other rotatable bonds are color-coded, and their torsional plots are shown in both radial and bar charts for a thorough inspection.

## 4 Conclusion

In summary, a new series of isatin–hydrazide conjugates, 5a–5i, were designed, synthesized, and evaluated for their *in vitro* cytotoxicity in the BC cell line MCF-7. All the compounds displayed moderate-to- good growth inhibitory potential. Compound 5i was most active with an IC_50_ value 9.29 ± 0.97 µM. This compound was shown to downregulate ERα levels robustly. The molecular docking results suggested the potential binding ability of the synthesized compounds. Furthermore, the accuracy and reliability of this molecule to bind with ERα were confirmed through MD simulations. MD simulations revealed the significant stability of the synthesized compound within the active site of ERα. In addition to that, *in silico* ADMET studies indicated that the compound entails drug-like properties. Collectively, these findings imply that the investigated compounds can serve as promising candidates for developing potent SERDs like anticancer agents against ER+ BC.

## Data Availability

The original contributions presented in the study are included in the article/Supplementary Material; further inquiries can be directed to the corresponding authors.

## References

[B1] Alajmi M. F. Rehman M. T. Hussain A. Rather G. M. (2018). Pharmacoinformatics approach for the identification of Polo-like kinase-1 inhibitors from natural sources as anti-cancer agents. Int. J. Biol. Macromol. 116, 173–181. 10.1016/j.ijbiomac.2018.05.023 29738867

[B2] Bhatia N. Hazra S. Thareja S. (2023). Selective Estrogen receptor degraders (SERDs) for the treatment of breast cancer: an overview. Eur. J. Med. Chem. 256, 115422. 10.1016/j.ejmech.2023.115422 37163948

[B3] Branka A. (2000). Nosé-Hoover chain method for nonequilibrium molecular dynamics simulation. Phys. Rev. E 61, 4769–4773. 10.1103/PhysRevE.61.4769 11031517

[B4] Dandrial J. Singla R. Kumar M. Jaitak V. (2016). Recent developments of C-4 substituted coumarin derivatives as anticancer agents. Eur. J. Med. Chem. 119, 141–168. 10.1016/j.ejmech.2016.03.087 27155469

[B5] Ding Z. Zhou M. Zeng C. (2020). Recent advances in isatin hybrids as potential anticancer agents. Arch. Pharm. 353, 1900367. 10.1002/ardp.201900367 31960987

[B6] Duffy M. J. (2006). Estrogen receptors: role in breast cancer. Crit. Rev. Clin. Lab. Sci. 43, 325–347. 10.1080/10408360600739218 16769596

[B7] El-faham A. Elzathahery A. A. Al-Othman Z. A. Elsayed E. A. (2014). Facile method for the synthesis of silver nanoparticles using 3-hydrazino-isatin derivatives in aqueous methanol and their antibacterial activity. Int. J. Nanomedicine 9, 1167–1174. 10.2147/IJN.S58571 24623975 PMC3949700

[B8] Fares M. Eldehna W. M. Abou-seri S. M. Abdel‐Aziz H. A. Aly M. H. Tolba M. F. (2015). Design, synthesis and *in vitro* antiproliferative activity of novel isatin‐quinazoline hybrids. Arch. Pharm. 348, 144–154. 10.1002/ardp.201400337 25664631

[B9] Ferraz De Paiva R. E. Vieira E. G. Rodrigues da silva D. Wegermann C. A. Costa Ferreira A. M. (2021). Anticancer compounds based on isatin-derivatives: strategies to ameliorate selectivity and efficiency. Front. Mol. Biosci. 7, 627272. 10.3389/fmolb.2020.627272 33614708 PMC7889591

[B10] Firdous F. Riaz S. Furqan M. Fozail S. Fatima K. Pohl S. O. T.-G. (2023). Design, synthesis, and biological evaluation of SSE1806, a microtubule destabilizer that overcomes multidrug resistance. ACS Med. Chem. Lett. 14, 1369–1377. 10.1021/acsmedchemlett.3c00258 37849542 PMC10577696

[B11] Fujiki N. Konno H. Kaneko Y. Gohno T. Hanamura T. Imami K. (2014). Estrogen response element-GFP (ERE-GFP) introduced MCF-7 cells demonstrated the coexistence of multiple estrogen-deprivation resistant mechanisms. J. Steroid Biochem. Mol. Biol. 139, 61–72. 10.1016/j.jsbmb.2013.08.012 24128438

[B12] Guissi N. E. I. Li H. Xu Y. Semcheddine F. Chen M. Su Z. (2017). Mitoxantrone-and folate-TPGS2k conjugate hybrid micellar aggregates to circumvent toxicity and enhance efficiency for breast cancer therapy. Mol. Pharm. 14, 1082–1094. 10.1021/acs.molpharmaceut.6b01009 28191959

[B13] Gupta V. K. Bhalla Y. Jaitak V. (2014). Impact of ABC transporters, glutathione conjugates in MDR and their modulation by flavonoids: an overview. Med. Chem. Res. 23, 1–15. 10.1007/s00044-013-0612-6

[B14] Guzowski J. R. Delaney E. J. Humora M. J. Irdam E. Kiesman W. F. Kwok A. (2012). Understanding and control of dimethyl sulfate in a manufacturing process: kinetic modeling of a fischer esterification catalyzed by H2SO4. Org. Process Res. Dev. 16, 232–239. 10.1021/op200323j

[B15] Han S. J. Begum K. Foulds C. E. Hamilton R. A. Bailey S. Malovannaya A. (2016). The dual estrogen receptor α inhibitory effects of the tissue-selective estrogen complex for endometrial and breast safety. Mol. Pharmacol. 89, 14–26. 10.1124/mol.115.100925 26487511 PMC4702103

[B16] Harris H. A. Albert L. M. Leathurby Y. Malamas M. S. Mewshaw R. E. Miller C. P. (2003). Evaluation of an estrogen receptor-β agonist in animal models of human disease. Endocrinology 144, 4241–4249. 10.1210/en.2003-0550 14500559

[B17] Hiscox S. Morgan L. Green T. P. Barrow D. Gee J. NicholsonI R. I. (2006). Elevated Src activity promotes cellular invasion and motility in tamoxifen resistant breast cancer cells. Breast Cancer Res. Treat. 97, 263–274. 10.1007/s10549-005-9120-9 16333527

[B18] Ibrahim H. S. Abou-seri S. M. Tanc M. Elaasser M. M. Abdel-Aziz H. A. Supuran C. T. (2015). Isatin-pyrazole benzenesulfonamide hybrids potently inhibit tumor-associated carbonic anhydrase isoforms IX and XII. Eur. J. Med. Chem. 103, 583–593. 10.1016/j.ejmech.2015.09.021 26408817

[B19] Jeselsohn R. Guo H. Rees R. Barry W. Barlett C. Tung N. (2019). Abstract PD1-05: results from the phase Ib/II clinical trial of bazedoxifene and palbociclib in hormone receptor positive metastatic breast cancer. Cancer Res. 79, PD1. PD1-05. 10.1158/1538-7445.SABCS18-PD1-05

[B20] Khan K. M. Rasheed M. Ullah Z. Hayat S. Kaukab F. Choudhary M. I. (2003). Synthesis and *in vitro* leishmanicidal activity of some hydrazides and their analogues. Bioorg Med. Chem. 11, 1381–1387. 10.1016/S0968-0896(02)00611-9 12628664

[B21] Kumar S. Gu L. Palma G. Kaur M. Singh-Pillay A. Singh P. (2018). Design, synthesis, anti-proliferative evaluation and docking studies of 1 H-1, 2, 3-triazole tethered ospemifene–isatin conjugates as selective estrogen receptor modulators. New J. Chem. 42, 3703–3713. 10.1039/C7NJ04964A

[B22] Le Tourneau C. Raymond E. Faivre S. (2007). Sunitinib: a novel tyrosine kinase inhibitor. A brief review of its therapeutic potential in the treatment of renal carcinoma and gastrointestinal stromal tumors (GIST). Ther. Clin. Risk Manag. 3, 341–348. 10.2147/tcrm.2007.3.2.341 18360643 PMC1936316

[B23] Lewis-Wambi J. S. Kim H. Curpan R. Grigg R. Sarker M. A. Jordan V. C. (2011). The selective estrogen receptor modulator bazedoxifene inhibits hormone-independent breast cancer cell growth and down-regulates estrogen receptor α and cyclin D1. Mol. Pharmacol. 80, 610–620. 10.1124/mol.111.072249 21737572 PMC3187528

[B24] Lindsy R. Gallagher J. C. Kagan R. PickarI J. H. Constantine G. (2009). Efficacy of tissue-selective estrogen complex of bazedoxifene/conjugated estrogens for osteoporosis prevention in at-risk postmenopausal women. Fertil. Steril. 92, 1045–1052. 10.1016/j.fertnstert.2009.02.093 19635616

[B25] Lipinski C. A. (2004). Lead-and drug-like compounds: the rule-of-five revolution. Drug Discov. Today Technol. 1, 337–341. 10.1016/j.ddtec.2004.11.007 24981612

[B26] Manzoor S. Bilal A. Khan S. Ullah R. Iftikhar S. Emwas A.-H. (2018). Identification and characterization of SSE15206, a microtubule depolymerizing agent that overcomes multidrug resistance. Sci. Rep. 8, 3305. 10.1038/s41598-018-21642-0 29459693 PMC5818492

[B27] Martyna G. J. Tobias D. J. Klein M. L. (1994). Constant pressure molecular dynamics algorithms. J. Chem. Phys. 101, 4177–4189. 10.1063/1.467468

[B28] Motzer R. J. MichaelsonI M. D. Redman B. G. Hudes G. R. Wilding G. Figin R. A. (2006). Activity of SU11248, a multitargeted inhibitor of vascular endothelial growth factor receptor and platelet-derived growth factor receptor, in patients with metastatic renal cell carcinoma. J. Clin. Oncol. 24, 16–24. 10.1200/jco.2005.02.2574 16330672

[B29] Pandeya S. Sriram D. Nath G. Declerc E. (1999). Synthesis, antibacterial, antifungal and anti-HIV activities of Schiff and Mannich bases derived from isatin derivatives and N-[4-(4′-chlorophenyl) thiazol-2-yl] thiosemicarbazide. Eur. J. Pharm. Sci. 9, 25–31. 10.1016/S0928-0987(99)00038-X 10493993

[B30] Patlewicz G. Jeliazkova N. Safford R. Worth A. Aleksiev B. (2008). An evaluation of the implementation of the Cramer classification scheme in the Toxtree software. Sar. QSAR Environ. Res. 19, 495–524. 10.1080/10629360802083871 18853299

[B31] Payton-Stewart F. Tilghman S. L. Williams L. G. Winfield L. L. (2014). Benzimidazoles diminish ERE transcriptional activity and cell growth in breast cancer cells. Biochem. Biophys. Res. Commun. 450, 1358–1362. 10.1016/j.bbrc.2014.06.130 24997336 PMC4190015

[B32] Pires D. E. Blundell T. L. Ascher D. B. (2015). pkCSM: predicting small-molecule pharmacokinetic and toxicity properties using graph-based signatures. J. Med. Chem. 58, 4066–4072. 10.1021/acs.jmedchem.5b00104 25860834 PMC4434528

[B33] Rim K.-T. (2020). *In silico* prediction of toxicity and its applications for chemicals at work. Toxicol. Environ. Health Sci. 12, 191–202. 10.1007/s13530-020-00056-4 32421081 PMC7223298

[B34] Rohini R. Reddy P. M. Shanker K. Kanthaiah K. Ravinder V. Hu A. (2011). Synthesis of mono, bis-2-(2-arylideneaminophenyl) indole azomethines as potential antimicrobial agents. Arch. Pharm. Res. 34, 1077–1084. 10.1007/s12272-011-0705-z 21811914

[B35] Roth G. J. Heckel A. Colbatzky F. Handschuh S. Kley J. R. Lehmann-Lintz T. (2009). Design, synthesis, and evaluation of indolinones as triple angiokinase inhibitors and the discovery of a highly specific 6-methoxycarbonyl-substituted indolinone (BIBF 1120). J. Med. Chem. 52, 4466–4480. 10.1021/jm900431g 19522465

[B36] Samanta S. Pappula V. Dinda M. Adimurthy S. (2014). Transition metal-free oxidative esterification of benzylic alcohols in aqueous medium. Org. Biomol. Chem. 12, 9453–9456. 10.1039/C4OB01524J 25325738

[B37] Sander T. Freyss J. Von Korff M. Ruffner C. (2015). DataWarrior: an open-source program for chemistry aware data visualization and analysis. J. Chem. Inf. Model 55, 460–473. 10.1021/ci500588j 25558886

[B38] Selvam P. Murugesh N. Chandramohan M. Debyser Z. Witvrouw M. (2008). Design, synthesis and antiHIV activity of novel isatine-sulphonamides. Indian J. Pharm. Sci. 70, 779. 10.4103/0250-474X.49121 21369440 PMC3040873

[B39] Shoda T. Kato M. Harada R. Fujisato T. Okuhira K. Demizu Y. (2015). Synthesis and evaluation of tamoxifen derivatives with a long alkyl side chain as selective estrogen receptor down-regulators. Bioorg Med. Chem. 23, 3091–3096. 10.1016/j.bmc.2015.05.002 26003343

[B40] Sridhar S. K. Ramesh A. (2001). Synthesis and pharmacological activities of hydrazones, Schiff and Mannich bases of isatin derivatives. Biol. Pharm. Bull. 24, 1149–1152. 10.1248/bpb.24.1149 11642321

[B41] Sumpter W. C. (1944). The chemistry of isatin. Chem. Rev. 34, 393–434. 10.1021/cr60109a003

[B42] Veber D. F. Johnson S. R. Cheng H.-Y. Smith B. R. Ward K. W. Kopple K. D. (2002). Molecular properties that influence the oral bioavailability of drug candidates. J. Med. Chem. 45, 2615–2623. 10.1021/jm020017n 12036371

[B43] Verma M. Pandeya S. N. Singh K. N. Stables J. P. (2004). Anticonvulsant activity of Schiff bases of isatin derivatives. Acta Pharm. 54, 49–56. 15050044

[B44] Welsh A. W. Lannin D. R. Young G. S. Sherman M. E. FigueroaI J. D. Henry N. L. (2012). Cytoplasmic estrogen receptor in breast cancer. Clin. Cancer Res. 18, 118–126. 10.1158/1078-0432.CCR-11-1236 21980134 PMC3263348

[B45] Zamzami M. A. (2023). Molecular docking, molecular dynamics simulation and MM-GBSA studies of the activity of glycyrrhizin relevant substructures on SARS-CoV-2 RNA-dependent-RNA polymerase. J. Biomol. Struct. Dyn. 41, 1846–1858. 10.1080/07391102.2021.2025147 35037842

