# Supplementary material for: Discovery and prospects of new heterocyclic Isatin-hydrazide derivative with a novel role as estrogen receptor α degrader in breast cancer cells
Source: Front Chem. 2024 Jul 3;12:1424637. doi: 10.3389/fchem.2024.1424637 (PMC11252035; doi:10.3389/fchem.2024.1424637)

Figure S1: ^{13}C NMR spectrum of N'-(1-benzyl-2-oxo-1,2-dihydro-3H-indol-3-ylidene)pyridine-4-carbohydrazide (5a)

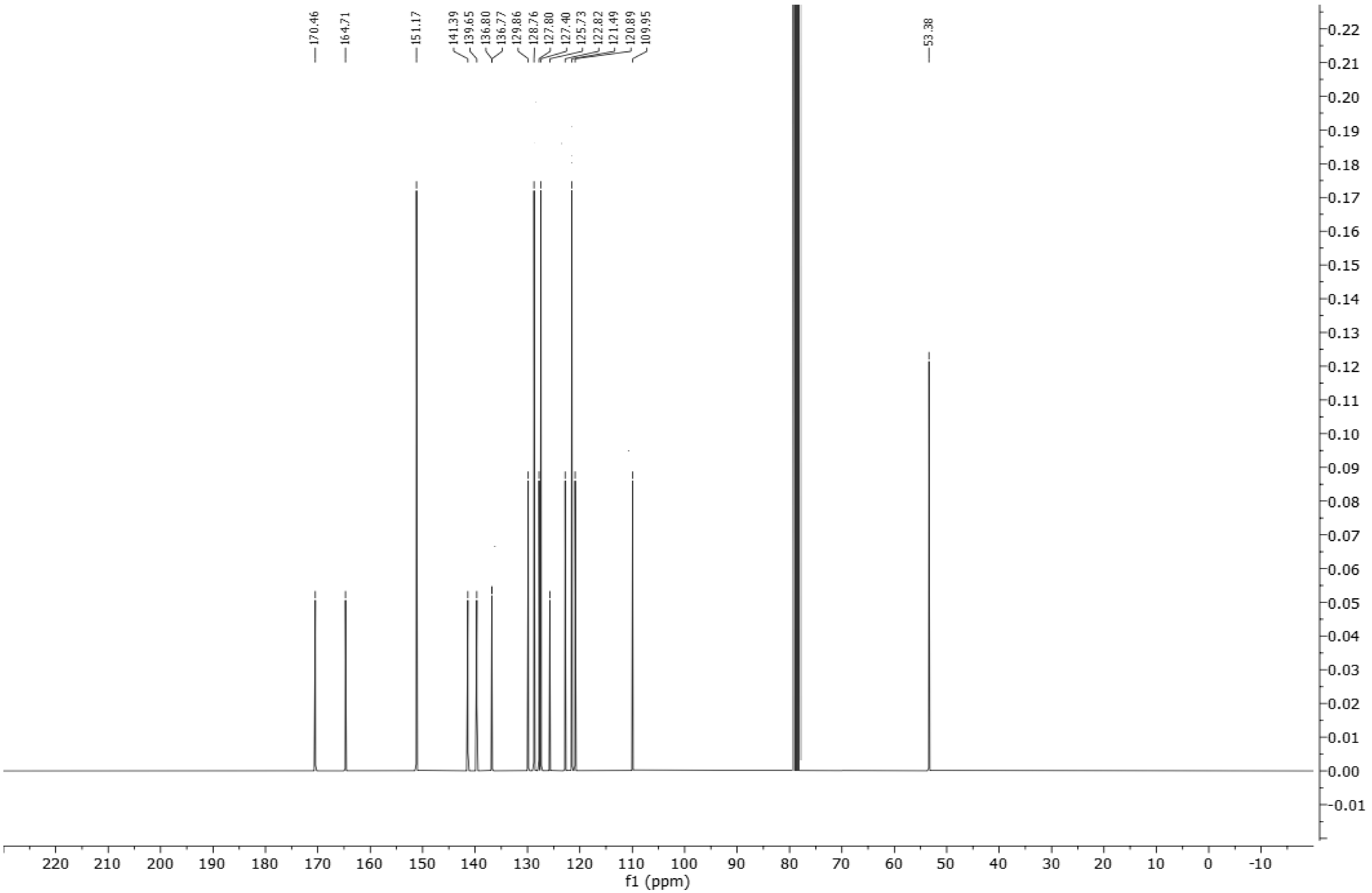

Figure S2: ^{1}H NMR spectrum of N'-(1-benzyl-2-oxo-1,2-dihydro-3H-indol-3-ylidene)pyridine-4-carbohydrazide (5a)

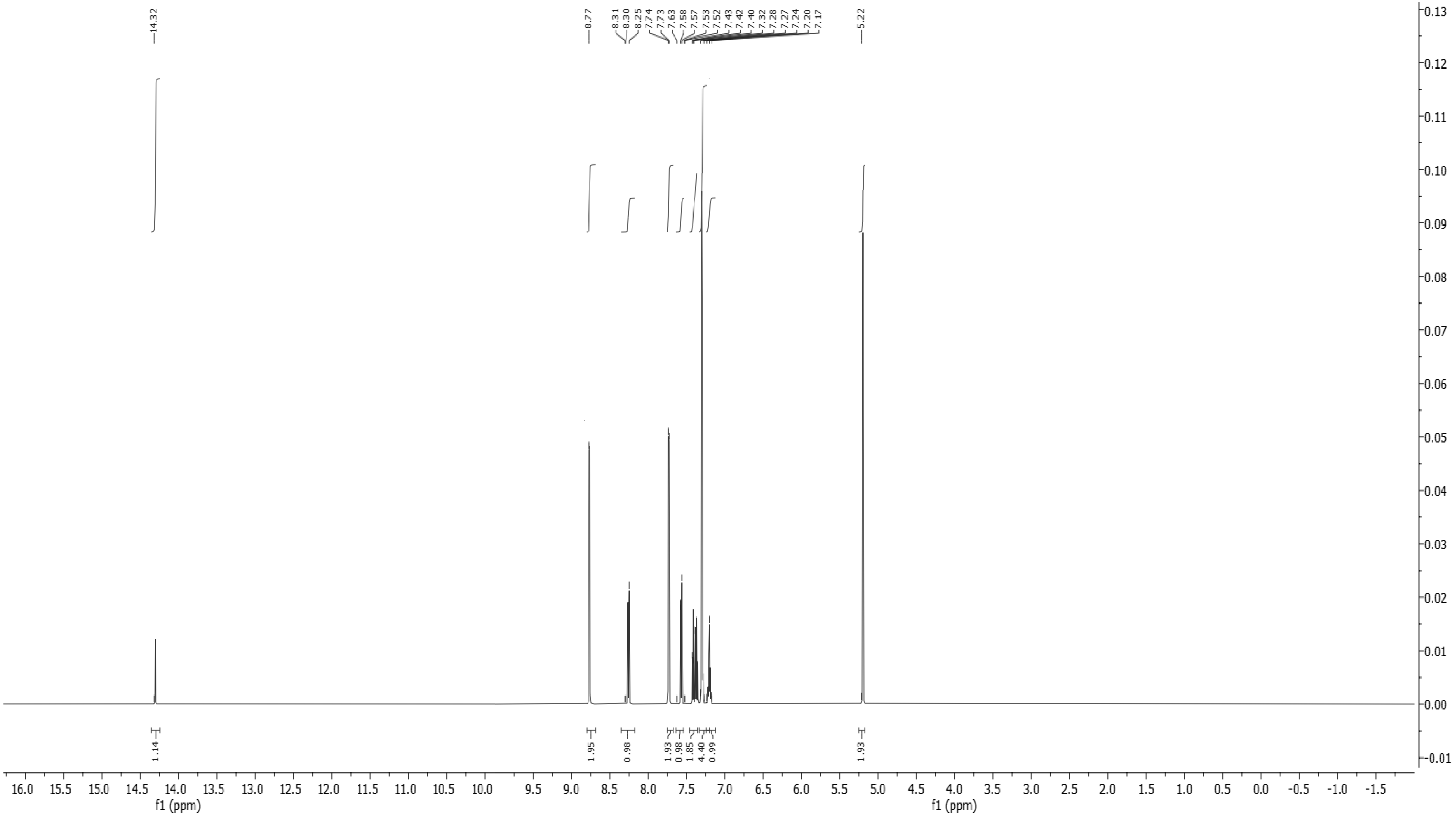

Figure S3: ^{13}C NMR spectrum of N'-(1-benzyl-2-oxo-1,2-dihydro-3H-indol-3-ylidene)-3-bromobenzohydrazide (5b)

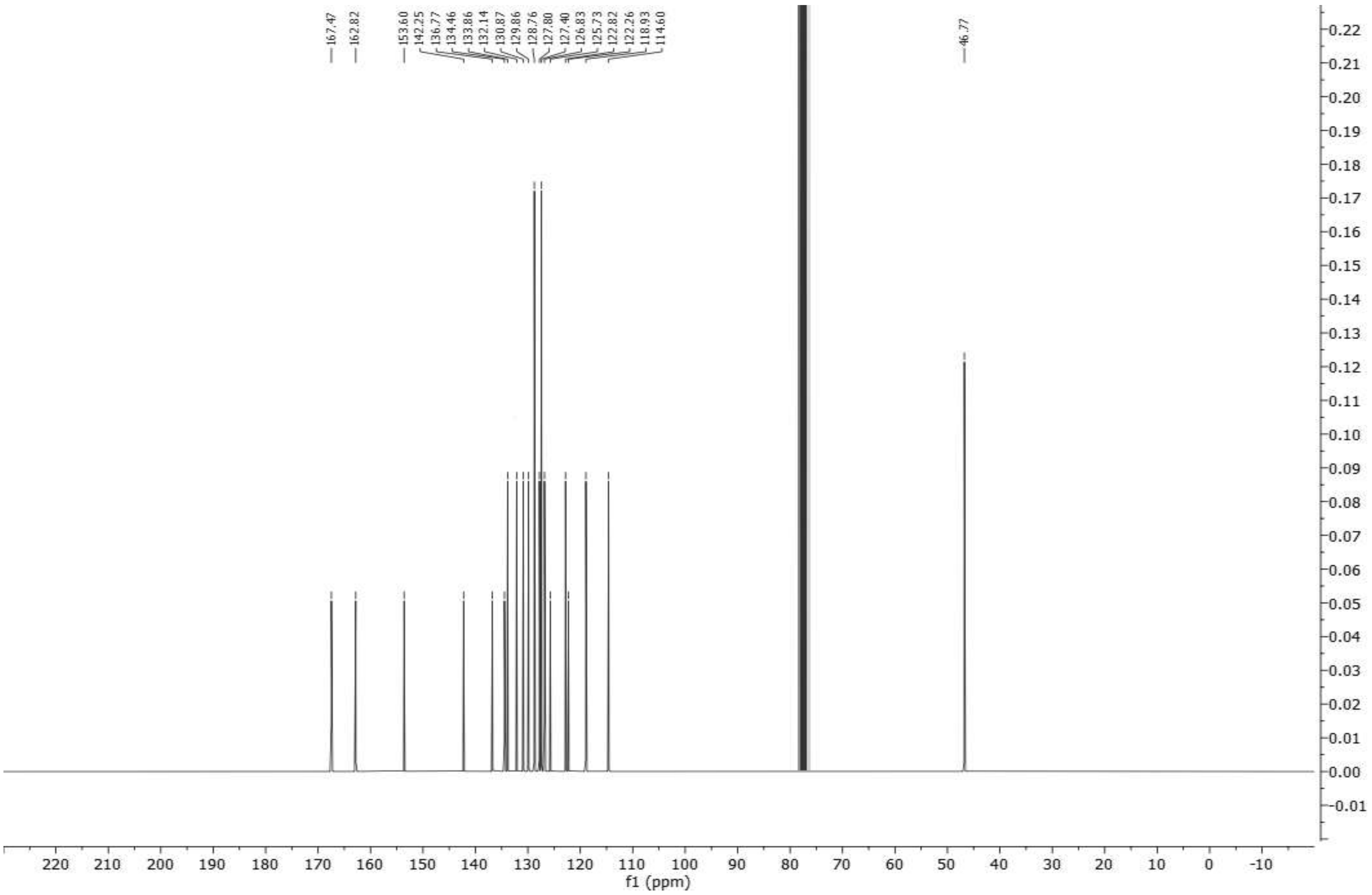

Figure S4: ^{1}H NMR spectrum of N'-(1-benzyl-2-oxo-1,2-dihydro-3H-indol-3-ylidene)-3-bromobenzohydrazide (5b)

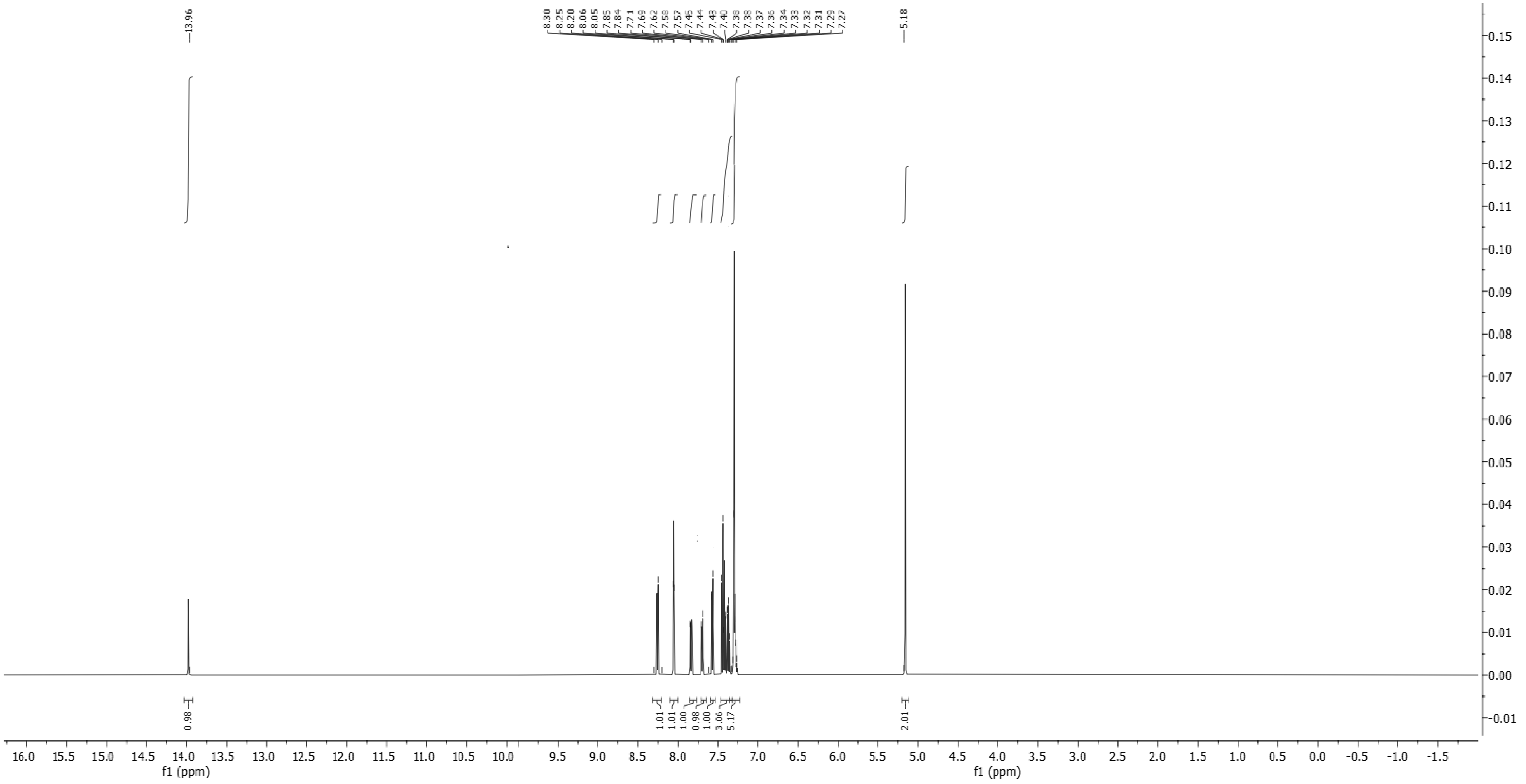

Figure S5: ^{13}C NMR spectrum of N'-(1-benzyl-2-oxo-1,2-dihydro-3H-indol-3-ylidene)-3-iodobenzohydrazide (5c)

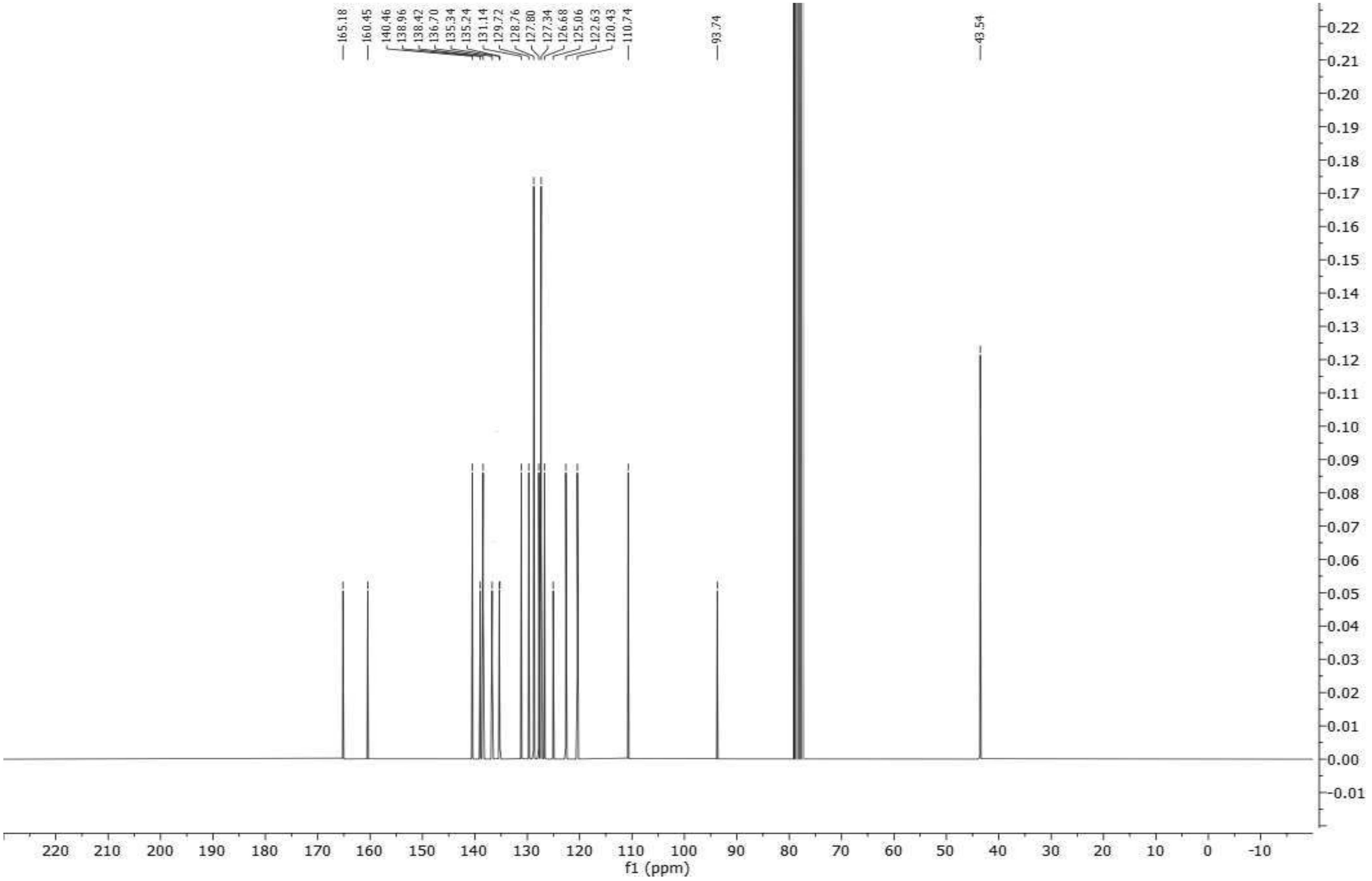

Figure S6: ^{1}H NMR spectrum of N'-(1-benzyl-2-oxo-1,2-dihydro-3H-indol-3-ylidene)-3-iodobenzohydrazide (5c)

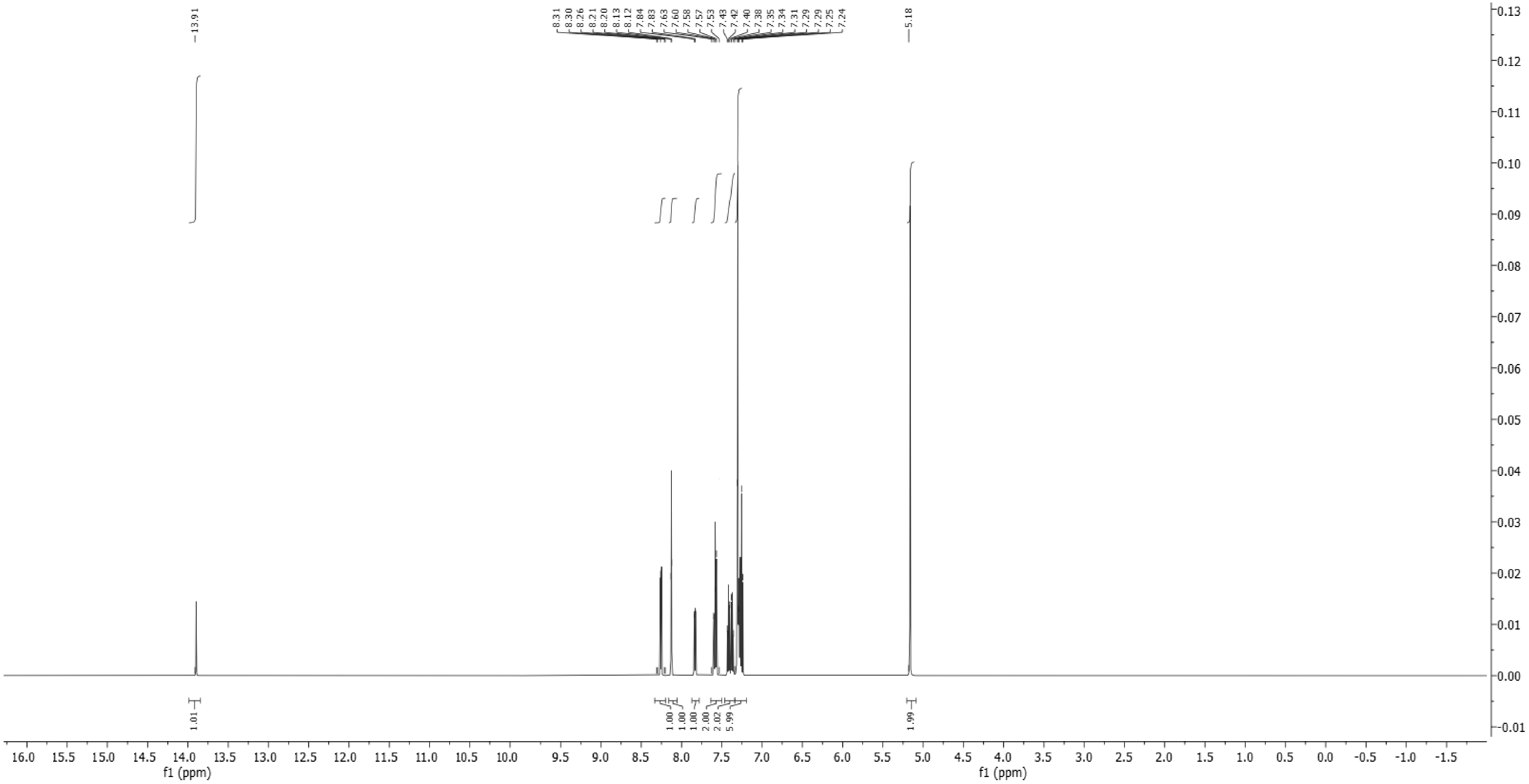

Figure S7: ^{13}C NMR spectrum of N'-(1-benzyl-2-oxo-1,2-dihydro-3H-indol-3-ylidene)-2-hydroxybenzohydrazide (5d)

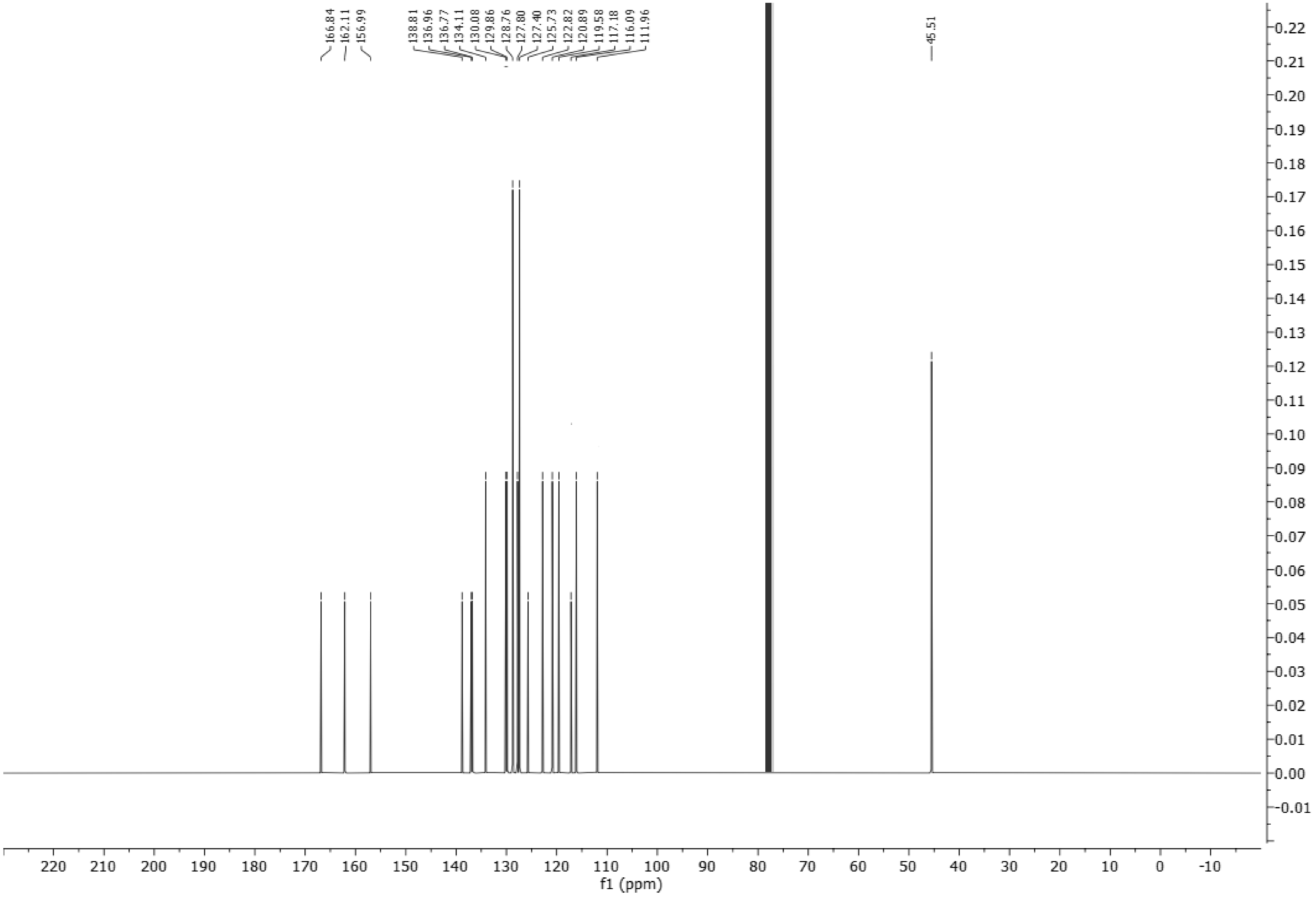

Figure S8: ^{1}H NMR spectrum of N'-(1-benzyl-2-oxo-1,2-dihydro-3H-indol-3-ylidene)-2-hydroxybenzohydrazide (5d)

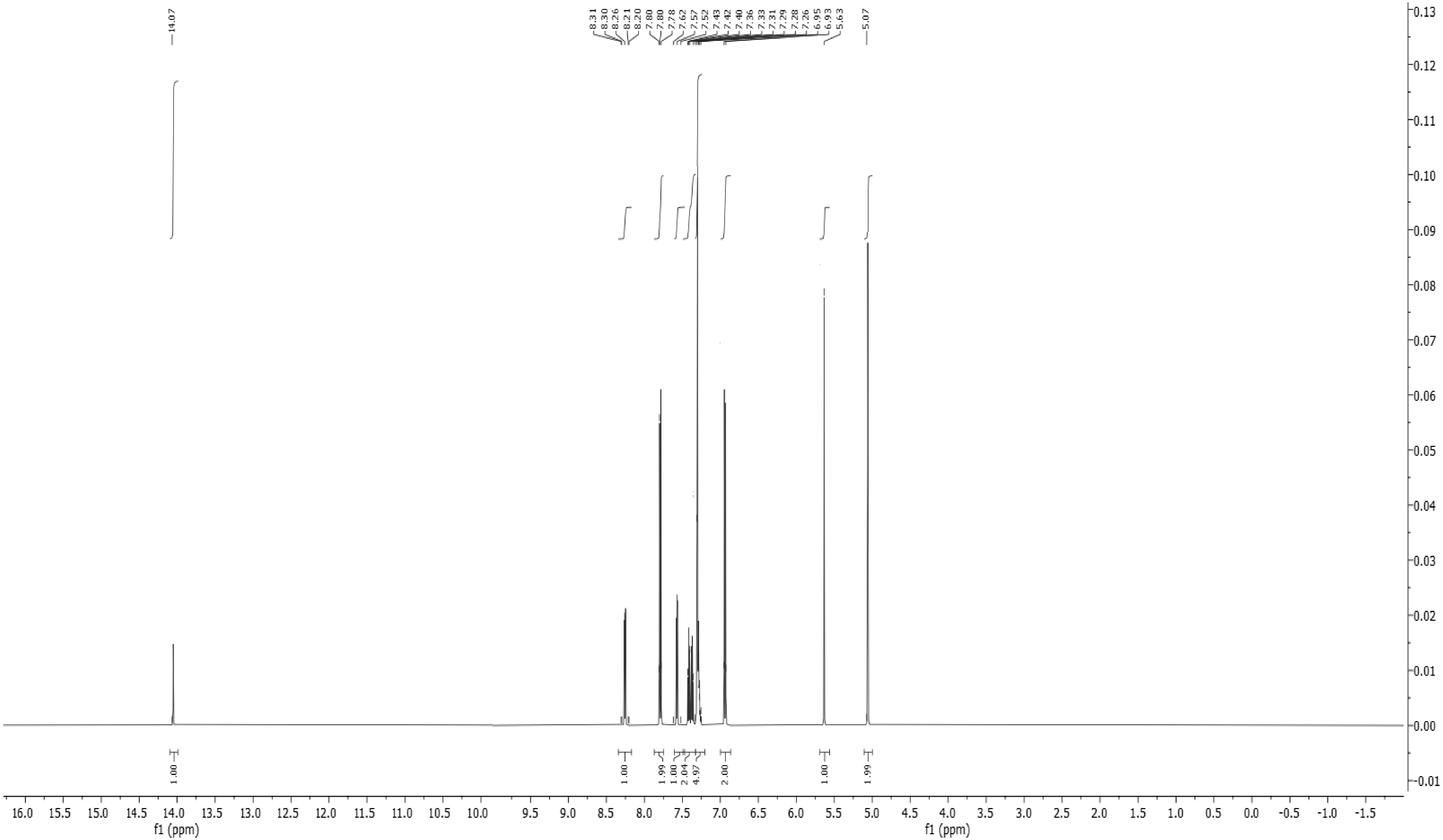

Figure S9: ^{13}C NMR spectrum of N'-(1-benzyl-2-oxo-1,2-dihydro-3H-indol-3-ylidene)-4-fluorobenzohydrazide (5e)

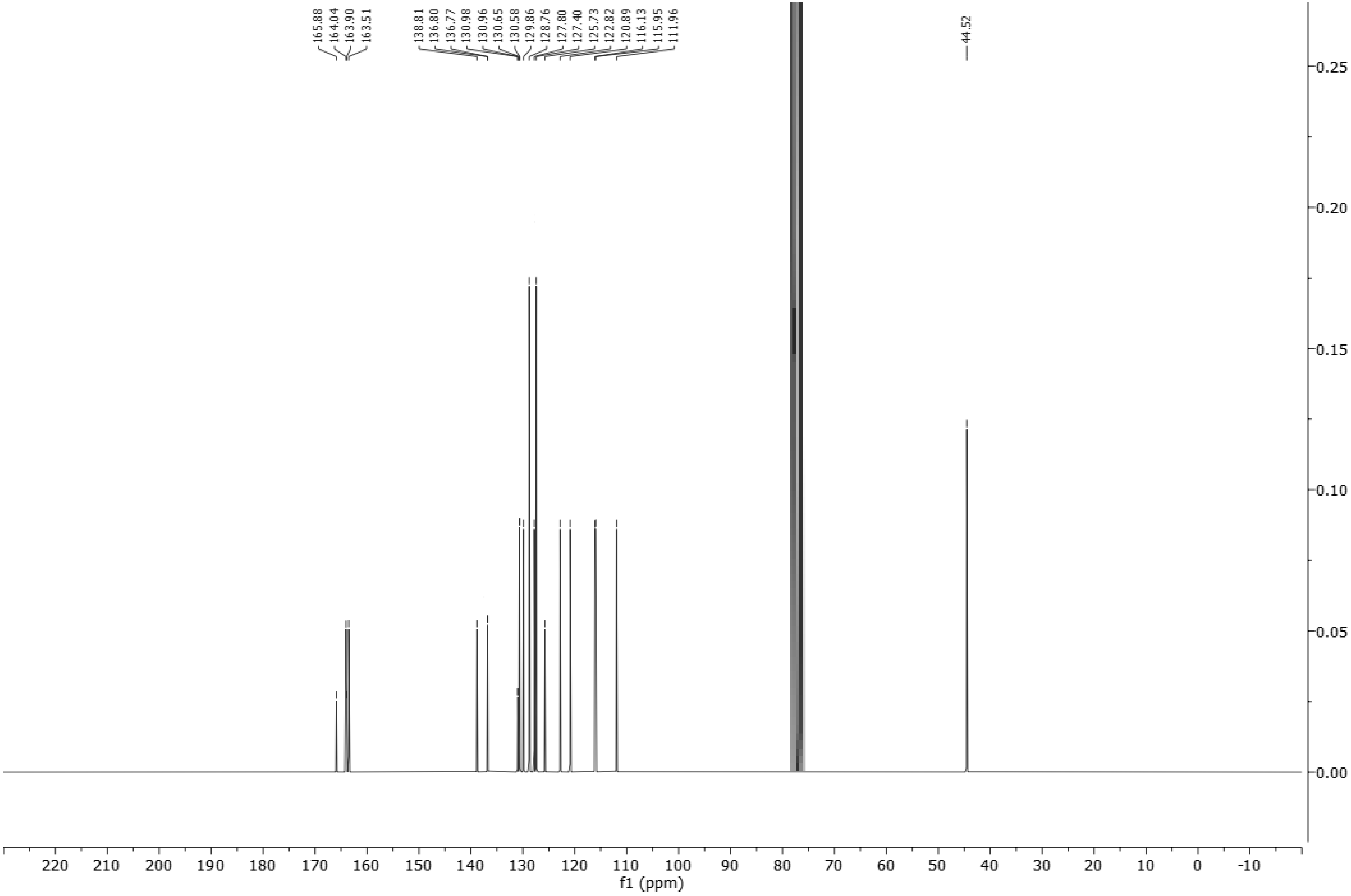

Figure S10: 1H NMR spectrum of N'-(1-benzyl-2-oxo-1,2-dihydro-3H-indol-3-ylidene)-4-fluorobenzohydrazide (5e)

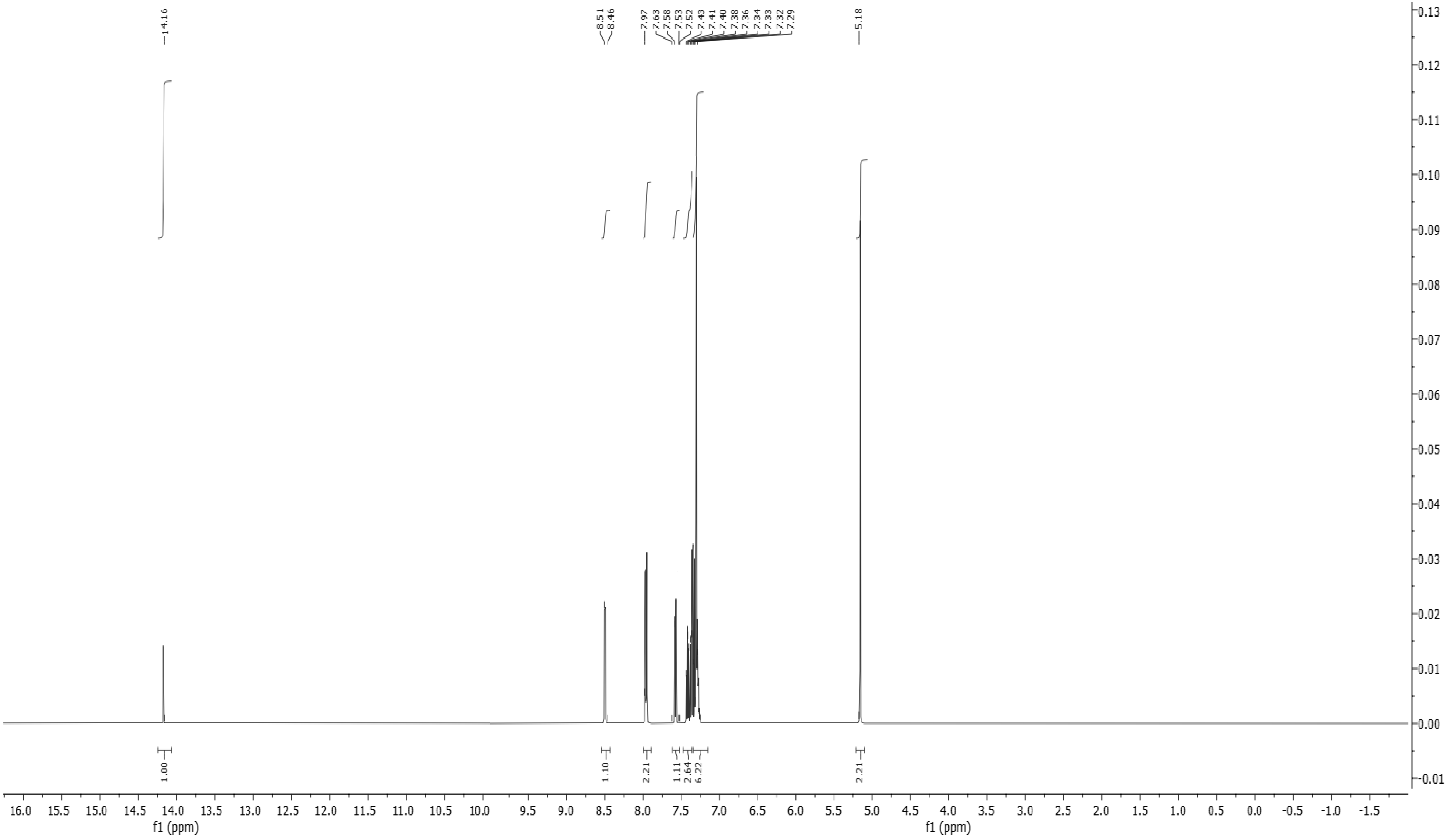

Figure S11: 13C NMR spectrum of N'-(1-benzyl-2-oxo-1,2-dihydro-3H-indol-3-ylidene)pyridine-2-carbohydrazide (5f):

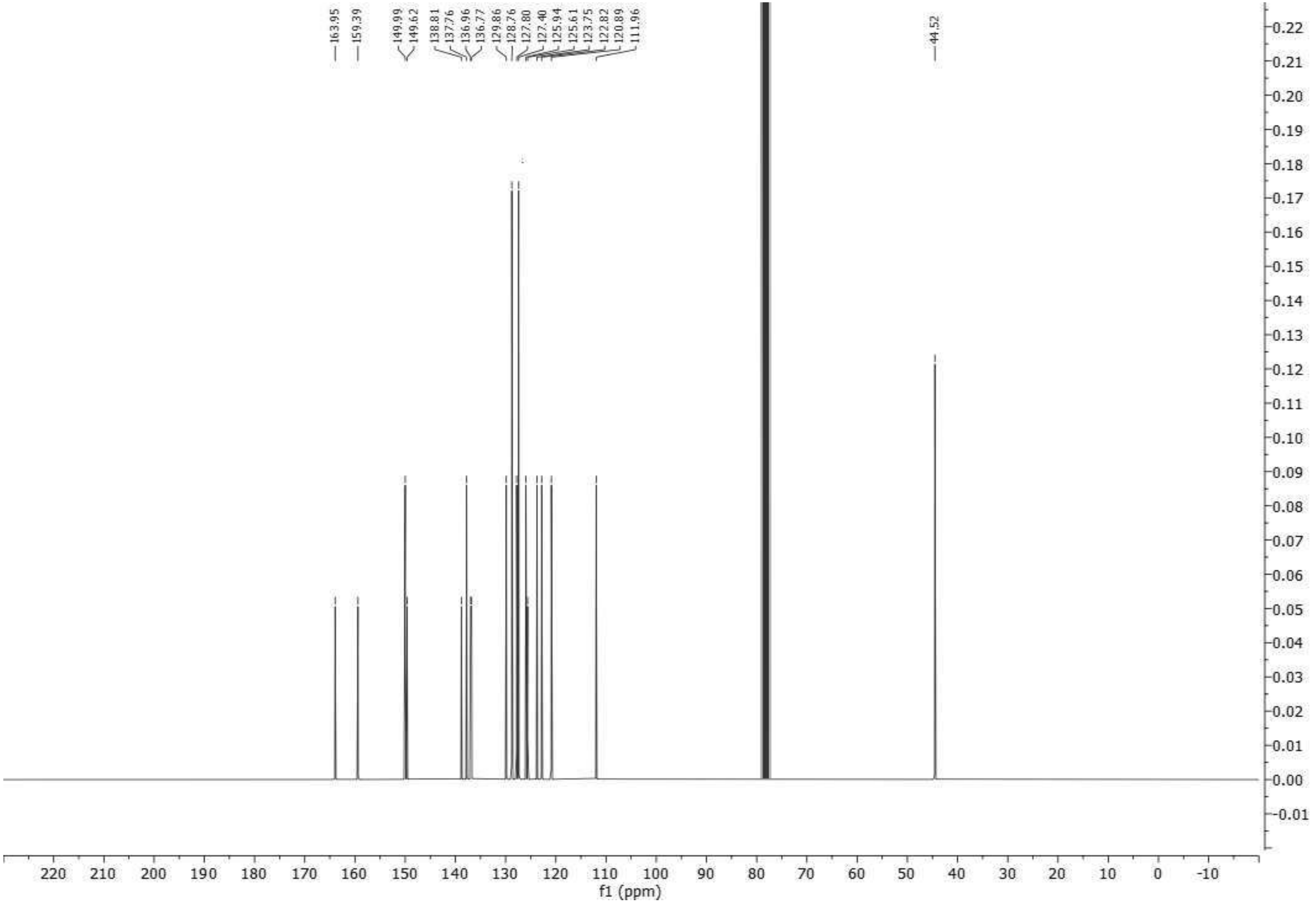

Figure S12: ^{1}H NMR spectrum of N'-(1-benzyl-2-oxo-1,2-dihydro-3H-indol-3-ylidene)pyridine-2-carbohydrazide (5f):

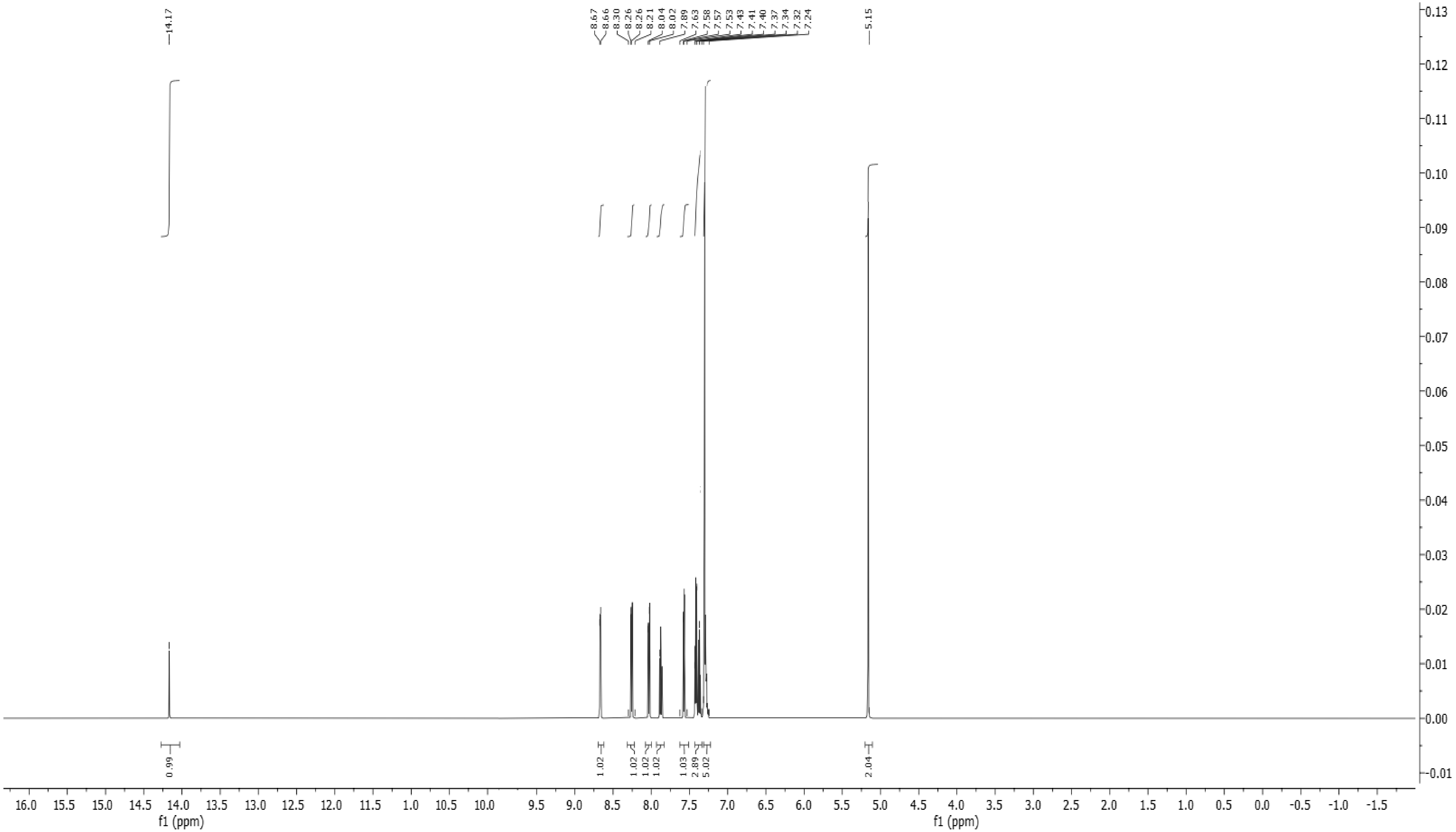

Figure S13: ^{13}C NMR spectrum of N'-(1-benzyl-2-oxo-1,2-dihydro-3H-indol-3-ylidene)-3,4,5trihydroxybenzohydrazide (5g):

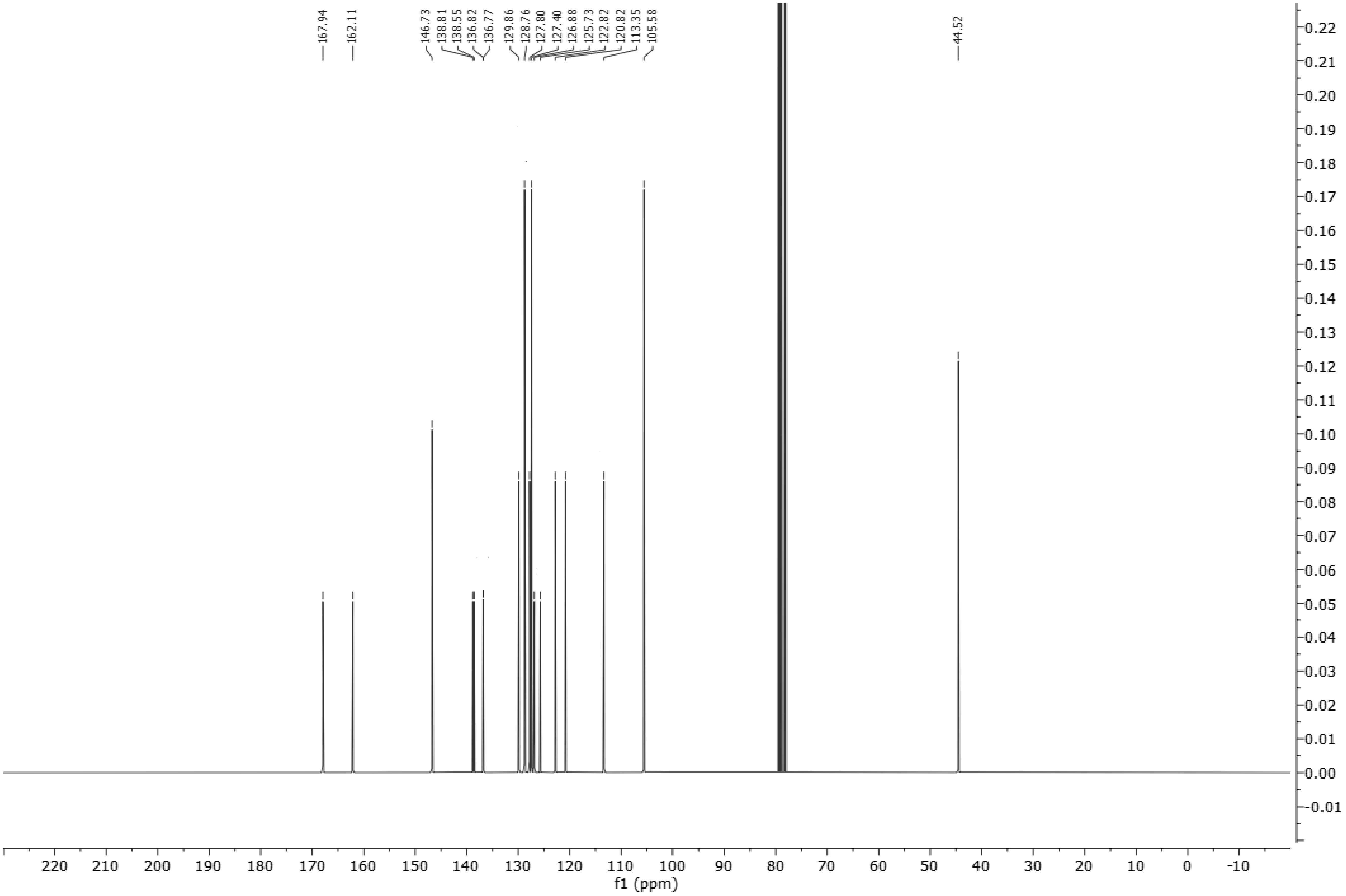

Figure S14: ^{1}H NMR spectrum of N'-(1-benzyl-2-oxo-1,2-dihydro-3H-indol-3-ylidene)-3,4,5trihydroxybenzohydrazide (5g):

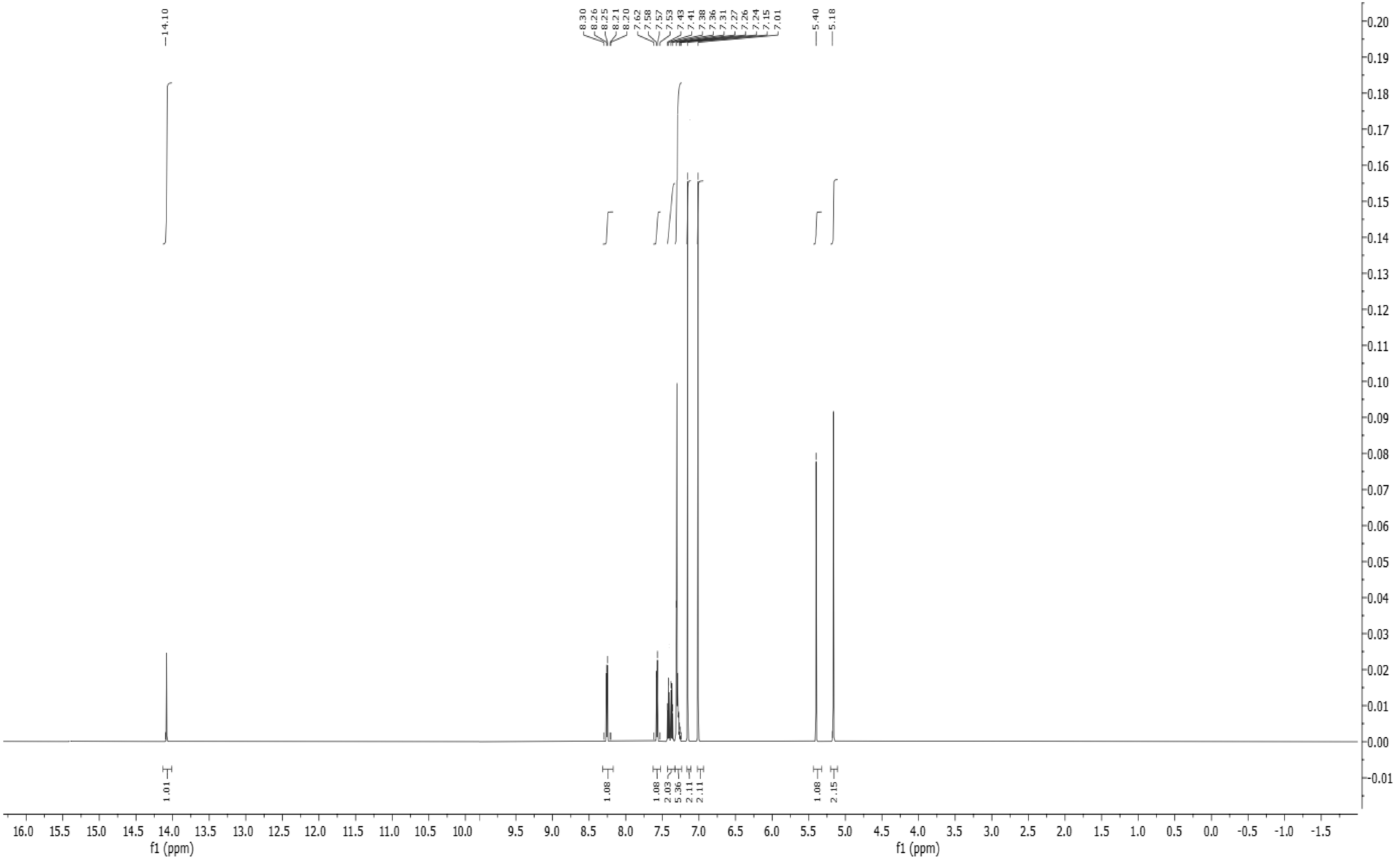

Figure S15: ^{13}C NMR spectrum of N'-(1-benzyl-2-oxo-1,2-dihydro-3H-indol-3-ylidene)-2-chlorobenzohydrazide (5h):

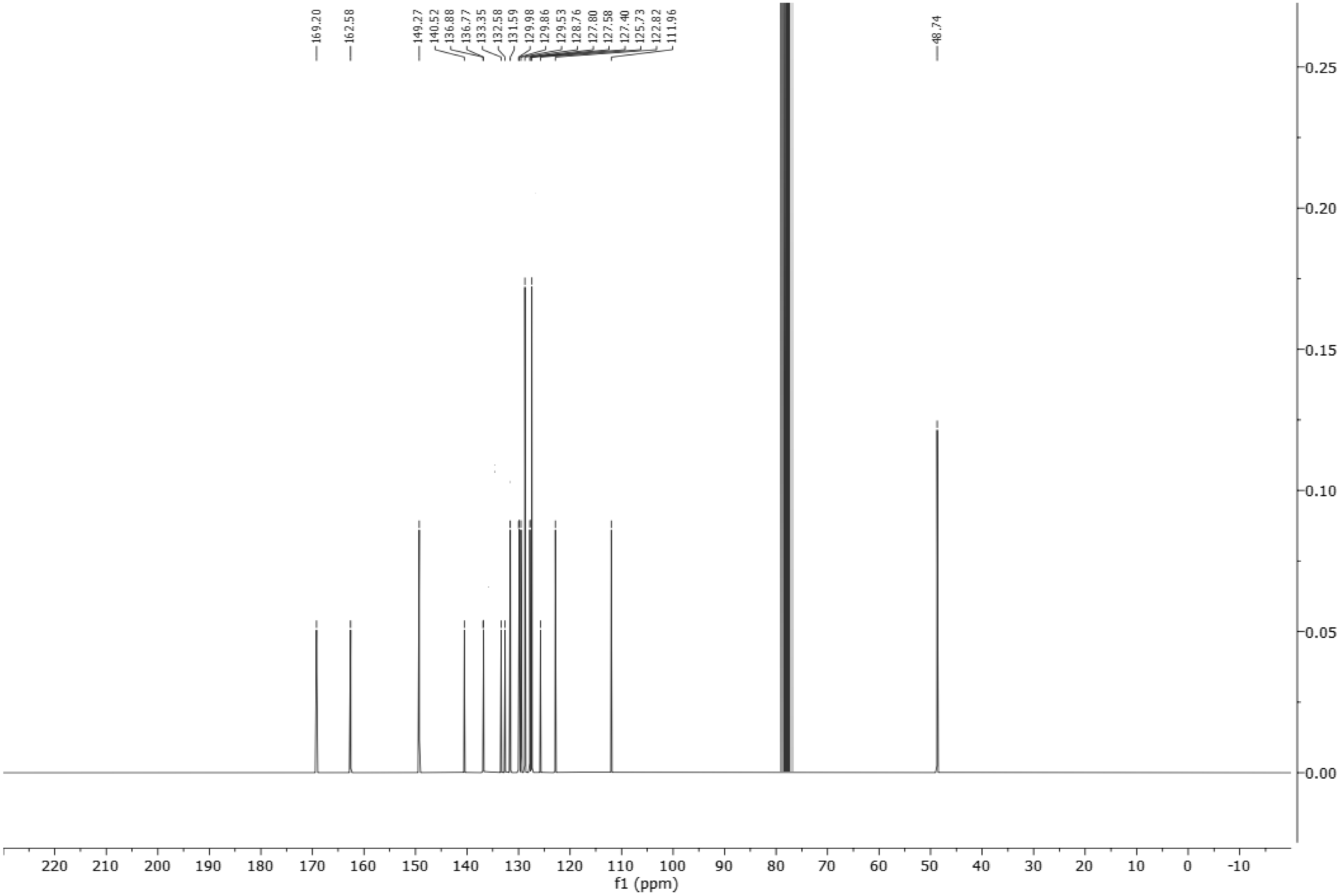

Figure S16: ^{1}H NMR spectrum of N'-(1-benzyl-2-oxo-1,2-dihydro-3H-indol-3-ylidene)-2-chlorobenzohydrazide (5h):

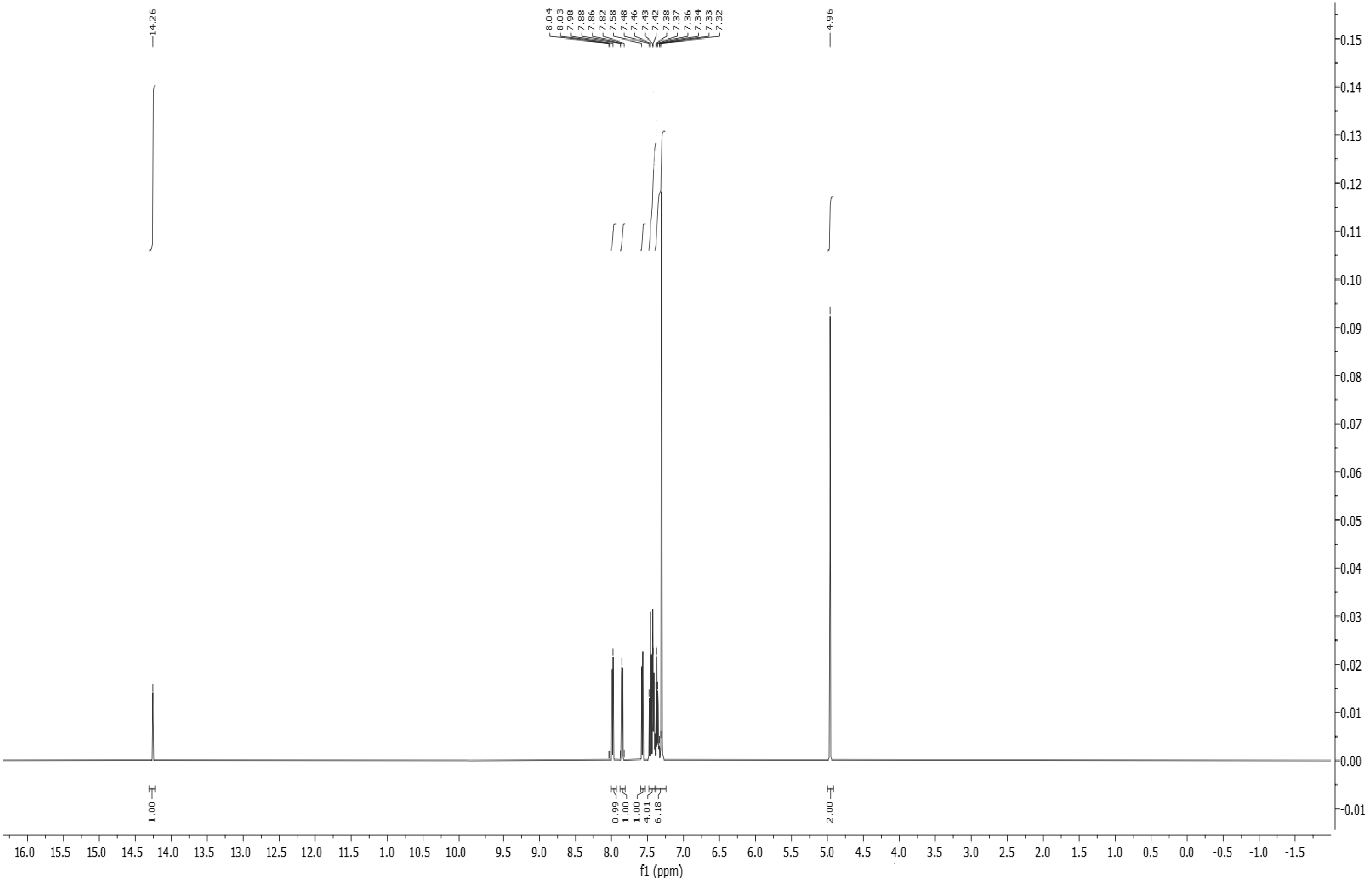

Figure S17: ^{13}C NMR spectrum of N'-(1-benzyl-2-oxo-1,2-dihydro-3H-indol-3-ylidene)benzohydrazide (5i):

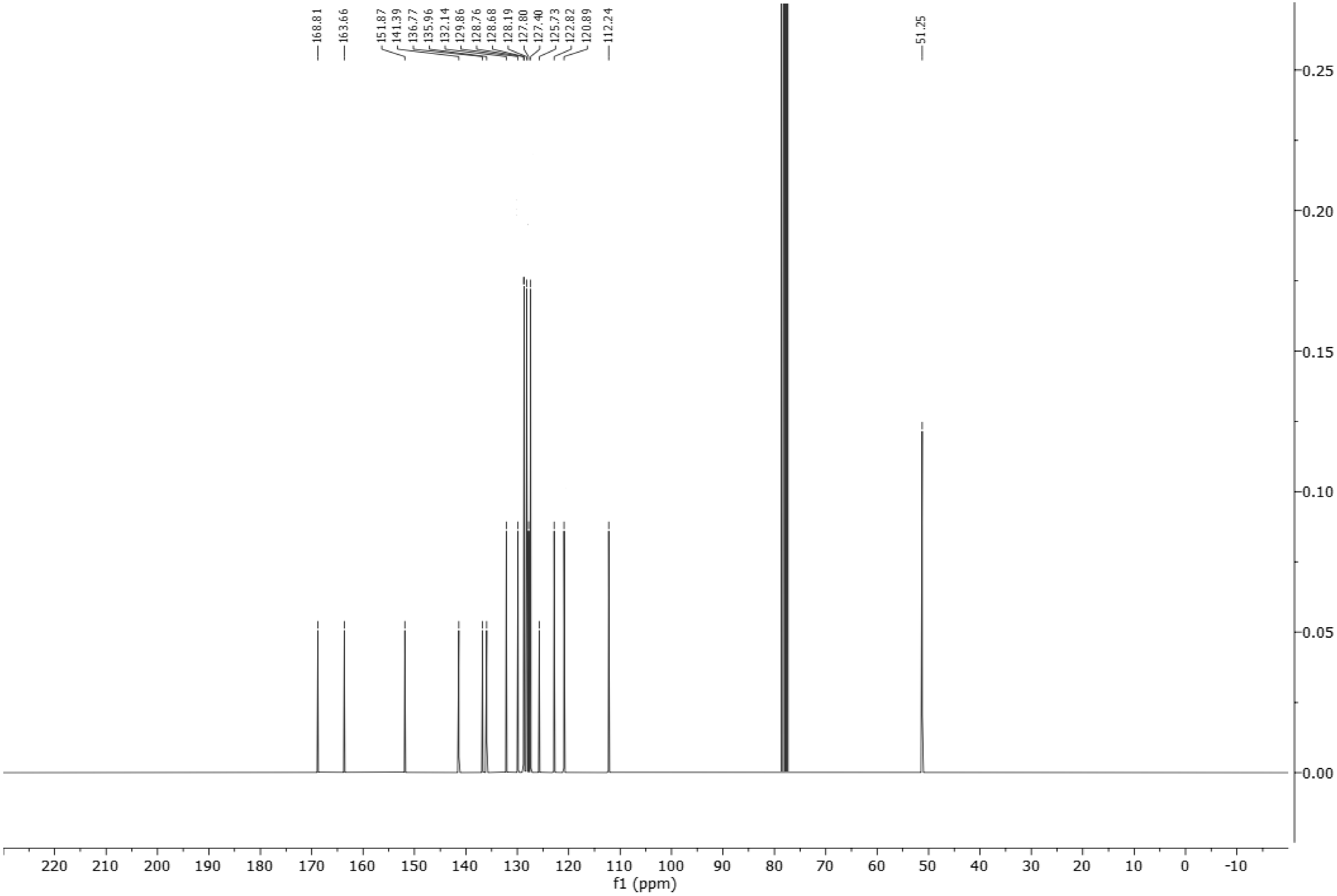

Figure S18: ^{1}H NMR spectrum of N'-(1-benzyl-2-oxo-1,2-dihydro-3H-indol-3-ylidene)benzohydrazide (5i):

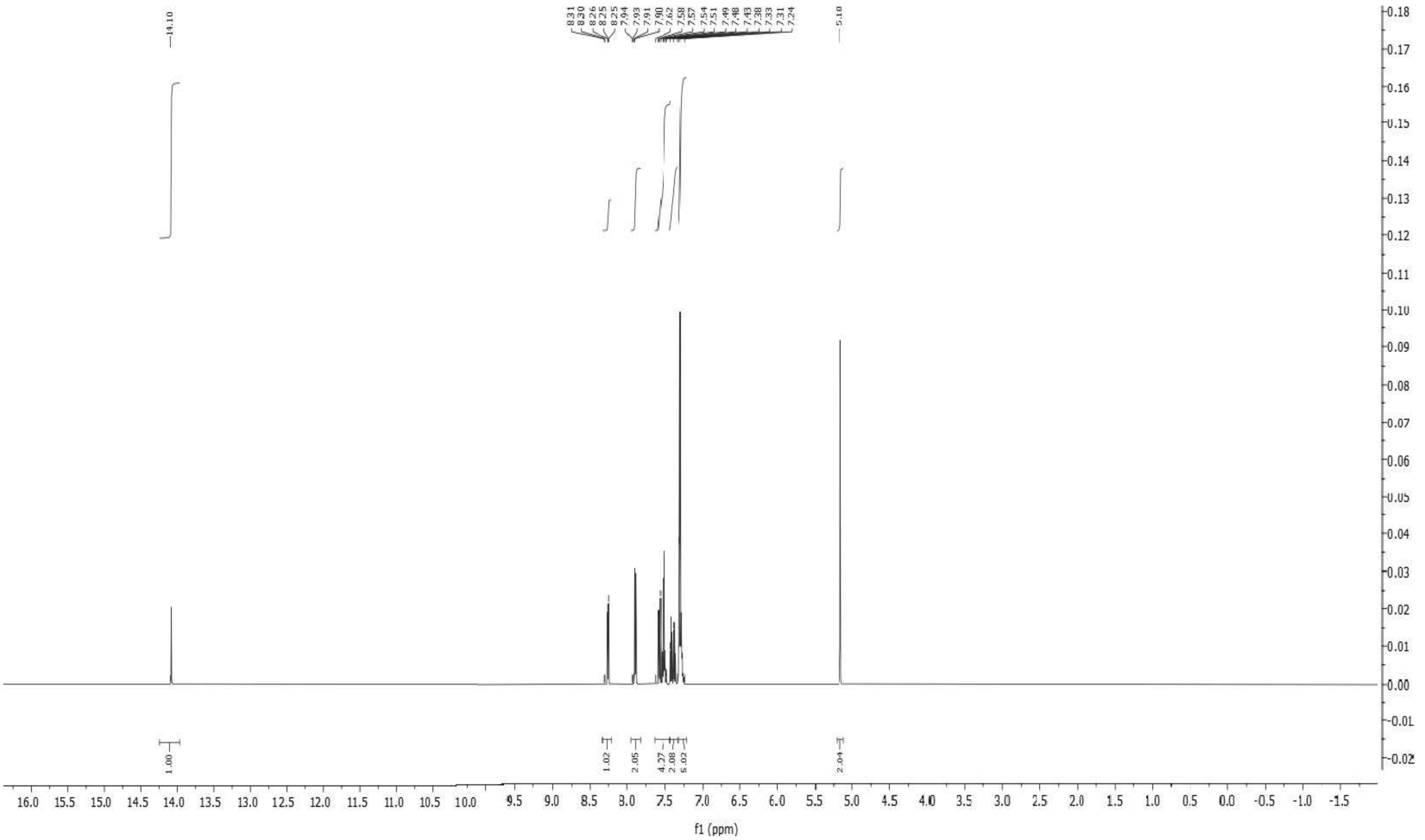

Figure S19: Mass spectrum of N'-(1-benzyl-2-oxo-1,2-dihydro-3H-indol-3-ylidene)pyridine-4-carbohydrazide (5a)

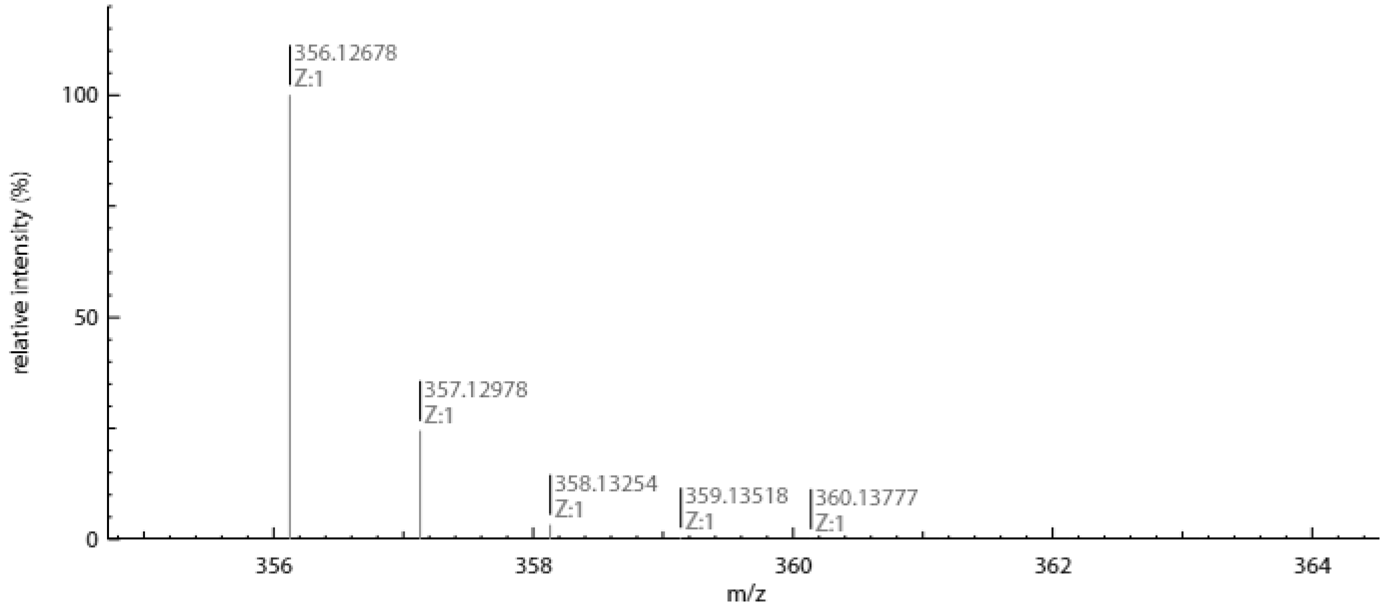

Figure S20: Mass spectrum of N'-(1-benzyl-2-oxo-1,2-dihydro-3H-indol-3-ylidene)-3-bromobenzohydrazide (5b)

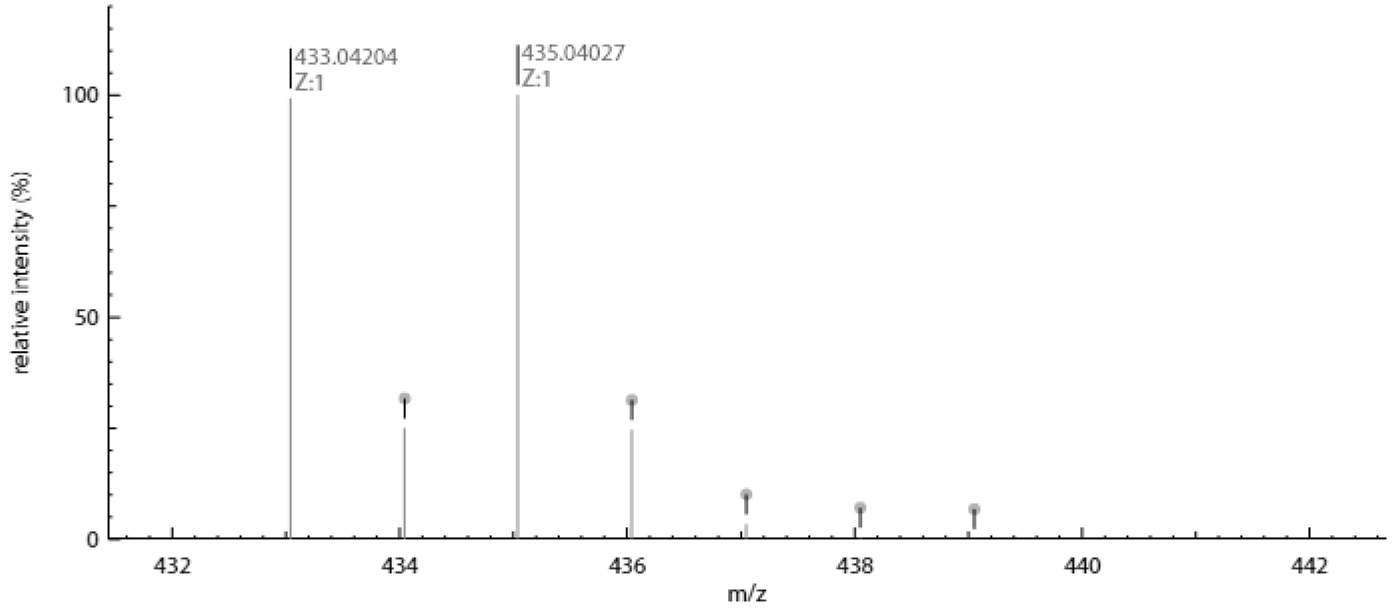

Figure S21: Mass spectrum of N'-(1-benzyl-2-oxo-1,2-dihydro-3H-indol-3-ylidene)-3-iodobenzohydrazide (5c)

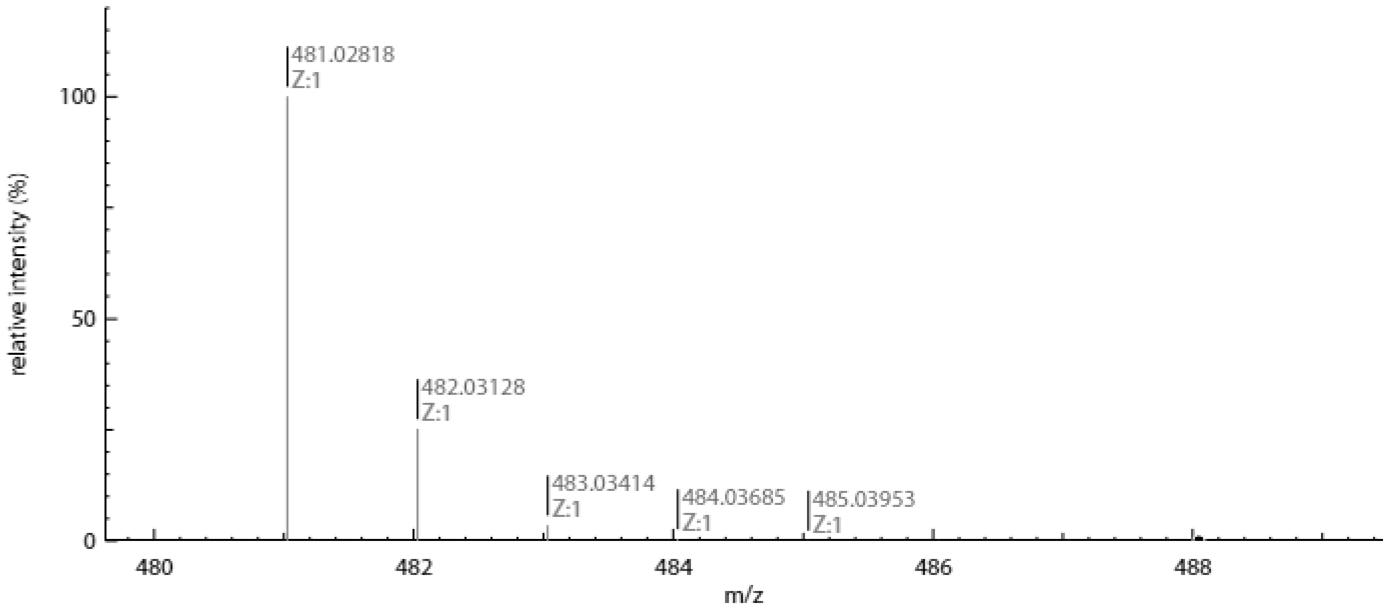

Figure S22: Mass spectrum of N'-(1-benzyl-2-oxo-1,2-dihydro-3H-indol-3-ylidene)-2-hydroxybenzohydrazide (5d)

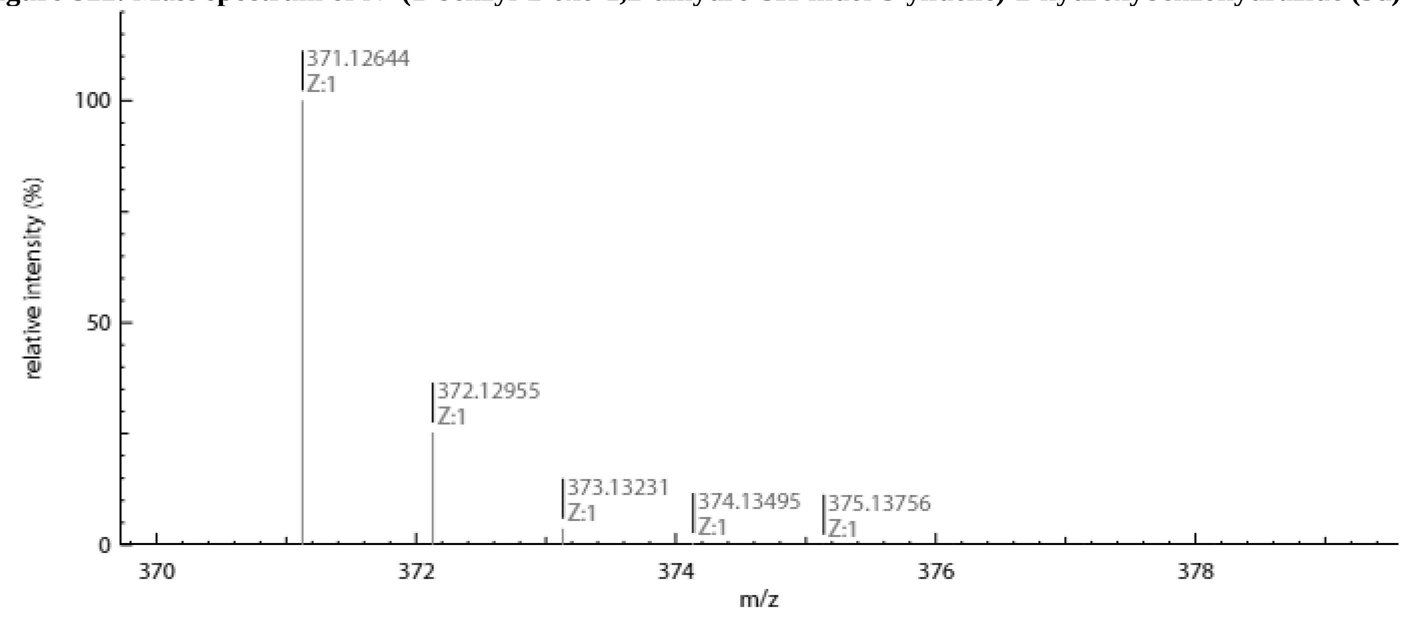

Figure S23: Mass spectrum of N'-(1-benzyl-2-oxo-1,2-dihydro-3H-indol-3-ylidene)-4-fluorobenzohydrazide (5e)

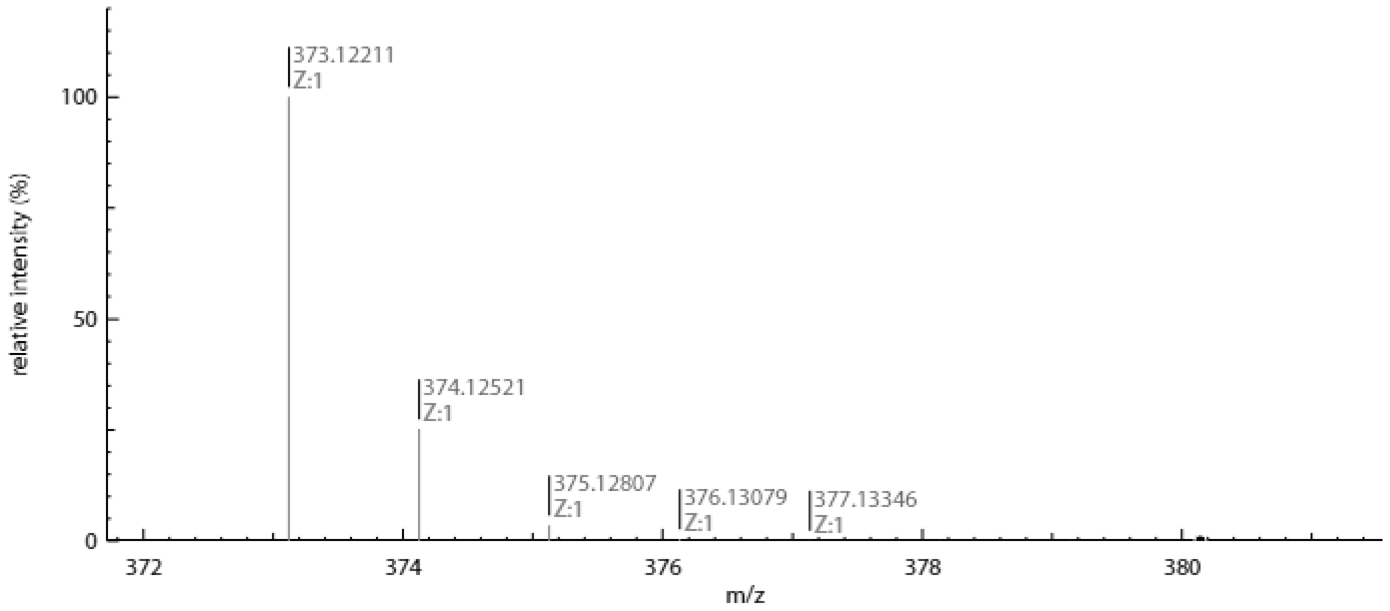

Figure S24: Mass spectrum of N'-(1-benzyl-2-oxo-1,2-dihydro-3H-indol-3-ylidene)pyridine-2-carbohydrazide (5f):

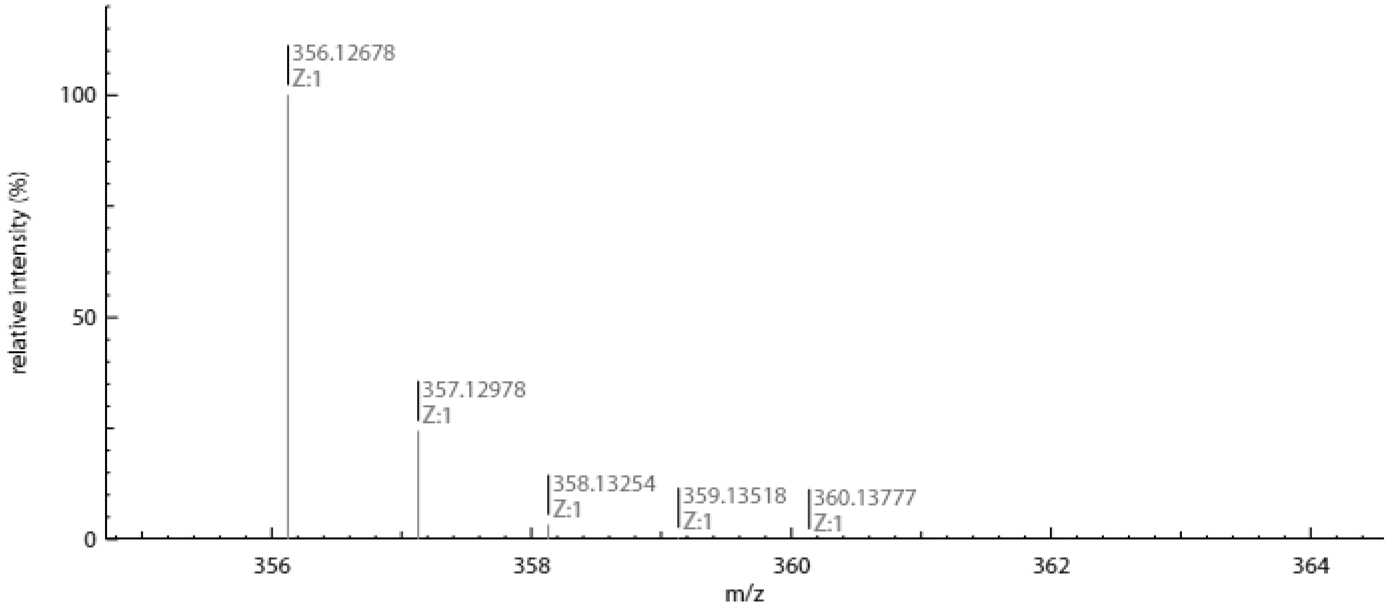

Figure S25: Mass spectrum of N'-(1-benzyl-2-oxo-1,2-dihydro-3H-indol-3-ylidene)-3,4,5trihydroxybenzohydrazide (5g):

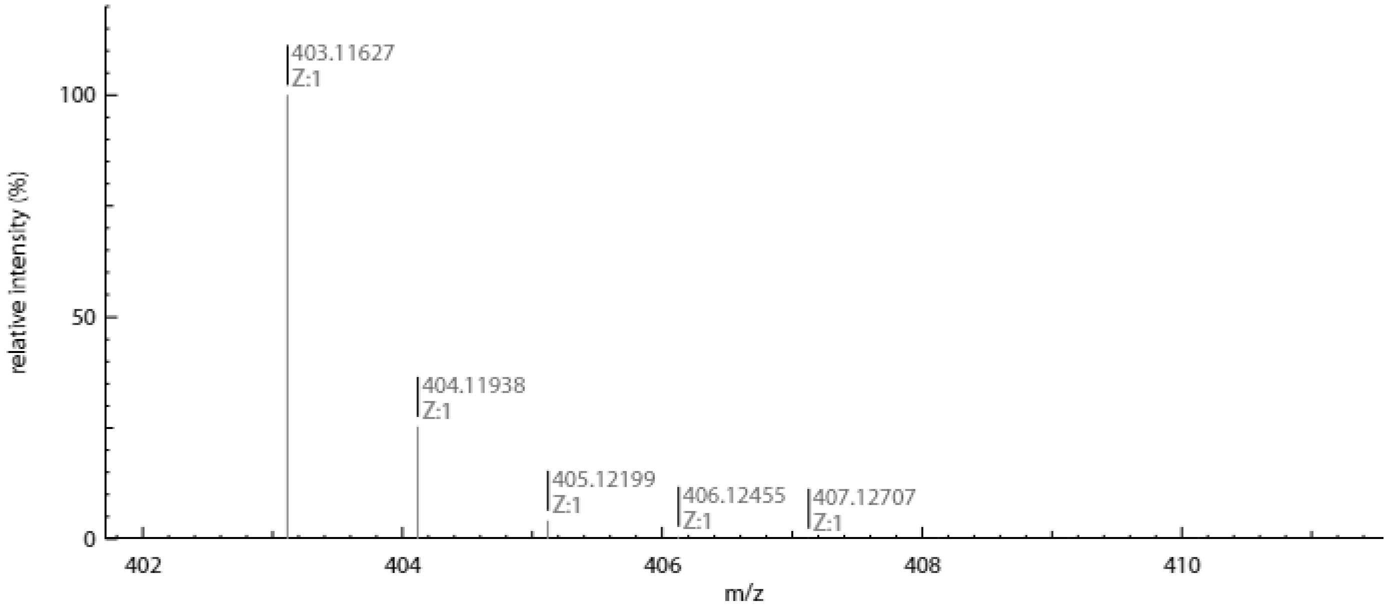

Figure S26: Mass spectrum of N'-(1-benzyl-2-oxo-1,2-dihydro-3H-indol-3-ylidene)-2-chlorobenzohydrazide (5h):

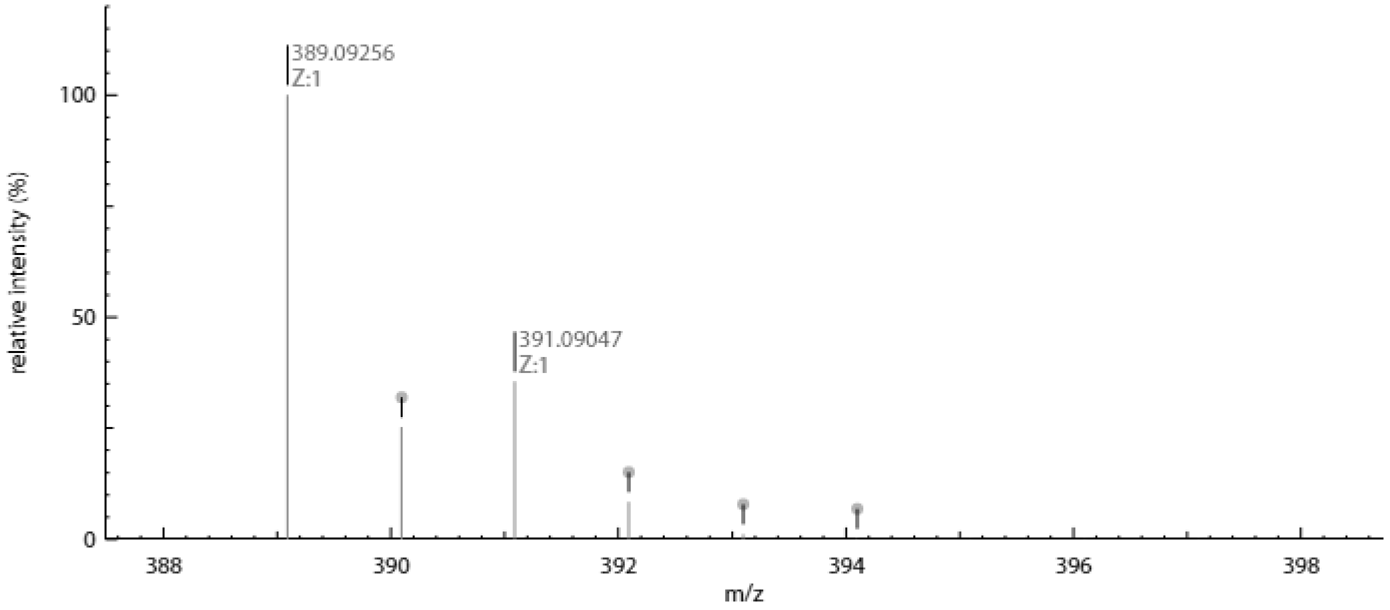

Figure S27: Mass spectrum of N'-(1-benzyl-2-oxo-1,2-dihydro-3H-indol-3-ylidene)benzohydrazide (5i):

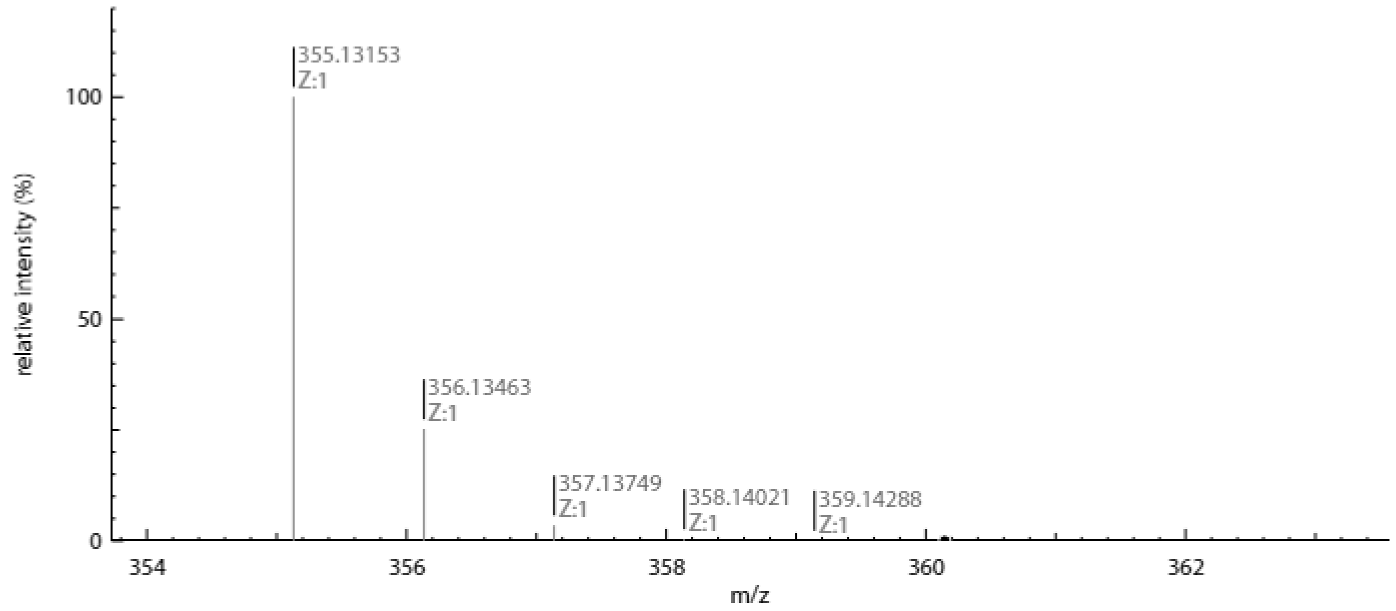

Supplement: Supplementary file 1 [file DataSheet1.PDF]
